# Comparative genomics, pangenomics, and phenomic studies of *Pectobacterium betavasculorum* strains isolated from sugar beet, potato, sunflower, and artichoke: insights into pathogenicity, virulence determinants, and adaptation to the host plant

**DOI:** 10.3389/fpls.2024.1352318

**Published:** 2024-03-21

**Authors:** Maria Borowska-Beszta, Magdalena Smoktunowicz, Daria Horoszkiewicz, Joanna Jonca, Michal Mateusz Waleron, Jan Gawor, Adriana Mika, Tomasz Sledzinski, Krzysztof Waleron, Malgorzata Waleron

**Affiliations:** ^1^Laboratory of Plant Protection and Biotechnology, Intercollegiate Faculty of Biotechnology, University of Gdansk and Medical University of Gdansk, Gdansk, Poland; ^2^Department of Pharmaceutical Microbiology, Faculty of Pharmacy, Medical University of Gdansk, Gdansk, Poland; ^3^DNA Sequencing & Synthesis Facility, Institute of Biochemistry & Biophysics, Polish Academy of Sciences, Warsaw, Poland; ^4^Department of Pharmaceutical Biochemistry, Faculty of Pharmacy, Medical University of Gdansk, Gdansk, Poland

**Keywords:** *Pectobacterium betavasculorum*, genomics, pangenomics, pathogenomics, fatty acid composition, host specificity, adaptation capacity, sugar beet

## Abstract

**Introduction:**

Bacteria of genus *Pectobacterium*, encompassing economically significant pathogens affecting various plants, includes the species *P. betavasculorum*, initially associated with beetroot infection. However, its host range is much broader. It causes diseases of sunflower, potato, tomato, carrots, sweet potato, radish, squash, cucumber, and chrysanthemum. To explain this phenomenon, a comprehensive pathogenomic and phenomic characterisation of *P. betavasculorum* species was performed.

**Methods:**

Genomes of *P. betavasculorum* strains isolated from potato, sunflower, and artichoke were sequenced and compared with those from sugar beet isolates. Metabolic profiling and pathogenomic analyses were conducted to assess virulence determinants and adaptation potential. Pathogenicity assays were performed on potato tubers and chicory leaves to confirm *in silico* predictions of disease symptoms. Phenotypic assays were also conducted to assess the strains ability to synthesise homoserine lactones and siderophores.

**Results:**

The genome size ranged from 4.675 to 4.931 kbp, and GC % was between 51.0% and 51.2%. The pangenome of *P. betavasculorum* is open and comprises, on average, 4,220 gene families. Of these, 83% of genes are the core genome, and 2% of the entire pangenome are unique genes. Strains isolated from sugar beet have a smaller pangenome size and a higher number of unique genes than those from other plants. Interestingly, genomes of strains from artichoke and sunflower share 391 common CDS that are not present in the genomes of other strains from sugar beet or potato. Those strains have only one unique gene. All strains could use numerous sugars as building materials and energy sources and possessed a high repertoire of virulence determinants in the genomes. *P. betavasculorum* strains were able to cause disease symptoms on potato tubers and chicory leaves. They were also able to synthesise homoserine lactones and siderophores.

**Discussion:**

The findings underscore the adaptability of *P. betavasculorum* to diverse hosts and environments. Strains adapted to plants with high sugar content in tissues have a different composition of fatty acids in membranes and a different mechanism of replenishing nitrogen in case of deficiency of this compound than strains derived from other plant species. Extensive phenomics and genomic analyses performed in this study have shown that *P. betavasculorum* species is an agronomically relevant pathogen.

## 1 Introduction

*Pectobacterium*, necrotrophic phytopathogenic rods, are a common causative agent of soft rot and blackleg diseases in agricultural and ornamental plants. They possess a wide range of plant cell-wall-degrading enzymes, which are their main virulence factor (Toth et al., 2021). Economic losses caused by these bacteria classified them in the 10th position out of the top 10 most important bacterial phytopathogens in the world (Mansfield et al., 2012).

Among *Pectobacterium* species, *P. betavasculorum* is considered a causative agent of vascular necrosis in sugar beets (Thomson, 1981; Rastgou et al., 2022). Bacteria from this species were first isolated from sugar beets with soft rot symptoms in the USA in 1972 (Thomson et al., 1977). In 1981, they were delineated as a new subspecies of *Erwinia carotovora* subsp. *betavasculorum* (Thomson, 1981). Previously, this species was thought to have a narrow host range restricted to sugar beet. However, further studies show that, in addition to beets, *P. betavasculorum* species can infect artichokes, carrots, cucumber, potatoes, sweet potatoes, radishes, sunflowers, squash, tomato, zucchini, and chrysanthemum (Whitney and Duffus, 1986; Harveson et al., 2009; Khan and Siddiqui 2020; Saleh et al., 1996).

**Figure 1:**
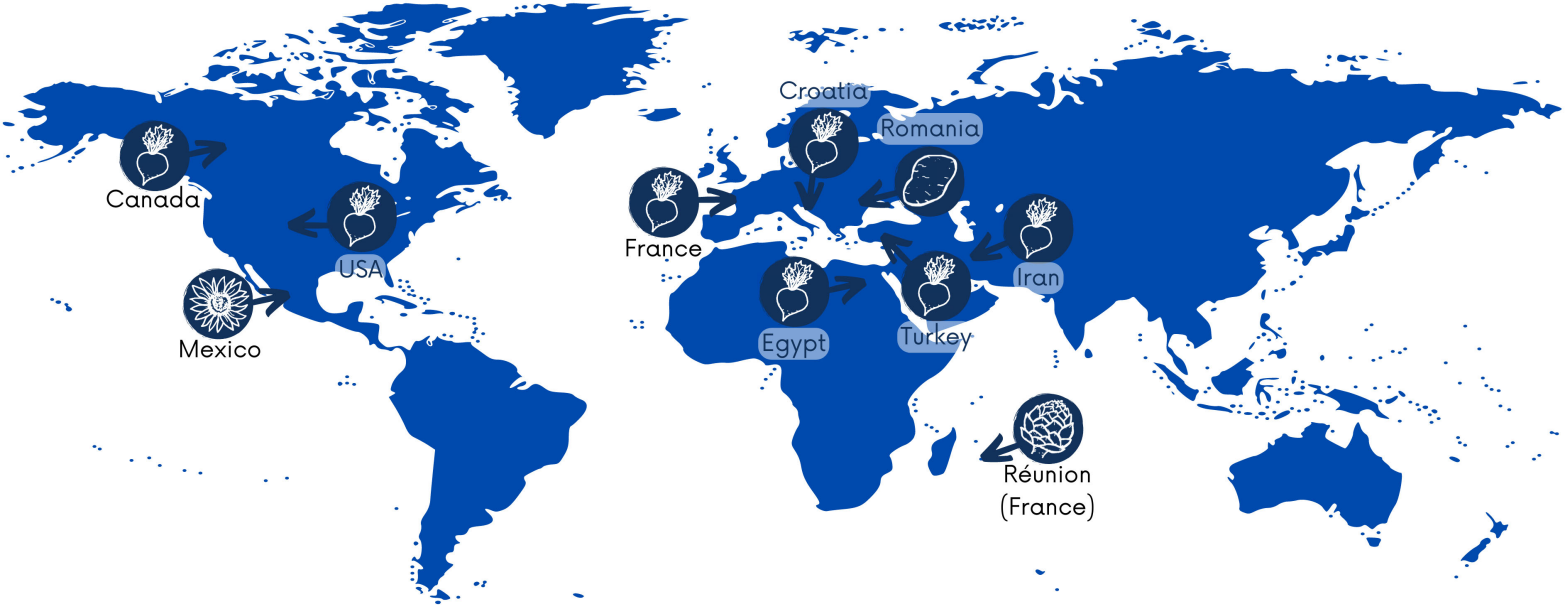
Distribution map of *P. betavasculorum*, indicating isolation country and host plant.

*P. betavasculorum* causes severe losses in sugar crops globally, and these bacteria have been reported on four continents, including Asia, Africa, Europe, and North America. According to the Centre for Agriculture and Bioscience International Organization (CABI) https://www.cabi.org/, as of 31 March 2021, the occurrence of *P. betavasculorum* had been reported in nine countries, such as the USA (Thomson, 1981), Egypt (Saleh et al., 1996), the French island La Reunion and Mexico (Gardan et al., 2003), Croatia (Ðermić, 2010), Iran (Rezaei and Taghavi, 2010; Nedaienia and Fassihiani, 2011; Baghaee-Ravari et al., 2011; Rezaee Danesh et al. 2021), Canada (Gilbert et al., 2015), Turkey (Ozturk et al., 2019), and France and Romania (Portier et al., 2020) (**Figure 1**). They are also present in Russia. In 2022, gene sequences from five *P. betavasculorum* strains isolated in Russia have been deposited in the GenBank database under accession numbers (OP593502–OP593506).

The industrial production of crops like potatoes, sugar beets, and sunflowers is essential for agriculture. According to the Food and Agriculture Organization (FAO) data, in 2021, a total of 376 million tons of potatoes were produced worldwide, and the total world production of sugar beets was 252.9 million tons. (FAOSTAT September 2023). These crops are vital for human consumption and play a crucial role in the food market. They are also extensively used in the biotechnological industry. For example, sugar beets, sucrose, potatoes, starch, and the waste from their production are used to produce biodegradable polymers (including the most popular polylactide, PLA), bioethanol, hydrogen, or biogas (Tomaszewska et al., 2018). Sunflowers have been recognized as functional foods or nutraceuticals, feedstock to produce biodiesel and bioplastics (Geneau-Sbartaï, 2008), and edible membranes, which serve as a packaging material in the food industry (Petraru and Amariei, 2022).

This intricate web of dependencies may be easily disrupted in case of a sudden decrease in crop yield caused by the instability of the Earth’s climate due to global warming. Plant development and crop yield depend on weather conditions during the entire vegetation period. From 1990 to 2020, there has been an increase of more than 0.5°C in average global temperature and more than 1.5°C in Europe, Asia, and Africa (FAOSTAT September 2023). This continuous increase in temperature results in water limitation, increasing soil salinity, and reduced nutrient availability, which negatively affect plant growth and reduce their resistance to pathogens such as viruses, nematodes, fungi, and bacteria. Microorganisms with increased resistance to draught conditions especially pose a significant threat to weakened plants.

Despite widespread occurrence, little is still known about *P. betavasculorum* species. One reason may be that, so far, no rapid detection and identification methods have been developed. Unfortunately, the PCR method commonly used to detect bacteria of the genus *Pectobacterium* using primers developed by Darrasse et al. (1994) is not useful in the case of *P. betavasculorum.* The primers were designed based on the *pelY* gene sequence, which is not present in the genomes of *P. betavasculorum*. The identification of this species is based on PCR-RFLP (Toth et al., 2001; Waleron et al., 2002), AFLP (Avrova et al., 2002), sequencing analyses of the 16S rRNA gene (Kwon et al., 1997; Hauben et al., 1998), and housekeeping genes (Gardan et al., 2003; Ma et al., 2007; Waleron et al., 2018; Portier et al., 2020). The difficulty of identification undoubtedly results in major underestimation of this pathogen’s significance and global prevalence.

By November 2023, in the GenBank database, only two genomic sequences are available for *P. betavasculorum* strains, NCPPB2793 (JQHM00000000) and NCPPB2795T (JQHL00000000), both isolated from sugar beet in the USA. It further emphasizes the need for thorough research in order to improve our understanding of this little-studied plant pathogen. Therefore, in the present study, we undertook genomic sequencing of four strains, CFBP1520 isolated from a sunflower in Mexico, SF142.2 originating from artichoke from the French island of La Reunion, CFBP3291 isolated from a potato in Romania, and strain Ecb168, derived from sugar beet in the USA.

The aim of this study was the comprehensive genetic and phenotypic characterization of the *P. betavasculorum* strains, which causes diseases of various plants that are an important part of the food market and are also used as raw materials in biotechnology.

The polyphasic analysis was performed to gain the broadest possible understanding of the biology of this underestimated *Pectobacterium* species. Extensive phenomic and genomic analyses were applied to verify their adaptive capabilities.

A special emphasis was placed on testing their potential to infect plants with high sugar or lipid content. It is worth highlighting that *P. betavasculorum* has a broad host range and may infect other plants than those from which it was originally isolated. It might be important for agriculture, especially in the era of progressive climate change, as such bacteria may be naturally more resistant to drought conditions and readily infect weakened plants. It may, in turn, lead to substantial losses in the crop industry. This is a significant concern, as no effective preventive and eradication methods exist for this pathogen. Thus, these bacteria may easily spread to new niches due to the intensive international trade of seeds, horticultural and ornamental plants, and plant-originated foods.

## 2 Methods

### 2.1 Bacterial strains

The 14 P*. betavasculorum* strains used in this study are listed in **Table 1**, and the reference *Pectobacterium* strains used for comparison are specified in **Supplementary Table S1**. The strains of various *Pectobacterium* species and *Chromobacterium violaceum* CV026 are a part of the *Pectobacterium* culture collection at the Intercollegiate Faculty of Biotechnology, Gdansk, Poland. *Pectobacterium* strains were maintained on the Crystal Violet Pectate (CVP) medium (Hélias et al. 2012), and *Chromobacterium violaceum* CV026 was cultivated on the Mueller–Hinton (MH) medium at 28°C. All strains were stored in 40% glycerol at −80°C.

**Table 1:** Host plant, geographical origin, year of isolation, ERIC profile, *recA* PCR-RFLP profile, and pathogenicity of the studied *P. betavasculorum* strains.

Strain	Host plant	Geographic origin	Year of isolation	ERIC profile	recA PCR-RFLP Profile*	The mean diameter of macerated potato tissue area [cm]	The average rotted area on chickory leaf [cm^2^]s
**LA 129=Ecb 168**	**Sugar beet**	**Monterey, California USA**	**1970s**	**I**	**21**	**3.55 ± 0.8**	**15.5 ± 9.9**
CFBP2121=NCPPB2794	Sugar beet	Moses Lake, Washington, USA	1974	II	21	3.6 ± 2.8	35.3 ± 18.7
343	Sugar beet	Kern County, California, USA	1970s	II	21	1.47 ± 0.1	9.94 ± 0.4
350	Sugar beet	Arizona, USA	1970s	II	21	5.10 ± 0.5	17.27 ± 0.1
0342=NCPPB3075	Sugar beet	Imperial County, California, USA	1970s	II	21	4.13 ± 0.1	65.67 ± 33.3
**CFBP1520**	**Sunflower**	**Mexico**	**–**	**III**	**21**	**4.54 ± 0.8**	**155.43 ± 0.1**
**SF142.2**	**Artichoke**	**La Reunion**	**1986**	**III**	**21**	**1.27 ± 0.4**	**43.44 ± 11.7**
307	Sugar beet	USA	–	II	21	6.44 ± 1.0	155.43 ± 0.1
**0341=NCPPB2793**	**Sugar beet**	**Belridge, California, USA**	**1974**	**II**	**21**	**9.97 ± 0.1**	**31.71 ± 5.44**
**NCPPB2795^T^=CFBP2122 = 307**	**Sugar beet**	**Kern Country, California, USA**	**1972**	**II**	**21**	**2.97 ± 0.4**	**22.52 ± 16.1**
**CFBP3291=LMG2398**	**Potato**	**Romania**	**1968**	**II**	**nt**	**1.4 ± 0.1**	**11.54 ± 5.0**
CFBP5531	Sugar beet	France, Aube, Avant-Les-Marcilly	1997	II	nt	1.36 ± 0.3	26.62 ± 9.1
CFBP5536	Sugar beet	France, Aube	1996	II	nt	1.7 ± 0.1	30.75 ± 0.8
CFBP5540	Sugar beet	France	2000	II	nt	1.52 ± 0.3	33.31 ± 7.6

*The numbers of recA PCR-RFLP profiles according to Waleron et al. (2002); nt, not tested.*P. betavasculorum* strains for which genomic sequences were analysed were marked in bold.

### 2.2 Phenotypic characterization

#### 2.2.1 Biochemical and physiological tests

Biochemical tests were performed with the BIOLOG GENIII plates (Biolog Inc., Hayward, CA, USA) according to the manufacturer’s instructions, using inoculation fluid A. Briefly, overnight cultures of bacteria on MHII plates were resuspended in 0.85% saline solution. A volume of 100 μl of bacterial suspension was added into each of the microplate wells. The change in color of the wells was evaluated with the naked eye. The reference or type strains of *Pectobacterium* species listed in **Supplementary Table S1** were used for comparison. Additionally, the production of acid from α-methyl glucoside or reducing substances from sucrose, utilization of citrate, dulcitol and dextrose as the only carbon source, fermentation of 10 sugars, inulin, mannitol and sorbitol and production of 17 enzymes using the DIATABS™, Diagnostic Tablets (RoscoDiagnostica A/S, Taastrup, Denmark) were assessed.

#### 2.2.2 Growth assays, adaptation to various environmental conditions, and metabolic activity measurements

The growth of *P. betavasculorum* strains in a microtiter plate was determined by absorbance (OD) measurement at 600 nm each hour for 72 h of incubation at 28°C using a microplate reader InfiniteM200Pro (Tecan, Männedorf, Switzerland).

The assays were performed in 96-well titration plates. The 200-μL of tryptic soy broth (TSB) medium with various pH, salinity, and PEG concentrations was inoculated with 5 μL of bacterial suspension with an optical density of 0.5 McF. The plates were incubated with shaking at 28°C. The absorbance readings at 600 nm were made after 0, 6, 24, and 48 h of incubation, using the Infinite M200 Pro (Tecan, Männedorf, Switzerland). The experiments with two replicates were performed twice. The effect of temperature on the growth of *P. betavasculorum* strains was investigated in the TSB medium. Plates were incubated with shaking at 15°C, 20°C, 28°C, and 37°C. The effect of pH on bacterial growth was studied in a TSB medium under pH values of 4, 5, 6, 7, 8, 9, 10, and 11, respectively. The ability to grow in various salinity conditions was conducted in TSB medium supplemented with 0 g L^−1^, 10 g L^−1^, 20 g L^−1^, 30 g L^−1^, 40 g L^−1^, 50 g L^−1^, 60 g L^−1^, 70 g L^−1^ NaCl, and 80 g L^−1^. The tolerance for limited water availability was estimated in TSB medium supplemented with 0.0 g L^−1^, 50.0 g L^−1^, 75.0 g L^−1^, 100.0 g L^−1^, 200.0 g L^−1^, 300.0 g L^−1^, 400.0 g L^−1^, and 500.0 g L^−1^ of polyethylene glycol (PEG).

The metabolic activity of *P. betavasculorum* strains cultivated in media with different sugars and plant extracts was assessed by the polypyridyl complex of Ru(II) method as described in (Jońca et al., 2021). The following media were used: M63 medium supplemented with 0.5% solutions of four polyols, namely, arabitol, erythritol, sorbitol, and xylitol,; eight monosaccharides, namely, ribose, arabinose, xylose, glucose, fructose, fucose, mannose, rhamnose, nine disaccharides, sucrose, lactose, maltose, isomaltose, melibiose, cellobiose, turanose, trehalose, pallatinose; one trisaccharide–raphinose; three polymers and starch such as (consisting of numerous glucose units), inulin (composed mainly of fructose units—fructans), pectin (pectic polysaccharide, rich in galacturonic acid), and taurine (non-protein amino acid). Moreover, M63 medium supplemented with 10% of extracts obtained from plants such as sugar beet, fodder beet, calla lily, blackberry, pitaya, ground cherry, black nightshade, orchid, viviparous, and *Arabidopsis thaliana* were used for the experiments. Plant extracts were prepared according to the procedure previously described by Jońca et al. (2021). Absorbance was read at 600 nm and fluorescence at 480 nm, each hour for 72 h of incubation at 28°C using a microplate reader InfiniteM200Pro (Tecan, Männedorf, Switzerland). The experiment was performed with three replicate samples.

#### 2.2.3 Antibiotic susceptibility assay

The antibiotic susceptibility of *P. betavasculorum* strains was tested by a standard disk diffusion method. Antibiotic disks containing ampicillin 10 µg, erythromycin 15 µg, gentamicin 250 µg, kanamycin 30 µg, streptomycin 10 µg, and tetracycline 15 µg (Biomaxima, Gdansk, Poland) were used. Briefly, 100 µL of bacterial suspension was spread on a Mueller–Hinton (MH) medium with an optical density of 0.5 McF. After applying the antibiotic disks, the plates were incubated for 24 h at 28°C. Then, the zone of bacterial growth inhibition around the antibiotic disk was assessed. Moreover, the production of beta-lactamases and carbapenemases was determined with the application of chromogenic medium, ESBL (extended-spectrum beta-lactamases), and KPC (*Klebsiella pneumoniae* carbapenemase) plates (Graso Biotech, Owidz, Poland) as described previously (Smoktunowicz et al., 2022).

#### 2.2.4 Plant tissue maceration assays

Pathogenicity of the *P. betavasculorum* strains was assessed for chicory leaves and potato tubers. Tests were performed as described previously (Lee et al., 2013; Smoktunowicz et al., 2022). Briefly, plant leaves and tuber slices were surface sterilized by soaking in 5% (v/v) sodium hypochlorite (NaOCl) and then rinsed thrice with distilled water. Bacterial cultures were grown on CVP medium for 48 h and then harvested and resuspended in Ringer solution to an approximate cell density of 10^8^ CFU mL^−1^. A small incision was made on each chicory leaf using a pipette tip, and 25 μL of each bacterial suspension was inoculated into the damaged section. Pipette tips containing 50 μL of each bacterial suspension were driven into the flesh of the surface disinfected potato slices. Plants were placed in sterilized plastic boxes with sufficient moisture and stored at 28°C for several days. Plants inoculated with Ringer solutions were used as a negative control. The experiments were repeated three times, each with three replicate samples.

#### 2.2.5 PCWDEs, N-acyl homoserine lactone siderophore and auxin production

The activity of PCWDEs was assessed as described previously (Smoktunowicz et al., 2022; Andro et al., 1984; Miller, 1972; Tindall et al., 2014; Himpsl and Mobley, 2019). Briefly, for polygalacturonase assay, isolates were incubated on a solid M63 medium (Miller, 1972) containing polygalacturonic acid. After 48 h incubation at 28°C, plates were stained by flooding with 10% (w/v) copper acetate, which forms a blue complex with the polymer, leaving clear haloes around colonies that produce pectolytic enzymes. Cellulase activity was assessed on a medium containing 0.1% (w/v) carboxymethylcellulose (CMC). After 48 h of incubation at 30°C, the plates were stained with an aqueous 0.1% (w/v) Congo red solution for 1 h at room temperature and washed with 1 M NaCl. Cellulase-producing colonies formed “halo” zones. Protease, lipase, and oligo-1,6-glucosidase activities were assessed on skimmed milk, egg yolk agar, and starch agar, respectively (Tindall et al., 2014). Siderophore production was assessed with chrome azurol S (CAS) agar plate assay (Himpsl and Mobley, 2019). The diameter of “halo” zones around bacterial colonies was measured in each case. Experiments were performed in two repetitions, and the means were calculated for each strain. Gelatinase production was assayed with the standard gelatin stab method (Tindall et al., 2014). Liquefaction of the medium was assessed after 1 week of incubation. Malonate utilization as a sole carbon source was evaluated using the malonate broth (Tindall et al., 2014). A shift in the pH indicator color from green to dark violet indicated malonate utilization.

The capability of the strains to produce N-acyl homoserine lactone (AHL) was assessed using *Chromobacterium violaceum* CV026 as a biosensor, as described by Ravn et al. (2001). Briefly, tested strains were streaked on the LA plates and then incubated overnight at 28°C. After that time, *C. violaceum* CV2026 was streaked on the plates in parallel. Plates were then once again incubated at 30°C overnight. After that time, the plates were evaluated for the purple violacein pigment that is produced by *C. violaceum* in the presence of AHLs.

The auxin production by *P. betavasculorum* strains was assessed by confirming with a colorimetric assay using the Salkowski reagent method (Gang et al., 2019). Strains were cultivated overnight at 30°C in TSB medium supplemented with 0.5 g of tryptophan. After centrifugation, 100 µL of supernatant and 100 µL of Salkowski reagent were mixed on a 96-well plate incubated for 30 min in the dark. The color intensity was measured spectrophotometrically at a wavelength of 536 nm. A non-inoculated medium was used as a control. An *Escherichia coli* strain ATCC8339 was used as a positive control, while *ΔtnaA* deletion mutant, which lost l-tryptophan degradation activity (Shimazaki et al., 2012), was used as a non-producing auxins control.

### 2.3 Fatty acids analysis

Total lipids were extracted from bacteria suspension in Ringer solution, using a mixture of chloroform and methanol (2:1 v/v). Lipid extracts were subjected to hydrolysis by 0.5 M KOH. After hydrolysis and washing with water/n-hexane, the n-hexane phase was evaporated to dryness under a stream of nitrogen. 19-Methyl eicosanoate was used as an internal standard (IS). Free fatty acids (unesterified FAs) were methylated with 10% boron trifluoride reagent in methanol. The fatty acid methyl esters (FAMEs) were analyzed with gas chromatography/electron ionization mass spectrometry (GC-EI-MS). FAMEs were separated in a 30 m×0.25 mm i.d. RTX-MS-5 capillary column (film thickness, 0.25 μm). The column temperature was programmed from 60°C to 300°C at a rate of 4°C/min. The injector temperature and ion source was 300°C and 200°C, respectively. The carrier gas was helium, and the electron energy will be 70 eV.

A standard mix of fatty acids methyl esters (Supelco ^®^ 37 Component FAME Mix. 1890. cis/trans) were used for the identification of the major fatty acids based on retention time. Individual FAs were expressed as percentages of the total FA’s present in the chromatogram.

### 2.4 Genetic characterization

#### 2.4.1 Genomic DNA preparation

Strains of *P. betavasculorum* were stored in 20% glycerol at −80°C. For the preparation of genomic DNA, bacteria were first grown for 48 h on CVP medium (Hélias et al. 2012) at 28°C. A volume of 10 ml of the liquid TSB medium (bioMerieux, Marcy-l’Étoile, France) was inoculated with a single colony from the CVP plate and grown overnight at 28°C with shaking. Obtained cultures were centrifuged for cell harvest for 20 min at 3,200*g* at 4°C and resuspended into TE buffer (10 mM Tris–HCl, pH 7.5, 1 mM EDTA). The DNA was extracted according to the cetyltrimethylammonium bromide (CTAB) protocol (www.jgi.doe.gov, accessed on 5 August 2023). The quantity and quality of the DNA were assessed by the spectrophotometric analysis with a microplate reader InfiniteM200Pro (Tecan, Männedorf, Switzerland) and the 1% agarose gel electrophoresis.

#### 2.4.2 DNA fingerprinting analyses

A total genomic DNA fingerprinting was performed by the repetitive element PCR fingerprinting (rep-PCR) using enterobacterial repetitive intergenic consensus (ERIC) primers (Versalovic et al., 1991). Analysis was performed with BioNumerics V6.6 (http://www.applied-maths.com). The neighbor-joining cladogram was created with a band-based Jaccard coefficient.

#### 2.4.3 Multi-locus sequence analysis

The sequences of 13 housekeeping genes (a*cnA*, 2,692 bp; *gapA*, 1,005 bp; *gyrA*, 2,649 bp; *gyrB*, 2,418 bp; *icdA*, 1,254 bp; *mdh*, 940 bp; *mtlD*, 1,149 bp; *pgi*, 1,644 bp; *proA*, 1,259 bp; *recA*, 1,074 bp; *recN*, 1,662 bp; *rpoA*, 990 bp; and *rpoS*, 1,514 bp) commonly used for multi-locus sequence analysis of *Pectobacterium* (Ma et al., 2007; Waleron et al., 2018), were extracted from genomes of six *P. betavasculorum* strains (listed in **Table 1**). Their sequences were merged and aligned with sequences retrieved from genomes of the type strains of each of *Pectobacterium* species that are available at the GenBank database with using the MUSCLE algorithm with the default settings in Geneious Prime (www.geneious.com).

Phylogenetic analyses were performed on the concatenated data set (20,770 bp) with the Maximum-Likelihood method and the General Time Reversible as the best nucleotide substitution model selected using jModeltest 2.1.9 software. Bootstrapping was executed with 1,000 replications. The gene sequences of *D. solani* IFB0099 (JXRS00000000) were used as an outgroup.

#### 2.4.4 Genome sequencing, assembly, and annotation

The genomes of two *P. betavasculorum* strains, CFBP1520 and SF142.2, were sequenced using the Illumina MiSeq technology (**Table 2**). Quality and adapter trimming of the raw reads was performed using Trimmomatic v0.32 (http://www.usadellab.org/cms/?page=trimmomatic, accessed on 5 August 2023); kmer length and distribution were analyzed using KmerGenie v1.6949 (http://kmergenie.bx.psu.edu/, accessed on 5 August 2023). *De novo* assembling was performed using SPAdes v3.10.1 (http://cab.spbu.ru/software/spades/, accessed on 5 August 2023), Velvet v1.2.10 (https://www.mybiosoftware.com/velvet-1-1-07-sequence-assembler-short-reads.html, accessed on 5 August 2023), and Ray v2.3.1 (http://denovoassembler.sourceforge.net/, accessed on 5 August 2023). The contigs were integrated using CISA v1.3 (http://sb.nhri.org.tw/CISA/en/CISA, accessed on 5 August 2023) software and scaffolded with SSPACE v3.0 (https://github.com/nsoranzo/sspace_basic, accessed on 5 August 2023). The final assembly was evaluated using Quast v4.5 (http://quast.sourceforge.net/quast, accessed on 5 August 2023) software. Another two strains, CFBP3291 and Ecb168, were sequenced in a hybrid mode using the Illumina HiSeq 2000 and Oxford Nanopore platforms (**Table 2**). An Illumina-compatible paired-end sequencing library was constructed using the NEB Ultra II FS Preparation Kit (New England Biolabs, Beverly, USA) according to the manufacturer’s instructions. The library was sequenced using an Illumina MiSeq platform (Illumina, San Diego, CA, USA) with 2 × 300 paired-end reads using v3 600-cycle sequencing kit. Sequence quality metrics were assessed using FASTQC (http://www.bioinformatics.babraham.ac.uk/projects/fastqc/), and quality was trimmed using fastp (Chen et al., 2018). Prior to long-read library preparation, genomic DNA was sheared into ~30 kb fragments using 26G needle, followed by size selection using a Short Read Eliminator kit (Circulomics, Baltimore, MD, USA). Five micrograms of the recovered DNA was taken for 1D library construction using SQK-LSK109 kit, and 0.8 µg of the final library was loaded into R9.4.1 flow cell and sequenced on GridION sequencer (Oxford Nanopore Technologies, Oxford, UK). Raw nanopore data was basecalled using Guppy v5.0.7 in super accuracy mode (Oxford Nanopore Technologies, Oxford, UK). After quality filtering using NanoFilt (De Coster et al., 2018) and residual adapter removal using Porechop (https://github.com/rrwick/Porechop), the obtained dataset was quality checked using NanoPlot (De Coster et al., 2018). Long nanopore reads were then assembled in hybrid mode using Unicycler (Wick et al., 2017). The remaining ambiguities in the genome assemblies were verified by the PCR amplification of DNA fragments, followed by Sanger sequencing with an ABI3730xl Genetic Analyzer (Life Technologies, Carlsbad, CA, USA) using BigDye Terminator Mix v. 3.1 chemistry (Life Technologies, Carlsbad, CA, USA). All of the possible sequence errors and miss-assemblies were further manually corrected using Seqman software (DNAStar, Madison, WI, USA) to obtain the complete nucleotide sequence of bacterial genomes). *novo* assembling was performed using Flye v 2.9.1 (https://github.com/fenderglass/Flye, accessed on 5 August 2023) and Unicycler v 0.5.0 (https://github.com/rrwick/Unicycler, accessed on 5 August 2023). The final assembly was evaluated using Quast v4.5 (http://quast.sourceforge.net/quast, accessed on 5 August 2023) software.

**Table 2:** Genomic features of *Pectobacterium betavasculorum* strains according to GenBank database.

Strain/attribute	CFBP1520	SF142.2	CFBP3291	Ecb168	NCPPB2793	NCPPB2795^T^
Genome Accession no	JAODTE00000000	JANIMZ00000000	CP129215	CP118484	JQHL00000000	JQHM00000000
Genome size (bp)	4,931,193	4,925,866	4,873,649	4,764,233	4,675,732	4,685,213
DNA G+C (%)	51.2	51.2	51.2	51.0	51.0	51.2
DNA scaffolds	63	48	1	1	115	93
Total genes	4,581	4,554	4,587	4,329	4,126	4,321
Total CDSs	4,448	4,457	4,465	4,218	3,982	4,225
Protein coding genes	4,324	4,350	4,271	4,103	4,077	4,118
RNA genes	97	97	99	99	96	96
Pseudo genes	124	107	194	115	48	107
CRISPR repeats	3	3	3	3	5	3
Plasmid (size -bp, Accession no)	nd	nd	pPB3291 23636bp, 46.7%GC CP129216	nd	nd	pB2795 23736bp, 46.7%GC JQHM00000026

Annotation was performed by the NCBI PGAPpipeline (https://www.ncbi.nlm.nih.gov/genome/annotation_prok/, accessed on 5 August 2023) and RAST (Rapid Annotation using Subsystem Technology) (http://rast.nmpdr.org/, accessed on 5 August 2023). The genomic sequence was additionally annotated with BlastKOALA (KEGG Orthology And LinksAnnotation) (https://www.kegg.jp/blastkoala/, accessed on 5 August 2023).

#### 2.4.5 Genomic analyses

Comparative genomics was performed with the use of Pathway Tools Software v27.0 (http://bioinformatics.ai.sri.com/ptools/, accessed on 14 August 2023), Bacterial Pangenome Analysis (BPGA) pipeline (Chaudhari et al., 2016) (https://iicb.res.in/bpga/, accessed on 13 September 2023), and a GET_HOMOLOGUES software package (Contreras-Moreira and Vinuesa, 2013). The obtained results were subjected to the BLAST analysis against the UniProt protein database (https://www.uniprot.org/, accessed on 13 September 2023). The entire set of genes identified in all *P. betavasculorum* genomes was recognized as a pangenome. The core of the pangenome consists of the gene families that were present on all six analyzed genomes. While the accessory genome contains gene families that were present in at least two up to five of six analyzed *P. betavasculorum* genomes. The unique genes were only found in a single genome.

The visual comparison of genome homology was done with BRIG (BLAST Ring Image Generator) (http://sourceforge.net/projects/brig, accessed on 5 August 2023) with the default settings (Alikhan et al., 2011). The genomes of *P. betavasculorum* NCPPB2795^T^ (JQHM00000000) and *P. betavasculorum* NCPPB2793 (JQHL00000000) have been included for genomic comparison.

The phylogenomic analysis based on the core proteins sequence comparison was done using PhyloPhlAn computational pipeline (https://huttenhower.sph.harvard.edu/phylophlan, accessed on 4 August 2023). The core protein analysis was performed using the sequences of other *Pectobacterium* members available on GenBank and the phylophlan library of 400 conserved proteins of Prokaryotes and Archea. The sequence of *Duffyella gerundensis* AR (CP073262) was used as an outgroup. The bootstrap consensus was inferred from 1,000 replicates. To establish the relationship between *P. betavasculorum* strains isolated from different plant species, the ML tree based on 3,573 core genes per genome (3,588,885 bp per genome) have been created with using the EDGAR platform for comparative genomics (https://edgar3.computational.bio.uni-giessen.de/cgi-bin/edgar_login.cgi). The calculation of the average nucleotide identity (ANI), the average amino-acid identity (AAI), and the Pairwise Percentage of Conserved Proteins (POCP) was performed using the EDGAR pipeline (Dieckmann et al. 2021). To evaluate the assignment of the studied strains to species, *in silico* and the average nucleotide identity (ANI) were calculated between the genomes of *P. betavasculorum* and the genomes of the other type or reference strains of the members of *Pectobacteriaceae* family with using g JSpeciesWS: a web server (Richter et al., 2016).

The presence of ICE and IME elements in the *P. betavasculorum* genomes was investigated using ICEfinder, a web-based tool (https://bioinfo-mml.sjtu.edu.cn/ICEfinder/ICEfinder.html, accessed on 20 September 2023).

The biosynthetic gene clusters encoding secondary metabolites related to the synthesis of phytotoxins, antibiotics, and antimicrobial compounds were predicted using antiSMASH 4.0 (Blin et al., 2023) (https://antismash.secondarymetabolites.org/#!/about, accessed on 20 September 2023).

### 2.5 Statistical analyses

Statistical significance of the pathogenicity tests and phenotypic assays was assessed using independent Student’s t-tests and one-way analysis of variance (ANOVA). A *post-hoc* analysis was performed using Student’s t-test with a base mean as a reference. Statistical significance was assumed if p-value < 0.05. The statistical analysis was done using R software for Windows version 4.1.2 (R Foundation, Vienna, Austria) and the following packages: ComplexHeatmap (Gu et al., 2016), ggplot2 (Wickham, 2009), and ggpubr (Kassambara, 2023).

## 3 Results

### 3.1 Phenotypic characterization

*P. betavasculorum* strains are Gram-negative, motile, non-sporulating, and facultative anaerobic bacteria that belong to the order *Enterobacteriales* within the class of *Gammaproteobacteria*. Cells are rod shaped with a length of approximately 2 μm in the exponential growth phase. *P. betavasculorum* strains produce pits on a crystal violet pectate medium (**Supplementary Figure S1**). All studied *P. betavasculorum* strains were able to macerate potato tuber and chicory leaf tissue efficiently (**Table 1**).

All of the 14 tested *P. betavasculorum* strains (**Table 1**) could perform glucose, maltose, mannose, rhamnose, raffinose, sucrose, d-xylose, and mannitol fermentation. Additionally, all tolerated salinity was up to 4% NaCl. What is more, all strains exhibited enzyme activity of α- and β-glucosidase, α- and β-galactosidase, β-glucuronidase, ornithine decarboxylase, arginine dihydrolase, tryptophan deaminase, and urease, but not lysine decarboxylase and β-xylosidase. They do not produce acid from α-methyl glucoside, lactose, trehalose, adonitol, sorbitol, or reducing substances from sucrose. In addition, all strains were sensitive to ampicillin, tetracycline, gentamicin, kanamycin, and streptomycin but resistant to erythromycin. None of the tested strains exhibited resistance to beta-lactamases or carbapenems (**Supplementary Figure S2**, **Supplementary Table S1**).

Metabolic profiling showed that all tested *P. betavasculorum* strains could use as sole carbon sources the following compounds: dextrin, α-D-glucose, D-galactose (except type strain NCPPB2795^T^), gentiobiose, D-fructose, D-maltose, D-mannose, L-rhamnose, D-cellobiose, D-mannitol, myoinositol, glycerol, D-sorbitol, D-glucose-6-PO_4_, sucrose, D-trehalose, D-turanose, β-methyl-D-glucoside, D-salicin, N-acetyl-D-glucosamine, D-aspartic acid, L-aspartic acid, L-glutamic acid, D-gluconic acid, L-galactonic acid lactone, acetic acid, bromosuccinic acid, formic acid, L-lactic acid, L-malic acid, mucic acid, D-saccharic acid, and pectin. However, they were not able to utilize stachyose, D-raffinose, α-D-Lactose, D-melibiose, D-Fucose, L-Fucose, and D-arabitol, D-serine, L-histidine, L-pyroglutamic acid, glucuronamide, quinic acid, p-hydroxyphenyl acetic acid, citric acid, D-malic acid, γ-aminobutyric acid, N-acetyl-β-D-mannosamine, N-acetyl-D-galactosamine, N-acetyl neuraminic acid, 3-methyl glucose, inosine, D-serine, gelatin, L-arginine, D-lactic acid methyl ester, D-malic acid, Tween 40, β-hydroxy-D l-butyric acid, α-keto butyric acid, propionic acid, sodium bromide. In addition, all *P. betavasculorum* strains could grow at pH 6, 1% sodium chloride and 4% sodium chloride (except strain NCPPB2793), and 1% sodium lactate, and were resistant to troleandomycin, rifamycin SV, lincomycin, niaproof 4, vancomycin, tetrazolium violet, and tetrazolium blue. Only two strains, NCPPB 2794 and NCPPB 3075, did not grow at pH 5 and in 4% NaCl, while the rest did. All tested strains were sensitive to fusidic acid, D-serine, minocycline, lincomycin, nalidixic acid, guanidine HCl, lithium chloride, and potassium tellurite (**Supplementary Table S2**).

Analysis of biochemical properties showed that *P. betavasculorum* strains CFBP3291, CFBP1520, and SF142.2 isolated from potato, sunflower, and artichoke differ in metabolic activity from sugar beet strains, as they cannot utilize 3-methyl-glucose. What is more, the strains CFBP1520 and SF142.2 also cannot utilize lactose and raffinose. Unlike sugar beet strains, they could use α-hydroxybutyric acid as their only carbon source. In addition, only these two strains are resistant to aztreonam and nalidixic acid. It should be noted that sugar beet strains differ from one another. For example, strain Ecb168, like the sunflower and artichoke strains, does not use lactose and raffinose. Unlike all other strains, this strain did not use inosine, glycyl-L-proline, or L-serine.

**Figure 2:**
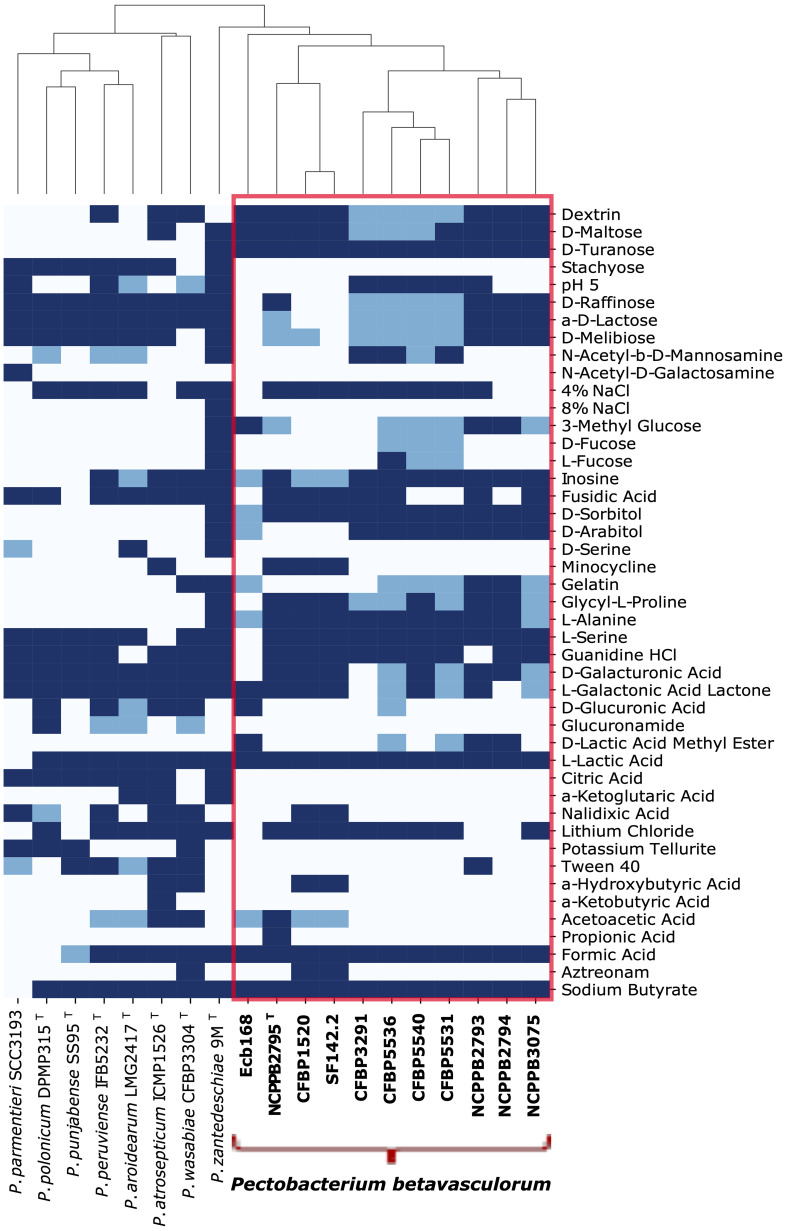
Phenotypic characters that differentiate ten *P. betavasculorum* strains, IFB5271, CF142.2. CFBP3291, Ecb168, CFBP5531, CFBP5536, CFBP5540, NCPPB2795^T^, NCPPB2793, NCPPB3075, and other closely related species: *P. atrosepticum* ICMP1526^T^, *P. parmentieri* SCC3193, *P. peruviense* IFB5232^T^, *P. polonicum* DPMP315^T^, *P. punjabense* SS95^T^, *P. wasabiae* CFBP 3304^T^, and *P. zantedeschiae* 9M^T^. Navy color means a positive reaction, blue means a weak activity, and white means an adverse reaction.

In contrast, the type strain, *P. betavasculorum* NCPPB2795^T^, can use propionic acid but does not use D-galactose and is resistant to minocycline. The strain NCPPB2793 was the only one capable of growing with the presence of Tween40, whereas strains NCPPB2794, NCPPB3075, and Ecb168 could not grow in the medium containing 4% NaCl. Moreover, three French strains, CFBP5540, CFBP5536, and CFBP5531, could utilize fucose as a carbon source (**Figure 2**, **Supplementary Table S2**).

The metabolic profiling results of *P. betavasculorum* strains were extended by simultaneous measurements of bacterial growth and metabolism on minimal medium M63 supplemented with 0.5% solutions of various polyols, sugars, polymers, or 10% plant extracts. All strains could use numerous sugars as a building material and energy source. The strains grew efficiently on minimal medium M63 supplemented with 0.5% solutions of ribose, xylose, glucose, trehalose, lactose, maltose, or sorbitol while growing half as fast on media supplemented with cellobiose, palatinose, raffinose, rhamnose, ribose, melibiose, and isomaltose. Feeble growth was observed when 0.5% inulin and turanose were used as a source of organic compounds. No growth ability was observed on media containing 0.5% arabitol, erythritol, xylitol, and taurine (**Supplementary Figure S3**). In addition, *P. betavasculorum* strains can grow on minimal medium containing, as the only carbon source, extracts of various plant species, such as sugar beet, fodder beet, carrot, physalis, dragon fruit, *Arabidopsis thaliana*, and potato tubers. Moreover, poor growth was observed in the case of extracts from blueberry and dragon fruits. None of the strains tested showed the ability to grow in the presence of extracts from ornamental plants like the Calla lily, orchids, viviparous, or black nightshade (**Supplementary Figure S4**).

All tested strains can synthesize signal molecules (acyl-homoserine lactones), siderophores, and plant hormones, which were confirmed using various assays (**Supplementary Figures S5**-**S7**).

A total of 14 tested *P. betavasculorum* strains were able to macerate plant tissue at laboratory conditions. The soft rot disease symptoms were observed on potato tuber slices and chicory leaves inoculated by wounding (**Table 1**).

Bacteria of the species *P. betavasculorum* are highly adaptable to environmental conditions. *P. betavasculorum* strains grew most effectively in the 20°C–28°C temperature range. Reducing the incubation temperature to 15°C resulted in an average of twofold poorer bacterial growth, and the observed difference was statistically significant while increasing the incubation temperature to 37°C resulted in a two- to fivefold decrease in bacterial growth (**Supplementary Figure S8A**). All *P. betavasculorum* strains grow well in salinity (0%–7%) with optimum at 1%–2% NaCl concentration (**Supplementary Figure S8B**). Tested bacteria grow efficiently at pH from 5 up to 11 (**Supplementary Figure S8C**) and tolerate the reduced availability of water up to 10% of PEG concentration in the growth medium (**Supplementary Figure S8D**). Furthermore, *P. betavasculorum* strains are able to grow and were metabolically active in media with different sucrose concentrations from 0% to 20% (**Supplementary Figure S9**).

### 3.2 Fatty acid methyl ester profiling

Fatty acid methyl ester (FAME) profiling was performed for four selected strains, two isolated from sugar beet (Ecb168. NCPPB2795^T^) and two from other plants (SF142. CFBP1520). Strain CFBP3291 isolated from potato was not included, as it is more similar phenotypically and genetically to sugar beet strains. *P. betavasculorum* strains demonstrate the highest amounts of seven fatty acids: palmitic (16:0), palmitoleic acids (16:1), oleic (18:1), pentadecylic acid (15:0), stearic acids (18:0), followed by margaric (17:0) and linoleic acid (18:2n-6) (**Table 3**).

**Table 3:** The total fatty acid composition of *P. betavasculorum* strains NCPPB2795^T^, Ecb168, CFBP1520, and SF142.2.

Fatty Acid	*P. betavsculorum* strain				* Statistically different values [p<0.05]					
	Ecb168	NCPPB2795	CFBP5120	SF142.2	p(B1 vs. B2)	p(B5 vs. B6)	p(B1 vs. B5)	p(B2 vs. B5)	p(B1 vs. B6)	p(B2 vs. B6)
10	0.02 ± 0.01	0.03 ± 0.02	0.02 ± 0.01	0.01 ± 0.000	0.808	0.423	1.000	0.808	0.423	0.423
12	0.28 ± 0.02	0.21 ± 0.08	0.37 ± 0.11	0.35 ± 0.04	0.457	0.849	0.482	0.341	0.207	0.233
14	1.87 ± 0.24	1.48 ± 0.11	3.66 ± 0.32	4.35 ± 0.06	0.269	0.163	0.045*	0.022*	0.009*	0.002*
16	13.5 ± 0.50	11.3 ± 0.11	25.9 ± 0.52	24.5 ± 0.11	0.051	0.124	0.003*	0.001*	0.002*	0.000*
18	4.6 ± 1.43	3.2 ± 0.29	10.7 ± 1.49	10.9 ± 0.27	0.422	0.886	0.100	0.039*	0.050*	0.003*
20	0.35 ± 0.05	0.40 ± 0.03	1.52 ± 0.10	1.39 ± 0.02	0.434	0.317	0.008*	0.008*	0.002*	0.001*
22	0.44 ± 0.04	0.41 ± 0.06	1.60 ± 0.10	1.41 ± 0.20	0.753	0.483	0.008*	0.009*	0.043*	0.043*
24	0.47 ± 0.08	0.51 ± 0.01	1.64 ± 0.05	1.51 ± 0.31	0.669	0.730	0.007*	0.002*	0.083	0.084
26	0.19 ± 0.02	0.15 ± 0.005	0.42 ± 0.01	0.43 ± 0.06	0.161	0.827	0.008*	0.001*	0.063	0.042*
**ECFA**	**21.7 ± 1.50**	**17.7 ± 0.52**	**45.7 ± 2.70**	**44.9 ± 0.67**	**0.124**	**0.781**	0.016*	0.009*	0.005*	0.001*
13	0.18 ± 0.01	0.13 ± 0.02	0.14 ± 0.02	0.13 ± 0.01	0.093	0.808	0.130	0.684	0.072	0.808
15	12.9 ± 1.04	17.2 ± 0.37	2.54 ± 0.05	3.19 ± 0.04	0.060	0.009*	0.010*	0.001*	0.011*	0.001*
17	6.53 ± 0.82	7.78 ± 0.16	1.26 ± 0.06	1.13 ± 0.04	0.274	0.207	0.024*	0.001*	0.023*	0.001*
19	0.10 ± 0.01	0.10 ± 0.000	0.39 ± 0.06	0.32 ± 0.14	0.423	0.708	0.034*	0.035*	0.249	0.257
21	0.10 ± 0.02	0.12 ± 0.000	0.47 ± 0.02	0.43 ± 0.04	0.238	0.499	0.003*	0.002*	0.016*	0.016*
23	0.12 ± 0.02	0.14 ± 0.01	0.47 ± 0.07	0.42 ± 0.02	0.300	0.532	0.034*	0.039*	0.005*	0.004*
25	0.12 ± 0.02	0.11 ± 0.03	0.26 ± 0.01	0.23 ± 0.03	0.764	0.443	0.015*	0.029*	0.076	0.085
**OCFA**	**20.0 ± 1.92**	**25.6 ± 0.49**	**5.5 ± 0.04**	**5.8 ± 0.31**	**0.108**	**0.397**	0.017*	0.001*	0.018*	0.001*
2.6.10-triM 12;0	0.13 ± 0.03	0.10 ± 0.01	0.26 ± 0.000	0.38 ± 0.01	0.360	0.007*	0.033*	0.001*	0.011*	0.002*
5.9.13-triM-14;0	0.05 ± 0.01	0.05 ± 0.01	0.17 ± 0.01	0.23 ± 0.03	1.000	0.198	0.008*	0.008*	0.026*	0.026*
**tri-M-BCFA**	**0.17 ± 0.03**	**0.14 ± 0.01**	**0.43 ± 0.01**	**0.61 ± 0.02**	**0.443**	0.015*	0.014*	0.002*	0.007*	0.002*
iso 12-M-13;0	0.02 ± 0.01	0.01 ± 0.000	0.04 ± 0.01	0.05 ± 0.01	0.423	0.553	0.155	0.095	0.089	0.057
iso 13-M-14;0	0.05 ± 0.000	0.04 ± 0.02	0.11 ± 0.01	0.11 ± 0.02	0.667	1.000	0.027*	0.089	0.095	0.132
iso 14-M-15;0	0.02 ± 0.000	0.03 ± 0.01	0.05 ± 0.01	0.06 ± 0.01	0.423	0.293	0.038*	0.312	0.020*	0.155
iso 15-M-16;0	0.02 ± 0.01	0.02 ± 0.01	0.03 ± 0.000	0.04 ± 0.02	1.000	0.771	0.095	0.095	0.333	0.333
iso 18-M-19;0	0.01 ± 0.000	0.01 ± 0.000	0.04 ± 0.010	0.03 ± 0.01	NT	0.312	0.095	0.095	0.095	0.095
iso 20-M-21;0	0.06 ± 0.01	0.04 ± 0.000	0.21 ± 0.030	0.15 ± 0.01	0.095	0.192	0.036*	0.030	0.030	0.020
iso 21-M-22;0	0.05 ± 0.000	0.05 ± 0.01	0.16 ± 0.01	0.12 ± 0.02	1.000	0.216	0.008*	0.016	0.073	0.089
**iso BCFA**	**0.23 ± 0.005**	**0.20 ± 0.025**	**0.66 ± 0.060**	**0.56 ± 0.025**	**0.360**	**0.248**	0.019*	0.019*	0.006*	0.010*
anteiso 10-M-12;0	0.04 ± 0.01	0.03 ± 0.000	0.06 ± 0.02	0.07 ± 0.01	0.423	0.698	0.349	0.272	0.089	0.057
anteiso 12-M-14;0	0.29 ± 0.04	0.22 ± 0.05	0.60 ± 0.02	0.84 ± 0.03	0.428	0.018*	0.023*	0.022*	0.009*	0.010)
anteiso 14-M-16;0	0.11 ± 0.02	0.15 ± 0.01	0.52 ± 0.02	0.43 ± 0.05	0.130	0.198	0.003	0.002	0.021	0.027
anteiso 16-M-18:0	0.14 ± 0.01	0.15 ± 0.04	0.45 ± 0.000	0.43 ± 0.07	0.924	0.802	0.001	0.021	0.055	0.076
anteiso 20-M-22;0	0.02 ± 0.01	0.01 ± 0.000	0.11 ± 0.000	0.08 ± 0.01	NT	0.020	0.003	0.002	0.014	0.010
anteiso 22-M-24;0	0.04 ± 0.02	0.03 ± 0.01	0.08 ± 0.01	0.11 ± 0.03	0.808	0.443	0.130	0.072	0.155	0.127
**anteiso BCFA**	**0.62 ± 0.05**	**0.58 ± 0.10**	**1.81 ± 0.04**	**1.95 ± 0.12**	**0.740**	**0.384**	**0.003**	**0.007**	**0.009**	**0.012**
**total BCFA**	**1.01 ± 0.07**	**0.91 ± 0.13**	**2.90 ± 0.09**	**3.12 ± 0.17**	**0.568**	**0.371**	**0.004**	**0.006**	**0.007**	**0.009**
**Total SFA**	**42.8 ± 0.49**	**44.1 ± 0.15**	**54.1 ± 2.84**	**53.8 ± 1.15**	**0.117**	**0.923**	**0.058**	**0.072**	**0.013**	**0.014**
14:1 Add	0.49 ± 0.09	0.38 ± 0.05	0.89 ± 0.07	1.35 ± 0.17	0.371	0.125	0.065	0.023	0.045	0.031
14;1	0.02 ± 0.01	0.01 ± 0.000	0.01 ± 0.000	0.03 ± 0.01	0.423	0.095	0.423	1.000	0.293	0.095
16;1	29.25 ± 0.47	31.16 ± 0.77	16.65 ± 2.41	16.40 ± 1.45	0.168	0.937	0.036	0.029	0.014	0.012
18;1	25.61 ± 0.89	22.92 ± 0.44	24.19 ± 1.09	23.26 ± 1.15	0.113	0.618	0.417	0.393	0.247	0.808
19;1	0.12 ± 0.02	0.08 ± 0.000	< LOD	< LOD	0.184	–	–	–		
20;1	0.10 ± 0.01	0.07 ± 0.02	0.25 ± 0.01	0.29 ± 0.03	0.192	0.257	0.006	0.008	0.021	0.017
22;1	0.14 ± 0.01	0.09 ± 0.01	0.25 ± 0.02	0.25 ± 0.06	0.057	0.943	0.020	0.013	0.196	0.119
24;1	**0.2 ± 0.02**	0.17 ± 0.01	0.16 ± 0.04	0.12 ± 0.000	0.088	0.423	0.204	0.804	0.032	0.012
**MUFA**	**55.94 ± 0.34**	**54.9 ± 0.28**	**42.4 ± 3.37**	**41.7 ± 0.42**	**0.134**	**0.859**	**0.057**	**0.066**	**0.001**	**0.001**
18;2n6	1.02 ± 0.15	0.86 ± 0.12	2.84 ± 0.54	3.79 ± 0.64	0.497	0.378	0.082	0.070	0.053	0.047
ARA	0.03 ± 0.00	0.02 ± 0.01	0.06 ± 0.03	0.04 ± 0.000	0.095	0.609	0.423	0.257	1.000	0.038
DGLA	0.08 ± 0.01	0.05 ± 0.000	0.24 ± 0.02	0.21 ± 0.005	0.038	0.232	0.015	0.011	0.003	0.001
AdA	–	–	–	–	–	–	–	–		
**PUFAn6**	**1.1 ± 0.15**	**0.9 ± 0.12**	**3.1 ± 0.59**	**4.0 ± 0.64**	**0.411**	**0.410**	**0.079**	**0.066**	**0.047**	**0.041**
18;3n3	0.16 ± 0.02	0.10 ± 0.01	0.34 ± 0.06	0.49 ± 0.07	0.143	0.220	0.097	0.049	0.042	0.027
EPA	–	–	–	–	–	–	–	–	–	
DHA	–	–	–	–	–	–	–	–	–	
DPAn3	–	–	–	–	–	–	–	–	–	
**PUFAn3**	**0.16 ± 0.02**	**0.10 ± 0.01**	**0.34 ± 0.06**	**0.49 ± 0.07**	**0.143**	**0.220**	**0.097**	**0.049**	**0.042**	**0.027**

The values in bold represent the sum of the amounts of individual fatty acids.NT - not tested.

**Figure 3:**
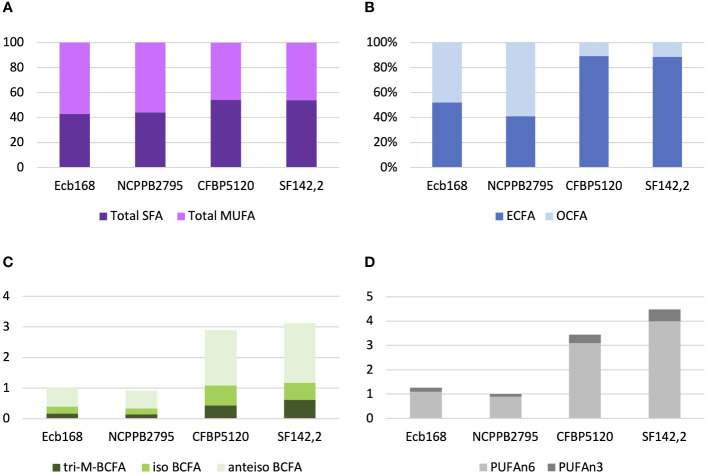
The proportions of the different fatty acid groups present in strains of *P. betavasculorum* isolated from a different host plant. **(A)** The ratio of total saturated fatty acids (SFAs) to monounsaturated fatty acids (MUFAs), **(B)** the proportion of even-chain fatty acids (ECFAs) to odd-chain fatty acids (OCFA), **(C)** the content (%) of polyunsaturated fatty acids (PUFAs) n6 and n3, and **(D)** the content (%) of various branched fatty acids (BCFAs). *P. betavasculorum* strains isolated from sugar beet, Ecb168 and NCPPB2795^T^, *P. betavasculorum* strain CFBP1520 isolated from sunflower, and *P. betavasculorum* strain SF142.2 isolated from the artichoke.

All tested *P. betavasculorum* strains demonstrate the same fatty acid composition but differ in their proportions. Strains isolated from sugar beets have more (56%–57%) unsaturated fatty acids (UFAs) represented mostly by monounsaturated fatty acid (MUFA) than saturated (SFA) (43%–44%). The opposite proportions were observed for strains isolated from sunflower and artichoke: 46% of unsaturated and 54% of saturated fatty acids (**Figure 3A**, **Table 3**). Polyunsaturated fatty acids (PUFAs) were more abundant in strains from sunflower and artichoke (**Figure 3D**, **Table 3**). Strains from sugar beets had significantly fewer branched fatty acids (BCFAs) than strains from sunflower and artichoke (**Figure 3C**, **Table 3**). In addition, the sugar beet strains had two to three times fewer even-chain fatty acids (ECFAs) while four to five times more odd-chain fatty acids (OCFA) than strains that came from other host plants (**Figure 3B**, **Table 3**). The differences in fatty acid composition observed between strains isolated from sugar beet and other plants were statistically significant (**Table 3**, **Supplementary Figure S10**).

### 3.3 Genotypic characterization

#### 3.3.1 Fingerprinting with ERIC-PCR

The genetic diversity of 14 P*. betavasculorum* strains originating from four different plant species and three different continents was assessed by the ERIC-PCR method and multi-locus sequence analysis.

**Figure 4:**
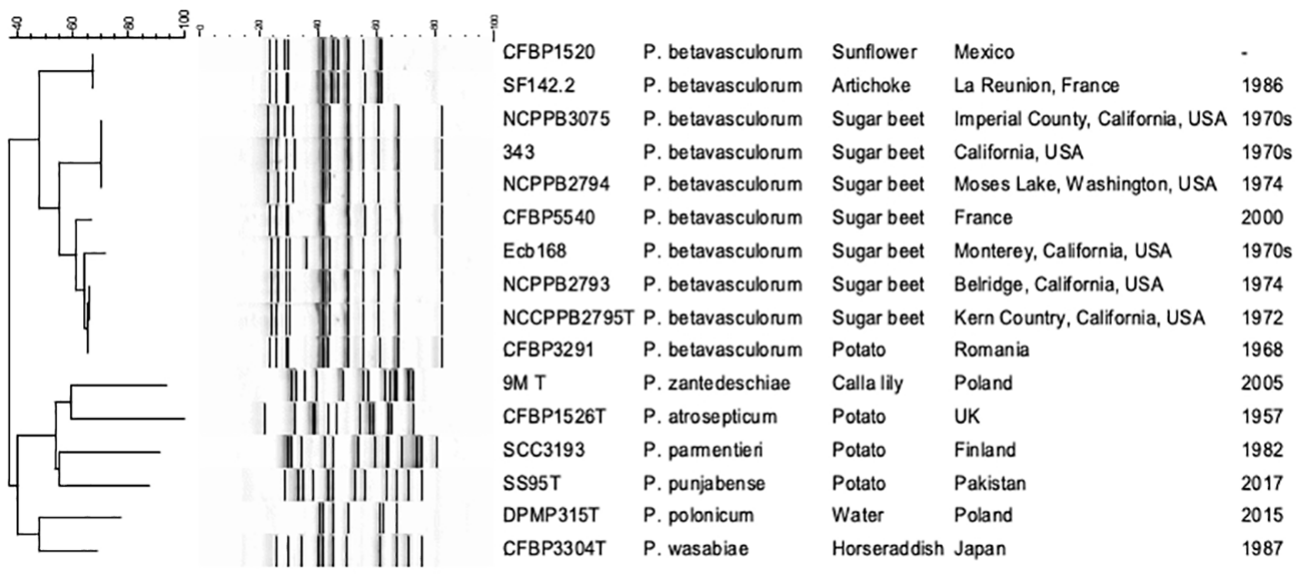
The electrophoretic patterns obtained after REP-PCR with ERIC primers. 1, *P. betavasculorum* Ecb168; 2, *P. betavasculorum* NCPPB2795^T^; 3, *P. betavasculorum* NCPPB2794; 4, *P. betavasculorum* 343; 5, *P. betavasculorum* 350; 6, *P. betavasculorum* NCPPB3075; 7, *P. betavasculorum* NCPPB2793; 8, *P. betavasculorum* 307; 9, *P. betavasculorum* CFBP5540; 12, *P. betavasculorum* CFBP3291; 13, *P. betavasculorum* CFBP1520; 14, *P. betavasculorum* SF142.2; 15, *P. zantedeschiae* 9M^T^; 16, *P. atrosepticum* ICMP1526^T^; 17, *P. parmentieri* SCC3193; 18, *P. wasabiae* CFBP 3304^T^; 19, *P. punjabense* SS95^T^; 20, *P. polonicum* DPMP315^T^. The neighbor joining cladogram was created with band base Jaccard coefficient.

Three ERIC profiles were discriminated against (**Table 1**, **Figure 4**). For strains CFBP1520 and SF142.2 isolated from sunflower and artichoke, the same fingerprint pattern was observed; however, it was distinct from those strains derived from sugar beet and potato. Furthermore, the sugar beet strains showed genetic diversity as two patterns were distinguished. Strain Ecb168 differed from the other strains isolated from this host plant.

#### 3.3.2 Multi-locus sequence analysis

Multi-locus sequence analysis (MLSA) analysis was performed for seven *P. betavasculorum* strains, namely, Ecb168, NCPPB2795^T^, NCPPB2794, CFBP3291, CFBP1520, SF142.2, and NCPPB3075, that were selected based on ERIC fingerprinting profiles. A total of 13 housekeeping genes, namely, *acnA*, *gapA*, *gyrA*, *gyrB*, *icdA*, *mdh*, *mtlD*, *pgi*, *proA*, *recA*, *recN*, *rpoA*, and *rpoS*, were merged. Next, the Maximum Likelihood phylogenetic analysis on concatenated gene sequences (20,770 bp) was performed to evaluate the evolutionary relationships among *P. betavasculorum* strains and other species within the *Pectobacterium* genus. The generated tree has shown the presence of two major clusters. The species *P. betavasculorum* belongs to the first of these and is most closely related to *P. zantedeschiae*, *P. atrosepticum*, and *P. peruviense*, while *P. polonicum*, *P. punjabense*, *P. parmentieri*, and *P. wasabiae* constitute their sister clade. All *P. betavasculorum* strains form two subgroups that form one clearly separated branch.

The second clade included all the other species of the genus *Pectobacterium* described so far except for *P. fontis* and *P. cacticidum*. These two species, in turn, are grouped separately (**Supplementary Figure S11**). Moreover, the ML tree showed that *P. betavasculorum* strains are separated into two groups. Strain CFBP3291 isolated from potato groups together with two sugar beet strains, NCPPB2793 and NCPPB2795^T^. At the same time, sugar beet strain Ecb168 is more related to strains CFBP150 and SF142.2 that originated from sunflower and artichoke, respectively.

#### 3.3.3 Phylogenomics

**Figure 5:**
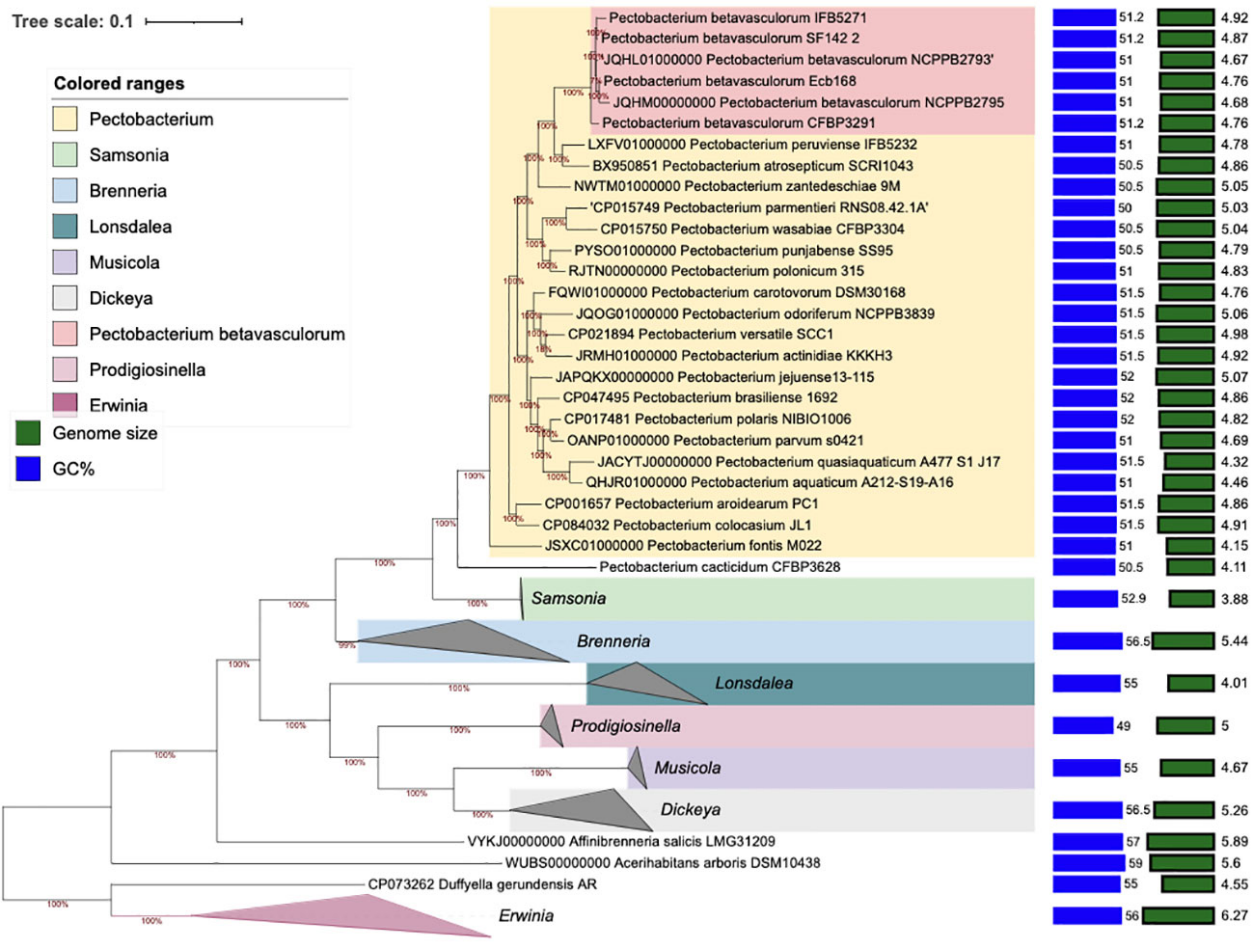
The phylogenomic analysis of *P. betavasculorum* strains based on the 400 most conserved universal proteins. The Maximum Likelihood tree was constructed using PhyloPhlAn computational pipeline (https://huttenhower.sph.harvard.edu/phylophlan, accessed on 7 August 2023).

The phylogenomic analysis based on core proteins showed similar results as MLSA and fingerprinting methods. All *P. betavasculorum* strains form one group (marked in coral pink) that is separated from other *Pectobacterium* species. *P. betavasculorum* is more closely related with species *P. peruviense*, *P. atrosepticum*, and *P. zantedeschiae* and more distantly with *P. polonicum*, *P. punjabense*, *P. parmentieri*, and *P. wasabiae* (**Figure 5**).

The ML tree for six genomes, built out of a core of 3,573 genes per genome (3,588,885 bp per genome) to establish a relationship between *P. betavasculorum* strains isolated from different plant species, was created. Strains CFBP150 and SF142.2 isolated from sunflower and artichoke are most similar to each other. Strain Ecb168 is most closely related to type strain NCPPB2795^T^, while strain CFBP3291 from potato is more related to strain NCBBP2793 from sugar beet (**Supplementary Figure S12A**). Additional genomic analyses, Percent of Conserved Proteins (POCP), Average Nucleotide Identity (ANI), and Average Amino acid Identity (AAI) were performed to resolve this dilemma also. According to POCP analysis, strains CFBP1520 and SF142.2 derived from sunflower and artichoke are most similar to each other and are a bit distant from strains from sugar beet NCPPB2793, NCPPB2795^T^, and Ecb168, and strain CFBP3291 from potato (**Supplementary Figure S12B**). In contrary to ANI, AAI analyses showed that strains CFBP150 and SF142.2 isolated from sunflower and artichoke are more similar to two strains isolated from sugar beet, Ecb168 and NCPPB2793, and all together are a bit distant from strains NCPPB2795^T^ from sugar beet and CFBP3291 derived from potato, which are most similar to each other (**Supplementary Figures S12C, D**).

#### 3.3.4 Genome sequencing and analysis

The genomes of four *P. betavasculorum* strains, CFBP1520 originated from sunflower in Mexico, SF142.2 isolated from artichoke on the French island La Reunion, CFBP3291 from potato grown in Romania, and Ecb168 from sugar beet in the USA, were sequenced, assembled, annotated, and deposited in GenBank under the following accession numbers CP118484, CP129215, JANIMZ000000000, and JARDVT000000000, respectively. The final genome sequences of strains Ecb168 and CFBP3291 are circulated and closed, while sequences of strains SF142.2 and CFBP1520 contain 48 or 63 scaffolds representing one chromosome each. The genome size ranged from 4.76 kBP to 4.93 kBP, and GC % was between 51.0% and 51.2% (**Table 2**).

Among the six *P. betavasculorum* isolates, only two strains, CFBP3291 and CBPPB2795^T^, harbor 23,763 bp long plasmid that is similar in 94.27% (100% coverage) with 21,742 bp long plasmid IncFII(pCRY) (NC005814) harboring a cryptic type of IVA secretory system (T4ASS) from *Yersinia pestis* biovar Microtus str. 91001 (Song et al., 2004). Plasmids pPB3291 (CP129216) and pPB2795 (JQHM00000026) from *P. betavasculorum* strains CFBP3291 and CBPPB2795^T^, respectively, are identical in 100%. Likewise, plasmid pCRY and both plasmids from *P. betavasculorum* strains contain 10 VirB proteins from T4SS (VirB1, TrbC/VirB2, ATPase VirB3, minor pilin of type IV secretion complex VirB5, VirB6, VirB8, VirB9, VirB10, and VirB11) and VirD, sequences for plasmid mobilization relaxosome protein MobB and MobC, RepA protein, chromosome partitioning protein ParA, sequences for dopa decarboxylase, conjugal transfer protein, Cag pathogenicity island protein Cag12, transcriptional antiterminator NusG, transcriptional regulator, EexN lipoprotein, conjugal transfer protein TrbL, and membrane protein. Moreover, the contig 4 corresponding to plasmid pPB3291 contains a thermonuclease, type II toxin-antitoxin system RelE/ParE, while scaffold 26, corresponding to plasmid pPB2795, contains additionally TriB protein ATP-binding protein, toxin-antitoxin system, toxin component HigB, micrococcal nuclease-like protein, and seven hypothetical proteins. The plasmids of *P. betavasculorum* strains are similar in 97.84% with plasmid pRHBSTW-00515_4 from *Klebsiella pneumoniae* strain RHBSTW-00515 (CP056428) isolated from livestock host (AbuOun et al., 2021, Microb Genom.) and in 94.8% (92% coverage) with numerous plasmids from clinical isolates of *Citrobacter freundii* (CP110909, CP110906, and CP110893).

The newly obtained genomic sequences were compared with genomes of two strains, NCPPB2793 and NCPPB2795^T^, isolated from sugar beet in the USA, which sequences are available in the GenBank under accession numbers JQHL00000000 and JQHM00000000, respectively. Genomes of strains isolated from sugar beet were slightly smaller (~4.7 kBP) than those of strains originating from other host plant species (~4.9 kBP). Consequently, this has resulted in a lower number of genes encoding proteins in the case of three strains isolated from sugar beet, NCPPB2793 (4,126), NCPPB2795^T^ (4,321), and Ecb168 (4,329), than for strains isolated from sunflower (4,581), artichoke (4,554), and potato (4,587). However, it should be noted that four out of six of the analyzed genomes are not closed. Summary information is presented in **Table 2**.

##### 3.3.4.1 Functional genome annotation

The RASTtk ModelSEED database was used to identify the metabolic pathways in *P. betavasculorum* using the Kyoto Encyclopedia of Gene and Genomes (KEGG). The subsystem category distribution of *P. betavasculorum* genomes annotated at RASTtk showed that approximately 30% of functioning features were present in ~350 subsystems (**Table 4**). Of these, the most abundant were features involved in amino acid and derivative subsystems 269–298 (14.7%) and metabolism of carbohydrate 308–358 (14.8%), protein 200–230 (12%), cofactors, vitamins, prosthetic group, and pigments 155–167 (8%), and features involved in membrane transport 123–148 (7.5%).

**Table 4:** The subsystem category distribution in *P. betavasculorum* genomes.

Subsystem Category Distribution	No of subsystem feature counts in *P. betavasculorum*					
	CFBP1520	SF142.2	CFBP3291	Ecb168	NCPPB2793	NCPPB2795
Total number of subsystems	342	353	356	344	349	354
Cofactors, vitamins, prosthetic groups, pigments	158	157	157	155	163	159
Cell wall and capsule	36	35	36	36	36	35
Virulence, disease, and defense	40	38	36	37	42	38
Potassium metabolism	8	8	8	8	8	8
Photosynthesis	0	0	0	0	0	0
Miscellaneous	15	15	15	15	15	15
Phages, prophages, transposable elements, plasmids	10	9	12	6	2	11
Membrane transport	142	142	133	123	141	148
Iron acquisition and metabolism	29	28	28	28	28	28
RNA metabolism	52	52	52	52	50	52
Nucleosides and nucleotides	97	97	95	95	94	95
Protein metabolism	230	203	212	211	200	202
Cell division and cell cycle	7	7	7	7	7	7
Motility and chemotaxis	17	16	16	16	16	18
Regulation and cell signaling	58	57	59	56	61	58
Secondary metabolism	4	4	4	4	4	4
DNA metabolism	83	82	84	77	82	84
Fatty acids, lipids, and isoprenoids	54	53	51	51	55	54
Nitrogen metabolism	**58**	**55**	40	40	40	39
Dormancy and sporulation	3	3	3	1	3	3
Respiration	93	93	92	92	92	92
Stress response	72	71	71	71	73	71
Metabolism of aromatic compounds	4	4	2	2	2	2
Amino acids and derivatives	311	307	305	310	309	308
Sulfur metabolism	23	23	24	24	23	24
Phosphorus metabolism	25	25	25	25	25	25
Carbohydrates	269	270	275	271	272	270

The significantly higher number of subsystems categorised as nitrogen metabolism detected in *P. betavasculorum* strains isolated from low-sugar tissue plants than in strains derived from high-sugar sugar beet and potato tubers was marked in bold.

We observed differences between *P. betavasculorum* strains in the number of genes involved in nitrogen and aromatic compounds metabolism. Strains isolated from sugar beet and potato do not have *nif* operon encoding genes responsible for atmospheric nitrogen fixation and genes responsible for salicylate and gentisate catabolism that are present in two strains originated from sunflower and artichoke (**Table 4**).

Furthermore, to determine differences in functions of each gene of *P. betavasculorum* genome, we analyzed our data with KEGG and COG; 7157/038 functional categories (including not annotated sequences) with *P. betavasculorum* strain Ecb168 as a reference were found in the selected contigs, distributed in core dispensable and singleton genes. Analyzing all annotated KEGG/COG genes, 0.33%/0.28% have inconsistent category information.

**Figure 6:**
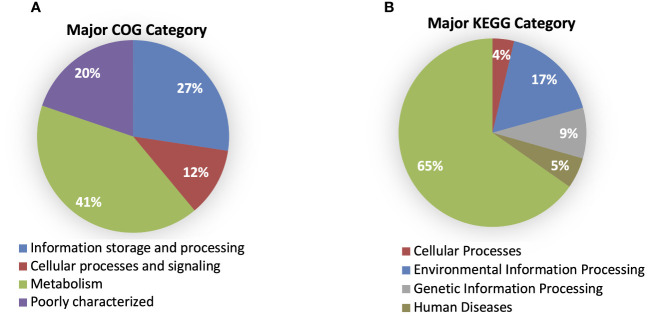
The % of genes annotated to major **(A)** COG and **(B)** KEGG categories for *P. betavasculorum* genomes.

According to the cluster of orthologous genes (COG) database, 86.5% of genes were annotated, and 41% of them were involved in metabolism processes, 27% were engaged in information processing and storage, 12% in cellular processing and signaling, while 20% of genes belonged to the poorly characterized category. In the cases of all *P. betavasculorum* genomes, the two s subgroups among the metabolism category contain genes annotated to the metabolism and transport of amino acids and carbohydrates; they contain almost 9% and 8% of genes, respectively (**Figure 6**, **Supplementary Table S3**).

**Figure 7:**
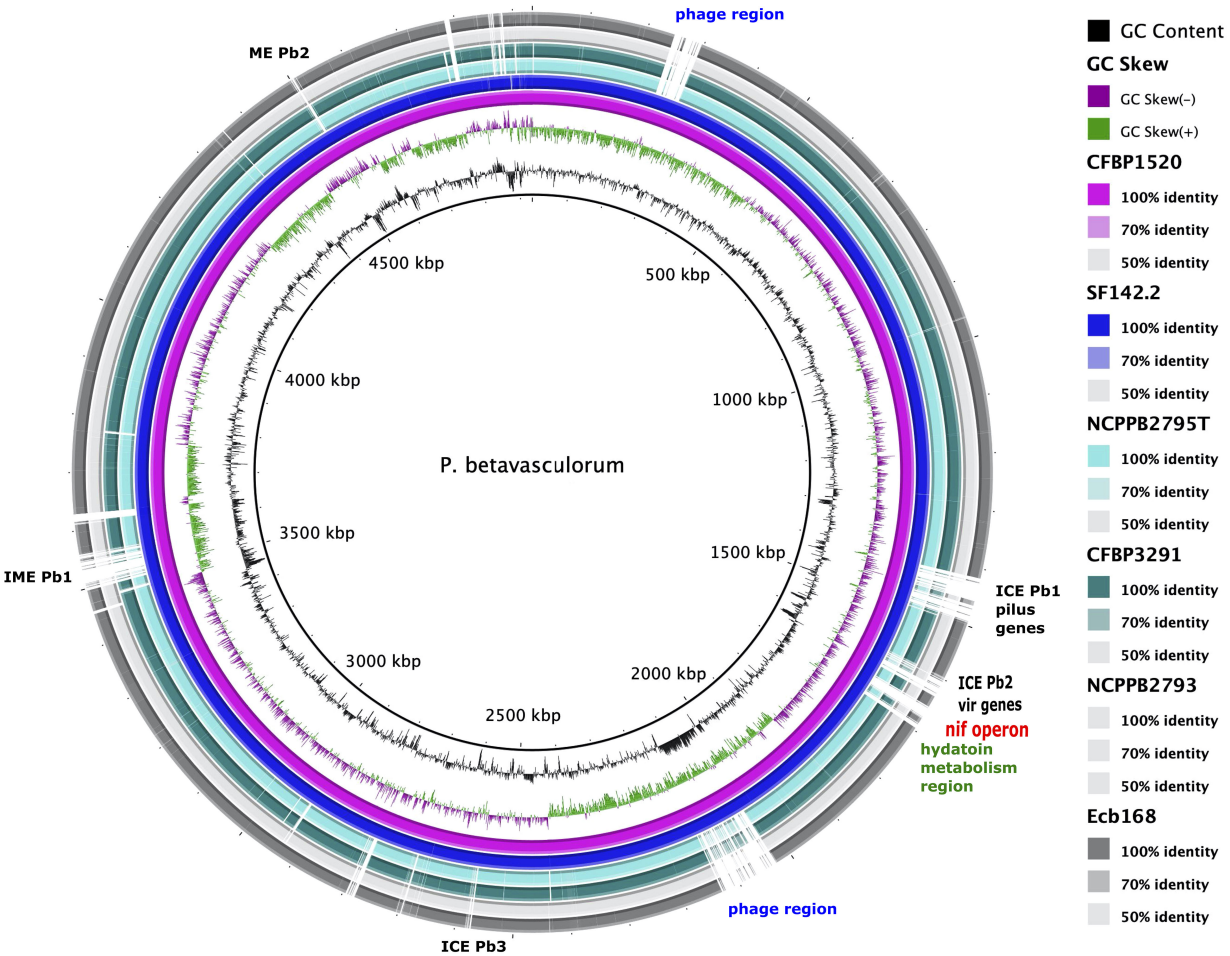
A graphical comparison of the whole genome sequences of *P. betavasculorum* strains: CFBP3291, Ecb168; NCPPB2795^T^, CFBP1520, and SF142.2. The circular map was created using BLAST Ring Image Generator software (Alikhan et al., 2011). The genome of strain *P. betavasculorum* CFBP3628 type strain was used as a reference. First rings correspond to the GC content and GC skew, respectively. Each of the depicted rings refers to one *P. betavasculorum* genome according to the listed coloration. White regions mark dissimilarities.

Using the KEGG database, 73% of genes have been annotated; among them, 65% of genes were annotated to metabolic processes, 17% to environmental information processing, 9% to genetic information processing, 5% were classified to human diseases category, and 4% of genes were connected to cellular processes. In all of the six analyzed genomes of *P. betavasculorum*, the same number of genes classified into each KEGG category was observed. Two categories, the signaling and cellular processes and genetic information processing, were the most prominent groups and contained 16% (916–929) and 11% of genes (612–640), respectively. The third largest group was a category of genes whose function was related to processing environmental information (450–462 genes, 8%). Approximately 4.5% of the genes each fell into the category of cellular processes and metabolism of carbohydrates and proteins (**Figure 6**, **Supplementary Table S3**). A visual comparison of genome homology done using BRIG (BLAST Ring Image Generator) showed that most regions within the analyzed genomes were conserved between genomes of *P. betavasculorum* strains. However, there were apparent differences between them (**Figure 7**).

#### 3.3.5 Pangenomics

**Figure 8:**
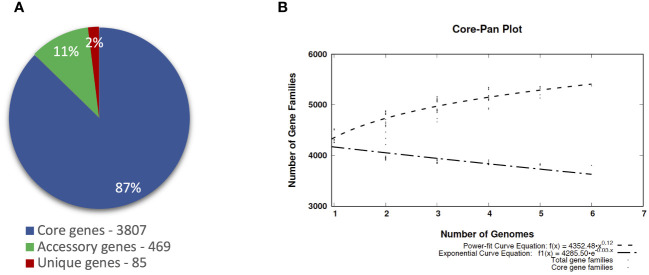
The pangenome profile of *P. betavasculorum* species calculated with BPGA software Chaudhari et al., 2016). **(A)** Abundancy of the core, accessory, and unique pangenome fractions within the pangenome of *P. betavasculorum.* **(B)** The number of distinct gene families referring to the pangenome size (dashed line; power-fit curve equation: f(x) = 4,224.574 · x^0.14^) in addition to the number of core gene families (dash-dotted line; exponential curve equation: f1(x) = 4272.24 · e^−0.004x^) are plotted against the number of genomes included.

The pangenomic analysis revealed that the pangenome of *P. betavasculorum* is open and comprises, on average, 4,361 gene families. Of these, 87% of genes are the core genome, and the unique genes are only 2% of the entire pangenome of this species (**Figure 8**).

The largest number of genes, 4,525, was recorded for the strain CFBP1520 from sunflower and the smallest, 4,265, for the strain NCPPB2793 isolated from sugar beet. Interestingly, the number of genes of strains CFBP1520 and SF142.2 originating from sunflower and artichoke, respectively, were bigger than those observed for strains NCPPB2793, NCPPB2795^T^ Ecb168, and CFBP3291 isolated from sugar beet and potato (**Table 5**). The complete genomes of these bacteria share 3,807 gene families, which represent over 80% of CDS in each genome. The highest unique CDS number was observed in the case of two strains isolated from sugar beet Ecb168 and NCPPB2793 (239 and 177, respectively), which was at least 10 times more than the unique CDS detected in genomes of four strains. In the case of strains SF142.2 NCPPB2795^T^ and CFBP3291, all unique CDS are annotated as hypothetical proteins. For the strain Ecb168, there were 239 unique CDS. The majority of them, 181 (70%), are coding hypothetical proteins. The CDS that are unique for this strain are genes encoding phage-related proteins, and three different type II toxin-antitoxin systems, RelB/DinJ, YafO/YafM, and HicA/B, VirB family type IV secretion system proteins. Among 177 CDS unique for strain NCPPB2793, 65 encoded hypothetical proteins, six CDS of membrane proteins, three genes for integrases, three encoding flavodoxin, one gene encoding flavodoxin/nitric oxide synthase, and two genes for aldehyde oxidase, transposases, DNA primases, transcriptional regulators, cold-shock proteins, non-ribosomal peptide synthetase module, LysR family transcriptional regulators, and type IV secretion protein Rhs, and 83 single genes listed in **Supplementary Table S4**.

**Table 5:** Pangenomic analysis of *P. betavasculorum* undertaken with BPGA software.

Strain	Host plant	Total No. of genes	No. of core genes	No. of accessory genes	No. of unique genes	No. of exclusively absent genes
CFBP1520	sunflower	4,525	3,807	695	23	8
SF142.2	artichoke	4,507	3,807	687	13	4
CFBP3291	potato	4,287	3,807	433	14	7
Ecb168	sugar beet	4,317	3,807	271	239	30
NCPPB2793	sugar beet	4,265	3,807	281	177	18
NCPPB2795^T^	sugar beet	4,303	3,807	482	14	7

The independent pangenomic analysis was performed with the implementation of the GET_HOMOLOGUES software package (Contreras-Moreira and Vinuesa, 2013), which allows the calculation of core genome with the application of three different methods (COG, BBDH, and OrthoMCL) and the discrimination of intersections between strains of different origins. (**Supplementary Figure S13**).

The genomes of three *P. betavasculorum* strains, Ecb168, NCPPB2793, and NCPPB2795^T^ isolated from sugar beet, shared only one common and unique gene that was not present in the genomes of strains isolated from other vegetables. Unfortunately, the function of this protein is not known.

Among five genes that were unique for three strains CFBP3291, CFBP1520, and SF142.2 originated from plants other than sugar beet, there were two hypothetical proteins, AlpA family transcriptional regulator, phage-related DUF4222 domain-containing protein and zinc-ribbon domain-containing protein.

Interestingly, genomes of two strains, CFBP1520 and SF142.2, isolated from plants whose tissues do not contain high sugar concentration (artichoke and sunflower) share 391 common CDS that are not present in other strains from sugar beet or potato. Many of them, 138 (35%), are hypothetical proteins. Interestingly, only these two strains harbored genes *nifA*, *nifB*, *nifD*, *knife*, *nifH*, *nifL*, *nifM*, *nifN*, *nifQ*, *nifT*, *nifU*, *nifW*, *nifX*, and *nifZ* that formed *nif* operon and are involved in nitrogen fixation (**Figure 7**, **Supplementary Figure S14**). Moreover, they shared two different type II TA systems, YdaS/YdaT and *yafN/yafO*, type IV TA system AbiEJ/AbiG, three restriction endonucleases including Eco47II-like restriction-modification (R-M) system type II, genes related to prophages, seven integrases, proteins Ync and Ynd being a part of conjugal type IV macromolecular transfer systems secretion system, outer membrane ferripyoverdine receptor from Ton and Tol transport systems, DNA-cytosine methyltransferase (EC 2.1.1.37), and two enzymes responsible for salicylate catabolism, salicylate esterase (SalE), and fumarylacetoacetate hydrolase (Fhf), and transporters and transcription regulators (**Supplementary Table S4**).

Four strains, CFBP3291, Ecb168, NCPPB2793, and NCPPB2795^T^, derived from plants with high sugar content (potato and sugar beet) share only 31 unique CDS. Among them are genetic determinants of enzymes responsible for histidine degradation [forminoglutamic iminohydrolase (EC 3.5.3.13), histidine ammonia-lyase (EC 4.3.1.3), histidine utilization repressor, imidazolonepropionase (EC 3.5.2.7), N-formylglutamate deformylase (EC 3.5.1.68), urocanate hydratase (EC 4.2.1.49)] (**Supplementary Figure S15**), ABC transporters (substrate-binding protein and permease-cluster 13, osmolytes), stresses-induced protein Ves (HutD), formiminoglutamic iminohydrolase (EC 3.5.3.13), and genes for phage-related proteins and 10 hypothetical proteins with unknown functions.

Moreover, comparative analysis allows us to identify 162 genes that are unique for two strains CFBP3291 and NCPPB2795^T^ containing plasmid. Among them, 71 are coding hypothetical proteins and genes of phage-related proteins, genes *mcrBC* and *mrr* encoding type IV RM system, and genes *hsdM*, *hsdR*, and *hsdS* coding complete RM system type I. They shared two different type II TA systems, *yafN/yafO* and *relE/rarE*, and the solitary gene of *yeeU* encoding antitoxin to YeeV, and the genes of flagellin lysine methyltransferase FliB, cag pathogenicity island Cag12 family protein, and aromatic l-amino acid decarboxylase. On the contrary, four strains without plasmid have only 32 unique genes mainly encoding hypothetical proteins, phage-related proteins, integrases, relaxase, and transcriptional regulators (**Supplementary Table S4**).

**Figure 9:**
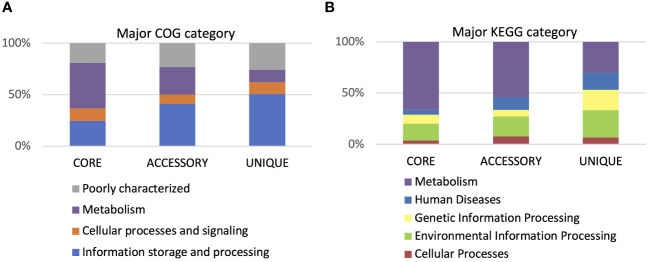
Gene annotation by COG and KEGG categories. **(A)** Major COG category and **(B)** major KEGG category.

**Figure 10:**
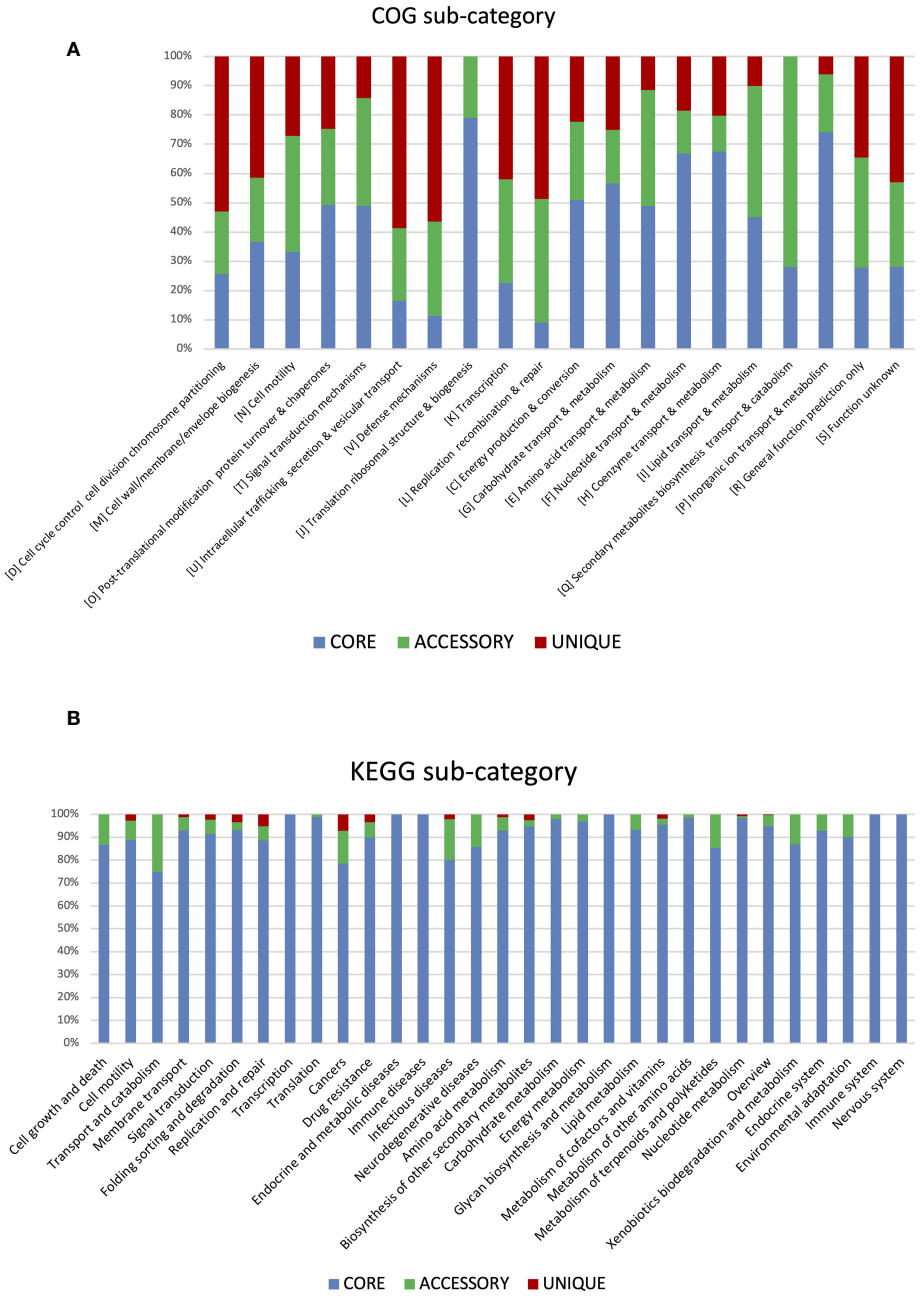
Gene annotation by COG and KEGG sub-categories. **(A)** Major COG sub-category and **(B)** major KEGG sub-category.

Functional annotation of genes within the core genome, accessory, and unique genes according to both COG and KEGG databases showed that most functions focused on metabolic processes (**Figure 9**). However, twice as few genes determining metabolic processes belong to the accessory part of the genome and four times fewer among unique genes. The opposite trend can be observed in the case of genes related to environmental information processing and information storage processing. In the core genome, the four COGs categories are the most numerous: [J] Translation ribosomal structure and biogenesis, [P] Inorganic ion transport and metabolism, [H] Coenzyme transport and metabolism, and [F] Nucleotide transport and metabolism. In the accessory part of the genome, the most abundant is the [Q] Secondary metabolites biosynthesis transport and catabolism category, while among the unique genes, the largest are two categories, [U] Intracellular trafficking secretion and vesicular transport and [V] Defense mechanisms (**Figure 10**). Using KEGG database, over 70% of genes from each category belonged to the core genome. The same KEGG sub-category related to carbohydrate metabolism is the largest in core, accessory, and unique part of *P. betavasculorum* genomes. The second largest subcategory in the core genome gathers genes related to membrane transport; in the accessory part of the genome, it was the amino acid metabolism subcategory, while within the unique genes, replication and repair category (**Supplementary Table S3**).

#### 3.3.6 Comparative genomics

Comparative genomics analyses were also performed to further identify distinctive traits between the *P. betavasculorum* and other *Pectobacterium* species for which genomes are available in the GenBank. Using the function-based comparison tool under RAST [29], 59 genes were predicted to be specific for *P. betavasculorum* (**Table 6**, **Supplementary Table S2**). Among them, we found 25 genes encoding hypothetical proteins, three genes encoding ABC transporters, two genes of transposases of insertion sequence (IS), two genes encoding major facilitator family (MSF) transporters, four encoding transcriptional regulators from LysR and GntR families, efflux transporter periplasmic adaptor subunit (*hlyD*), hemagglutinin repeat family protein, thiol: disulfide intercharge protein, or two phage-related proteins. Moreover, unique CDs encode proteins involved in amino acid synthesis and transport. Interestingly, *P. betavasculorum* is the only species of the genus *Pectobacterium* with genes encoding isopentyl transferase, allantoin permease, urea carboxylase and allophanate hydrolase gene cluster, genetic determinants of acetoin and butanediol biosynthesis, or genes responsible for sulfoquinovose metabolism (**Supplementary Figure S16**).

**Table 6:** List of CDs unique for *P. betavascuolorum*.

Locus	Most similar protein	Species	Protein id	Identity
JV35_02540	T3SS effector HopA1 family protein	*Dickeya fangzhongdai*	WP_039691973	74.00%
JV35_03105	Inulin fructotransferase DFA-I-forming	*Raoultella terrigena*	VTM17954	93.60%
JV35_03115	LacI family DNA-binding transcriptional regulator	*Dickeya fangzhongdai*	WP_161129920	85.70%
JV35_03350	MFS transporter	Candidatus Sodalis endolongispinus	WP_215671307	81.60%
JV35_06670	allantoin permease	*Brenneria* sp. hezel4-2-4	WP_172289733	82.40%
JV35_06675	biotin-dependent carboxyltransferase family protein	*Brenneria roseae*	WP_109052927	82.20%
JV35_06680	allophanate hydrolase subunit 1	*Brenneria* sp. L3-3C-1	WP_199377503	85.30%
JV35_06685	acetyl-CoA carboxylase biotin carboxylase subunit	*Brenneria izadpanahii*	WP_208227955	81.80%
JV35_06690	acetyl-CoA carboxylase	unclassified *Brenneria*	WP_172289737	87.70%
JV35_06700	LysR family transcriptional regulator	unclassified *Brenneria*	WP_264089307	92.10%
JV35_07930	multidrug effflux MFS transporter	*Dickeya dadantii*	WP_226056120	84.50%
JV35_07935	hypothetical protein	*Superficieibacter* sp. 1612_C1	WP_194205386	83.50%
JV35_07940	FAD-NAD(P)-binding protein	*Siccibacter turicensis*	WP_130098341	80.40%
JV35_07980	Bcr/CflA family drug resistance efflux transporter	*Pseudomonas* sp.	HBK47856	91.50%
JV35_08490	radical SAM protein	*Pantoea rwandensis*	WP_038649575	92.90%
JV35_08500	MFS transporter	*Pantoea rwandensis*	WP_038649572	81.90%
JV35_10605	transposase InsO family protein	*Samsonia erythrinae*	TCV01277	79.30%
JV35_11300	HAD family hydrolase	*unclassified Brenneria*	WP_172291444	66.00%
JV35_11305	hypothetical protein	*Brenneria* sp. hezel4-2-4	WP_172291445	68.10%
JV35_11310	adenine phosphoribosyltransferase	unclassified *Brenneria*	WP_172291446	78.50%
JV35_11315	4a-hydroxytetrahydrobiopterin dehydratase	unclassified *Brenneria*	WP_172291447	55.00%
JV35_11670	nickel ABC transporter substrate-binding protein	*Brenneria roseae*	WP_245929510	90.80%
JV35_11675	nickel ABC transporter permease subunit NikB	*Samsonia erythrinae*	WP_132453974	93.50%
JV35_11680	ABC transporter permease subunit	*Brenneria rubrifaciens*	WP_137712457	92.00%
JV35_11690	dipeptide/oligopeptide/nickel ABC transporter ATP-binding protein	*Samsonia erythrinae*	WP_132453980	79.10%
JV35_11905	helix-turn-helix transcriptional regulator	*Brenneria roseae*	WP_136157317	86.60%
JV35_11975	acetoin reductase	*Gibbsiella quercinecans*	WP_095844696	88.00%
JV35_11980	lipoate–protein ligase family protein	*Gibbsiella quercinecans*	WP_121554382	84.60%
JV35_11985	acetoin dehydrogenase dihydrolipoyllysine-residue acetyltransferase subunit	*Gibbsiella quercinecans*	WP_095844694	82.80%
JV35_11990	alpha-ketoacid dehydrogenase subunit beta	*Serratia* sp. ATCC 39006	WP_021014310	97.60%
JV35_11995	thiamine pyrophosphate-dependent dehydrogenase E1 component subunit alpha	*Gibbsiella quercinecans*	WP_230469530	96.10%
JV35_12005	sigma-54-dependent Fis family transcriptional regulator	*Serratia* sp. ATCC 39006	WP_021014307	83.40%
JV35_14355	SLC13 family permease	*Brenneria* sp. hezel4-2-4	WP_253906971	94.61%
JV35_14410	response regulator transcription factor	*Brenneria salicis*	WP_113864949	73.90%
JV35_14415	ATP-binding protein	unclassified *Brenneria*	WP_264089569	70.90%
JV35_14420	TonB-dependent receptor	unclassified *Brenneria*	WP_264089570	86.70%
JV35_15785	isopentenyl transferase	unclassified *Brenneria*	WP_172291217	63.00%
JV35_15790	hypothetical protein	unclassified *Brenneria*	WP_172291218	57.60%
JV35_16070	MFS transporter	*Citrobacter werkmanii*	EGT0663977	94.30%
JV35_16075	MFS transporter	*Klebsiella*	WP_154931221	97.40%
JV35_16080	alpha-glucosidase	*Citrobacter braakii*	WP_200103035	89.40%
JV35_16085	sulfoquinovose isomerase	*Citrobacter farmeri*	WP_125370327	95.40%
JV35_16090	sulfofructosephosphate aldolase	*Citrobacter freundii*	WP_181509827	94.80%
JV35_16095	sulfolactaldehyde 3-reductase	*Citrobacter werkmanii*	WP_200009470	94.90%
JV35_16100	sugar kinase	*Citrobacter* sp. Marseille-Q3906	WP_225760329	90.30%
JV35_16105	DeoR/GlpR family DNA-binding transcription regulator	*Citrobacter cronae*	WP_262756140	98.50%
JV35_16365	glycerol-3-phosphate transporter	*Selenomonas* sp. oral taxon 126	WP_066845247	70.00%
JV35_16675	hybrid non-ribosomal peptide synthetase/type I polyketide synthase	*Corallococcus coralloides*	WP_014395406	46.70%
JV35_16680	chlorinating enzyme	*Pseudoduganella buxea*	WP_155473017	69.60%
JV35_16690	Peptide synthetase and polyketide synthase	*Mycobacteroides abscessus* subsp. *abscessus*	SIG24094	33.20%
JV35_16695	alpha/beta fold hydrolase	*Corallococcus coralloides*	WP_128796085	34.60%
JV35_16700	MATE family efflux transporter	*Pseudoduganella buxea*	WP_155473013	50.20%
JV35_16715	LysE family transporter	*Photorhabdus luminescens*	WP_105397716	49.50%
JV35_18930	amino acid adenylation domain-containing protein	*Brenneria tiliae*	WP_249243655	80.50%
JV35_19240	hypothetical protein AE12_03583	*Escherichia coli* UCI 53	KDG74815	89.70%
JV35_19245	LuxR C-terminal-related transcriptional regulator	*Erwinia piriflorinigrans*	WP_167337179	45.90%
JV35_19515	acyl-CoA dehydrogenase family protein. partial	*Acidovorax avenae*	WP_107178314	64.30%
JV35_19830	CidA/LrgA family protein	*Samsonia erythrinae*	WP_132456095	89.10%
JV35_19835	CidB/LrgB family autolysis modulator	*Samsonia erythrinae*	WP_132456097	93.51%

#### 3.3.7 Pathogenomics

The genomic comparison of *P. betavasculorum* strains revealed the presence of 106 genes responsible for the synthesis of various virulence factors like plant cell wall-degrading enzymes (51) and toxins (25), and 106 genes encoding proteins budling six secretion systems (T1SS–T6SS) and outer membrane vesicles (known as T0SS) (**Table 7**).

**Table 7:** List of genes encoding virulence factors and virulence regulators detected in the in *P. betavasculorum* genomes.

Type	Product	Gene No.	Gene
PCWDEs	Pectate lyases	10	*pelA*. *pelB*. *peC*. *pelI*. *pelL*. *pelW*. *pelX*. *pelZ*. *hrpW*, *pelK*.
	Pectinases	1	*pnl*
	Pectin acetylesterases	2	*paeX. paeY*
	Pectin methylesterases	2	*pemA. pemB*
	Polygalacturonases	4	*pehA. pehK. pehN. pehX*
	Cellulases	3	*celB. celV. bcsZ*
	Alpha glucosidases	5	*aglB (PYV01_05880), aglB2 (PYV01_12555), PYV01_12540, PYV01_20850, PYV01_12550*
	Beta glucosidases	7	*bglA, bglB, bglF, bglX, nagZ, ascB, celH*
	Oligo-galacturonide lyase	1	*ogl*
	Rhamno-galacturonate lyase	1	*rhiE*
	Proteases	15	*prtW, prt1, lasA*, N-acetyl-L,L-diaminopimelate deacetylase, peptidase M20, peptidase M15*, peptidase M3, protease M48, peptidase S58, dppA*, tripeptide aminopeptidase, N-acyl-L-amino acid amidohydrolase, extracellular serine protease precursor, peptidase C39, hydrolase
Secretion systems	T1SS	12	*lapB, lapC, lapE, lapD, lapP, RTX* *lapG*. *aggA (tolC)*. *prtD*. *prtE*. *prtF*. *lapB*. *lapC*. *lapD*. *lassD*. *mdsABC. pdeH*
	T2SS	17	*outS*. *gspB*. gene encoding pectate lyase precursor. *pehX. gspC. gspD. gspE. gspF gspG. gspH. gspI. gspJ. gspK. gspL. gspM. gspN. gspO.*
	Flp/Tad	15	*tadG. KP22_09205. tadE. tadD. tadC*. *tadB*. *tadA. tadZ. rcpB. rcpA. rcpC. tadV. flp* KP22_09270. sensor histidine kinase (KP22_09265)
	T4a-pilus	*10*	*pilW, fimT, pilC, pilB, pilA, pilM, pilN, pilQ, pilT, gspO*
	T3SS	32	transcriptional regulator*. dspF, dspE, hrpW, hrpW*-chaperon, *hrpN, hrpV, hrpT, hrcC, hrpG, hrpF, hrpE, hrpD, hrcJ, hrpB, hrpA*, phosphodiesterase A (KP22_05650), *hrpS. hrpY, hrpX, hrpL, hrpJ, hrcV, hrpQ, hrcN, hrpO, hrpP, hrcQ, hrcR, hrcS, hrcT, hcrU*
	T4SS	10	virB11*, virB10*, virB9*, virB8*, virB6*, virD4*, virB5*, virB4*, virB2*, virB1*
	T5SS	3	*hecB, hlyC, hecA*
	T6SS	17	*impB*, *impC*, *impF*, *iraD*, *vasA*, *vasB*, *vasC*, *vasD*, *vase*, *vasF*, *vasG*, *vasH*, *vasI*, *vasJ*, *vasK*, *vasL*, KP22_16860.
	TA systems	25	*higB21*, *relE*, *rhaS*, *symE*, *cvpA*, *prtC*, *y4kP*, *higB22*, *hlyC*, *rtxC*, *ortT*, *cbtA*, *abiEii*, *parE1*, *ccdB*, *aebG*, *past*, *cptA*, *ccdB*, *yoeB*, *parE3*, *higB23*, *tabA*, *pinD*, *stbE*
Phytotoxins	Coronofacic acid	10	*cfa1, cfa2, cfa3, cfa4, cfa5, cfa6, cfa7, cfa8A, cfa8B, cfa3, cfl*
Bacteriocins	carotovoricin	19	*ctv* genes: KP22_06505, KP22_06510, KP22_06515, KP22_06525, KP22_06530, KP22_06535, KP22_06540, KP22_06545, KP22_06555, KP22_13600, KP22_06565, KP22_06570, KP22_06575, KP22_06580, KP22_02520, KP22_02515, KP22_02510, KP22_02505, KP22_02500
	carbapenem	9	*carR (luxR), carA, carB, carC, carD (cpmD), carE, carF (cpmF, cpmI), carG (cpmG, cpmJ), carH (cpmH, cpmK)*
	carocin	2	caroDK^*^, caroDI ^*^
	phenazine	12	*ehpA, ehpB, ehpC, ehpD, ehpE, ehpF, ehpG, ehpR*, KP22_RS02880 (Polyketide synthase modules and related proteins), KP22_02935 (putative malonyl CoA-acyl carrier protein transacylase), KP22_RS02850 (hypothetical protein), KP22_02890 (phenazine antibiotic biosynthesis protein)
Iron uptake systems	Achromobactin uptake cluster	5	*cbrD, cbrC, cbrB, cbrA, acr*
	Enterobactin synthesis cluster	8	*entD, cirA (cir, feuA)*, KP22_19820 (putative iron transporter), *entC, entE, entB, entF, entA*
Acetoin and 3-Hydroxy-2-Butanone (3H2B) pathway gene cluster		9	KP22_09560 (Transcriptional regulator LysR), KP22_09540_ putative membrane protein*, budR, budA, budB*, KP22_09520 alpha/beta hydrolase, *sspA, sspB, budC, gltD*
S28. Exopolysaccharide (EPS) and O-antigen genes		10	KP22_01465 (Undecaprenyl-phosphate alpha-N-acetylglucosaminyl 1-phosphate), *wza, wzb, wzc/epsC, rfbN, rfbD, rfbC, rfbA, galF, gnd*
Lipo-oligo/polysaccharide (LOS/LPS)		9	*rfaD, waaF, waaC, waaL1, waaQ, waaG, waaI*, *waaA (kdtA), coaD (ktdB)*
Enterobacterial Common Antigen (ECA) genes		11	*wecG, wzyE, wecF, wzxE, rffA, rffC, rffG, wecC, wecB, wzzE, wecA*
Other virulence genes		5	*svx/avrXca, nip/nep1, cit1, appA, rplY*
Virulence regulator genes		23	*expI, expR1, virR (expR2), virS (acrR), rscR, luxS, expA (gacA, uvrY, sirA), expS (gacS, rpfA, barA), pehR (phoP), pehS (phoQ), rcsC, rcsB, rcsD, rsmA (csrA), kdgR, expM, hor (slyA), hexA (lrhA), hexY (rsmC), evr, fur, sirB1, cytR*

^*^Genes present only in strains CFBP3291 and NCPPB2795^T^ that possess plasmids.

##### 3.3.7.1 PCWDEs

A total of 40 known or putatively related genes encoding pectinases, cellulases, and proteinases were identified (**Table 7**). The genomes of *P. betavasculorum*, like other *Pectobacterium* species, contain 10 genes: *pelA*, *pelB*, *pelC*, *pelZ*, *pelI*, *pelK*, encoding pectate lyases, *pelW—*pectate disaccharide lyase, including *pelX—*exopolygalacturonate lyase, *hrpW* that encodes type III effector HrpW, hairpin with pectate lyase domain, and pectate lyase L precursor *pelL*. However, strains of *P. betavasculorum*, as the only species of the genus *Pectobacterium*, lack the *pelY* gene encoding a periplasmic pectate lyase. In addition, the following genes were detected: *pnl* for a pectin lyase; *pemA* and *pemB* for pectinesterase; *paeX* and *paeY* for pectin acetylesterase; *pehX*, *pehN*, *pehA*, and *pehK* for polygalacturonases; *ogl* for an oligogalacturonide lyase; and *rhiE* for a rhamnogalacturonate lyase.

Three genes in *P. betavasculorum* are involved in cellulose degradation, including glycoside hydrolase *celB(celS)*, and two endoglucanase-encoding genes, *celV* and *bcsZ*. Moreover, five beta-glucosidase-encoding genes *bglA*, *bglB*, *bglD*, *nagZ*, and *celH*, and five an alpha-glucosidase-encoding genes were detected. An operon of nine genes encoding cellulose synthetase, including *bcsC*, *bcsA*, *bcsB*, *bcsG*, *bcsE*, *bcsZ*, *bcsQ*, *bcsF*, and *bcsR*, was also identified in all of six analyzed *P. betavasculorum* genomes. Moreover, 21 genes encoding proteases or peptidases were detected.

##### 3.3.7.2 Secretion systems

Six types of secretion systems (TSS) were detected in the genomes of analyzed six *P. betavasculorum* strains. In total, 12 genes were involved in the secretion system Type I (T1SS), 17 in Type II (T2SS), 32 in Type III (T3SS), 10 in Type IV (T4SS), three in Type V (T5SS), and 17 in Type VI (T6SS) secretion systems (**Table 7**).

*P. betavasculorum* strains and other *Pectobacterium* species possess genes related to T1SS, *prtDEF*, *lssBEF*, *lapBCDEG*, *aggA*, and *pdeH*, while genes encoding the *hasDEF* are missing in their genomes. The complete T2SS Out secretion system and the twin-arginine translocation (Tat) protein system are present in the analyzed genomes of *P. betavasculorum* strains. The T3SS gene cluster in *P. betavasculorum* strains consists of hypersensitive response and pathogenicity conserved genes (*hcr*)/hypersensitive response and pathogenicity or hairpin proteins (*hrp*), genes encoding DspE (disease-specific effector protein E), DspF (disease-specific chaperone protein F), HrpW- and HrpK-T3SS effector proteins, and chaperon proteins. Interestingly, the type IV secretion system consisting of 12 *vir* genes was detected only in the case of two strains CFBP3291 and NCPPB2795^T^, as it was located on the plasmid that is harbored by these strains. The complete set of genes (*pilABCDTQMNOPVW)* encoding type IV pilus machinery was present in all of *P. betavasculorum* genomes. The type Vb secretion system (T5SS) consisting of two genes, *hecB* and *hecA*, encoding hemolysin activation protein and hemolysin/hemagglutinin-like protein, were found in *P. betavasculorum* genomes. In the case of the T6SS, only the core part of this injectosome, delivering toxic effector proteins into bacterial or host cells, was present. The 17 genes encode the outer membrane lipoprotein (VasD), inner membrane proteins (ImpL/VasK and ImpK/VasF), ATPase (ClpV), and regulatory proteins or structure proteins (ImpB, ImpC, TssE, ImpG, ImpH, ImpI, ImpJ, VasH, VasI, VasJ, and VasL). While the gene encoding hypothetical proteinKP22_16860, gene *vgrG* (valine–glycine repeat protein G), and *hcp2* gene that encodes extracellular structural components of the secretion machine of *P. betavasculorum* strains were absent in their genomes. In addition, genes encoding Sigma-fimbriae uncharacterized paralogous subunit sF-SU1, chaperone protein, usher protein, and tip adhesin forming T7SS (Chaperone/Usher pathway, CU) were identified.

##### 3.3.7.3 Conjugative and mobile elements

The genomes of *P. betavasculorum* strains were screened for the presence of integrative conjugative elements (ICEs) and integrative mobile elements (IMEs) that are known to affect the pathogenicity and lifestyle of phytopathogens (de Assis et al., 2022). In total, four different ICE elements harboring genetic determinant of T4SS and six IME elements were detected. Integrative conjugative elements were present in all of *P. betavasculorum* genomes except strain Ecb168, which harbor four different IME elements only, whereas strain SF142.2 has only ICEs. The ICEAPb1 element encoding type IV secretion pilus proteins was present in the genomes of four strains, CFBP1520, SF142.2, CFBP3291, and NPPB2795^T^, while T4SS-like element ICEPb2 harboring *vir* genes was detected in four strains CFBP1520, SF142.2, NPPB2793, and NPPB2795^T^. Of the six IMEs detected, only IMEPb1 was unique to one *P. betavasculorum* strain CFBP1520; the others were present in two or three of the analyzed genomes (**Supplementary Table S6**).

##### 3.3.7.4 Prophages

Between 6 and 10 prophage regions were detected in all *P. betavasculorum* genomes analyzed (**Supplementary Table S7**). Most genomes differed in phage regions. The exceptions were strains CFBP1520 and SF142.2, in which 10 phage regions each were detected in their genomes, nine of which were the same. Interestingly, the phage BcepMu (NC_005882) from *Burkholderia cenocepacia* was detected in all analyzed genomes. The prophage region in strain CFBP3291 encodes type II toxin-antitoxin system CcdA/CcdB.

##### 3.3.7.5 Toxins and bacteriocins

Coronofacic acid (*cfa*) biosynthetic cluster consisting of 10 genes (*cfa*1-8A and B, and *cfl*), the carotovoricin (*ctv*) biosynthetic cluster, the carbapenem (*carRABCDEFGH)* biosynthesis cluster, and phenazine antibiotic biosynthesis gene cluster (*ehpABCDEFGR*) were detected in all of the six analyzed *P. betavasculorum* genomes. The carocin biosynthesis cluster consisting of two genes *caroDK* encoding bacteriocin killing protein (carocin D) and *caroDI* bacteriocin immunity protein was present in the genomes of two strains CFBP3291 and NCPPB2795^T^ only (**Table 7**). Those two strains possess plasmid; however, carocin genes were located on the chromosome.

The phytotoxin operon of biosynthesis of coronofacic acid (*cfa*) is located in the *P. betavasculorum* in the genomic island, like in the other species, *P. atrosepticum*, *P. brasiliense*, *P. peruviense*, and *P. actinidiae* (Panda et al., 2016; Arizala and Arif, 2019).

The detected genes of bacteriocins, carotovoricin, phenazine, and carocins are not unique for *P. betavasculorum* and are also produced by other *Pectobacterium* species (Marquez-Villavicencio et al., 2011). Carotovoricin is a phage tail-like bacteriocin encoded by a cluster of genes (*ctv*) located in the genetic island containing prophage remnant genes. In *P. betavasculorum*, the cluster of genes *ctv* was identified by the Phaster platform as a region similar to prophage BcepMu from *Burkholderia cenocepacia* (NC_005882) (**Supplementary Figure S5**). Such genomic island was described earlier in other *Pectobacterium* species, *P. atrosepticum* SCRI1043 and *P. carotovorum* (Nguyen et al., 2002; Yamada et al., 2006) or *P. brasiliense* 1692 (Shyntum et al. 2019). Carbapenem is an antibiotic produced by many bacteria including various *Pectobacterium* species (Marquez-Villavicencio et al., 2011). Synthesis of this bacteriocin is regulated by quorum-sensing molecules N-(3-oxohexanoyl)-L-homoserine (OHHL) and occurs simultaneously with the production of PCWD enzymes (Põllumaa et al., 2012). Oxygenic conditions suppress it (Shyntum et al. 2019). Carocin D colicin-like bacteriocin with DNase activity is also produced by *P. carotovorum* (Roh et al., 2010).

##### 3.3.7.6 Iron acquisition systems

Two distinct gene clusters involved in iron uptake were detected in all of the *P. betavasculorum* genomes. The Achromobactin uptake cluster consists of five genes, *cbrABCD* and *acr*, while the enterobactin synthesis cluster is built from eight genes, *entAFBECD*, *cirA*, and gene putative encoding iron transporter (**Table 7**). The ability to chelate iron was confirmed for all strains by using CAS plate assay (**Supplementary Figure S5**). *P*. *betavasculorum* strains do not pose genes encoding other iron uptake systems, like ferric citrate uptake cluster (*fecIRABCDE*), heme acquisition cluster (*hasRADEF*), hemin storage cluster (*hmsHFRS*), and ferredoxin uptake cluster (*fusBACD*), which were identified in the genomes of other *Pectobacterium* species (Arizala and Arif, 2019).

##### 3.3.7.7 The production of auxin and acetoin and 2,3-butanediol, other virulence genes, and virulence regulators

The indole*-*3*-*acetic acid (IAA) and 3-hydroxy-2-butanone (3H2B) pathways gene clusters and genes encoding avirulence protein (*svx/avrXca*) necrosis inducing virulence protein/necrosis and ethylene-inducing peptide 1 (*nip/nep1*), citrate transporter (*cit1*), glucose-1-phosphatase (*appA*), ribosomal protein RplY (*rplY*) were present in all of *P. betavasculorum* genomes. What is more, 12 virulence regulators like the two-component PhoP-*PhoQ* system, *RcsCBD* phosphorelay system, Gac/Rsm system, the quorum-sensing N-acyl homoserine lactone (AHL) including ExpI, a LuxI-type AHL synthase, two AHL receptors ExpR1 and ExpR2, two-component transcriptional response virulence regulator (evr), ferric uptake regulation protein FUR, sirohydrochlorin ferrochelatase (*sirB1*), transcriptional regulators KdgR and ExpM (regulator of RpoS), transcriptional regulator SlyA, and transcriptional (co)regulator CytR were detected (**Table 7**). The production of IAA, acetoin, and 3-hydroxy-2-butanone (3H2B) was confirmed experimentally (**Supplementary Figures S2**, **S7**, and **Supplementary Table S1**). We detected the same set of genes as (Arizala and Arif, 2019) in earlier analyses, which were performed for only two *P. betavasculorum* genomes (JQHM00000000 and JQHL00000000) that were available in the GenBank. Similarly, like Arizala and Arif, 2019, in none of the six *P. betavasculorum* genomes did we detect the presence of other virulence factors, such as metal-dependent isothiocyanate hydrolases (*saxA1*, *saxA2*, and *saxA3)* or Erwinia virulence factor Evf (**Table 7**).

##### 3.3.7.8 Polymer synthesis

The newly sequenced genomes of *P. betavasculorum* strains had the same repertoire of genetic determinants responsible for biopolymer synthesis, as it was described for strains NCPPB2793 and NCPPB25^T^ by Arizala and Arif (2019). Regardless of the origin of *P. betavasculorum* strains, in all genomes, the following gene clusters encoding bacterial cellulose synthase complex (*bcsCABGEZQFR*), capsular polysaccharide (*cps*) biosynthesis, Enterobacterial Common Antigen (ECA), exopolysaccharide (EPS), and O-antigen biosynthesis and lipo-oligo/polysaccharide (LOS/LPS) were detected (**Table 7**).

##### 3.3.7.9 Production of secondary metabolites

In the genomes of all five strains of *P. betavasculoru*m, clusters of genes for the biosynthesis of 12 secondary metabolites such as coronophoric acid, 1.6-phenazinedimethanol, amonabactin P 750, linear peptides containing azol(in)e, O-antigens, siderophores, homoserine lactones, beta-lactones containing a protease inhibitor, β-lactams, bicornutin A, and rhizomide were detected.

Xenoamicin A biosynthetic gene cluster was unique for strains IFB5271 and SF142.2 that did not originate from sugar beets. Gene clusters responsible for synthesizing Le-pyrrolopyrazines were a characteristic for strain IFB5271 from sunflower, while gacamide A and holrhizin were unique for strain SF142.2 from artichoke. Strains from sugar beets have different compositions of gene clusters predicted to encode non-ribosomal peptides. For type strains NCPPB2795^T^, these were teixobactin, syringomycin, and N-myristoyl-D-asparagine/cis-7-tetradecenoyl-D-asparagine/(R)-N1-[(S)-5-oxohexan-2-yl]-2-tetradecanamidosuccinamide. Strain NCPPB2793 were nunapeptin/nunamycin, microsclerodermin, and thanamycin (**Supplementary Table S8**).

## 4 Discussion

The currently ongoing climate changes have the most significant impact in agriculture. Rising land temperatures, decreased water availability, and increased soil salinity related to, among others, the use of excessive amounts of plant protection products or fertilizers have a negative impact on plant growth and development. At the same time, intensive international trade of plant materials, such as seeds, tubers, bulbs, ornamentals, herbs, or plant food, results in the intensive spread of plant-associated bacteria, including pathogens from the *Pectobacterium* genus (Smoktunowicz et al., 2022). Climate warming makes it easier for phytopathogens to acclimatize to the new climate zone and/or settle in a new host. In order to effectively detect and monitor the spread of phytopathogens, a comprehensive genetic and phenotypic characterization and the broadest possible knowledge about their biology, adaptive abilities, and the possibility of changing hosts are necessary.

Therefore, in this study, the research was undertaken to understand the biology of *P. betavasculorum*, which causes diseases of various plants that are an important part of the food market and are also used as raw materials in biotechnology. The polyphasic analysis was performed to verify whether strains of *P. betavasculorum* are adapted for the colonization of plants with high sugar or lipid content.

The comprehensive phenotypic characteristic based on 130 phenotypic features (**Figure 2**; **Supplementary Tables S1**, **S2**) of14 strains isolated from sugar beet, potato, sunflower, and artichoke isolated in Europe, Africa, and Central America showed that *P. betavasculorum* strains differ from other *Pectobacterium* species, and strains CFBP1520 and SF142.2 from sunflower and artichoke have nearly identical biochemical properties. These observations agreed with previous studies of Thomson (1981) and Gardan et al. (2003) except the fact that strains isolated from sugar beet were phenotypically diverse and could not be differentiated from strains isolated from other plants. Strain NCPPB2795^T^ was more similar to strains CFBP1520 and SF142.2 from sunflower and artichoke than with other sugar beet strains. Strain CFBP3291 from potato was more similar to strains isolated from sugar beet. In contrast, strain Ecb168 differed significantly from all *P. betavasculorum* strains, formed smaller colonies, and was characterized by a slower growth rate.

The results of metabolic profiling of *P. betavasculorum* strains showed very high metabolic plasticity of these bacteria, which is a fundamental ability that allows them to adapt their metabolic status to specific requirements during growth in response to various stimuli or in response to stress conditions. *P. betavasculorum* strains showed the ability to use various polyols, sugars, and polymers as a building material and energy source (**Supplementary Figure S3**) and plant extracts (**Supplementary Figure S4**) regardless of the host plant from which the bacteria were isolated.

All tested strains are characterized by the same high adaptability to changing environmental conditions, showing effective growth in the temperature range from 15°C to 37°C, salinity gradient of 0%–6%, pH from 2 to 11, and in conditions of limited access to water. A similar ability to grow under osmotic stress caused by high sucrose concentration (up to 20%) is demonstrated by strains isolated from sugar beet, sunflower, and artichoke (**Supplementary Figure S9**). Those results indicate that due to their high adaptability, *P. betavasculorum* may be able to spread to various climatic zones, especially in times of ongoing climate change. In addition, due to their ability to use various nutrients, *P. betavasculorum* strains are able to colonize various plant hosts. It has been shown that the ability to grow in a minimal medium containing 10% tissue extracts from eight plant species as the only source of organic compounds (**Supplementary Figure S4**). The tissues of most of the tested plant species contain significant amounts of sugars. However, it was not observed that strains from sugar beet were characterized by a faster growth rate than strains isolated from other plants. All tested strains showed weaker growth in the presence of blueberry and pitaya extracts. This fact can be correlated with the high content of flavonoids, including anthocyanins, known for their antibacterial properties (Cisowska et al., 2011), in the tissue of these plants. However, it was not observed that the SF142.2 strain isolated from artichoke, also containing large amounts of flavonoids, had a more remarkable ability to grow in the presence of blueberry and pitaya extracts than strains from sugar beet or sunflower (**Supplementary Figure S4**).

In order to compare the pathogenicity of the tested strains, we used potato tubers and chicory leaves, as those tests are most commonly performed on those plants, which allows comparison of their virulence with other *Pectobacterium* species. All tested *P. betavasculorum* strains showed the ability to macerate potato tubers and chicory leaves in laboratory conditions, in tests in which the plants were inoculated by wounding. No differences were observed in the maceration ability of plant species depending on the plant species from which the bacteria were isolated. Strains isolated from sugar beet or potato did not cause more intense symptoms of soft rot in tests on potato tubers than strains from sunflower or artichoke. Like the artichoke strain, it did not rot the chicory leaves more severely (**Table 1**). The obtained results are not surprising as the comparative analyses of genomes revealed that all analyzed strains had the same set of genes encoding PCWD enzymes. However, it should be highlighted that even though *P. betavasculorum* strains do not possess the *pel*Y gene, they do not differ in terms of virulence from strains of other *Pectobacterium* species as was shown in our earlier work (Waleron et al., 2014).

The phenotypic analysis, metabolic profiling, and pathogenicity tests of *P. betvasculorum* strains did not show that the strains isolated from plants characterized by high or low sugar or lipid content differed much in terms of biochemical properties, virulence, or adaptability.

However, bacteria can adapt to various environmental conditions also by modifying the lipid composition of their membranes. Unsaturated fatty acids and a decrease in the fatty acids chain length increase the resistance of microorganisms to lower temperatures, especially anteiso-branching (Suutari and Laakso, 1994). Physiological tests showed that all *P. betavasculorum* strains could grow well at temperatures between 15°C and 28°C. The results of FAME analysis showed that all analyzed *P. betavasculorum* strains demonstrate the same fatty acid composition but differ in their proportions. The lack of change in the fatty acid composition may be because, for FAME analysis, all strains were cultured in the same rich medium at the same 28°C temperature, which is optimal for the growth of *P. betavasculorum*. Surprisingly, the differences in the proportions of fatty acids were observed between *P. betavasculorum* strains, and they can be connected with differences in the optimal growth temperatures of the host plants from which the bacteria were isolated. Bacterial strains isolated from sugar beets are characterized by higher amounts of UFAs than strains from sunflower and artichoke (**Figure 3A**), which correlates with the optimal growth temperatures of the host plants. Sugar beets, which are grown mainly in the northern hemisphere, show more significant growth and yield more abundantly at lower temperatures than in temperatures that are the best for growing sunflower or artichoke. The increase in UFAs during growth at cold temperatures is observed. Increased relative abundance of UFAs increases membrane fluidity at cold temperatures (Diomande et al., 2014; de Carvalho and Caramujo, 2018; Français et al., 2021). Increased proportions of UFA as growth temperature decreases have been reported for *E. coli* (Marr and Ingraham, 1962) and *Vibrio* spp (Bhakoo and Herbert, 1979). On the contrary, higher amounts of SFA than UFA were recorded for *P. betavasculorum* strains isolated from sunflower and artichoke originating from warm climates (**Figure 3A**, **Table 3**). Likewise, it was noticed that, in the case of *E. coli*, when the growth temperature was elevated from 15°C to 43°C, the ratio of saturated to unsaturated fatty acids in the membrane also increased (Sinensky, 1971; 1974). Similar observations apply to MUFA, which were observed in greater amounts in *P. betavasculorum* strains isolated from sugar beet than in strains from sunflower and artichoke (**Figure 3A**, **Table 3**). This observation is consistent with previous studies that reported higher MUFA proportions at cold than at optimal temperatures in *B. cereus* (Kaneda, 1972; Haque and Russell, 2004; de Sarrau et al., 2012; Chazarreta Cifré et al., 2013).

The total amounts of OCFA and MUFA are higher in sugar beet strains than in strains from other plants (**Figure 3B**, **Table 3**). Higher sucrose content might favor the fermentation process, leading to a decrease in the pH value. Bacteria to adapt to acidic conditions increase the OCFA and MUFA contents and increase membrane fluidity, as was observed in the case of *E. coli* (Gianotti et al., 2009). Moreover, monounsaturated fatty acids and odd-chain long fatty acids can help maintain the microbial membrane’s fluidity in anaerobic conditions (Fulco, 1983; Jenkins, 1993).

The high sucrose content available for *P. betavasculorum* strains in sugar beet tissue might result in such adaptation as lower BCFA content. It was observed that cellulolytic bacteria increase membrane fluidity by increasing BCFA in cellulose’s presence. An increase in BCFA was observed after changing from a medium rich in simple sugars to one containing cellulose. The increase in membrane fluidity allows the translocation of large polymers and the secretion of cellulolytic enzymes outside the cell to hydrolyse cellulose (Vlaeminck et al., 2005; Mitchell et al., 2023). It can be hypothesized that *P. betavasculorum* isolated from sugar beet with substantial amounts of readily available sucrose contain less BCFA than strains isolated from artichoke and sunflower, which are forced to hydrolyze polymers as a result of less availability of simpler compounds that do not require hydrolysis. It should be noted, however, that all strains tested, including those from sugar beet, showed cellulolytic activity and possessed the same number (three) of genes encoding cellulases.

*P. betavasculorum* strains Ecb168 and NCPPB2795^T^, isolated from sugar beet, had a higher proportion of unsaturated fatty acids than strains SF142.2 and CFBP1520 isolated from artichoke and sunflower, respectively. On the other hand, strains SF142.2 and CFBP1520 had more polyunsaturated and branched fatty acids, which could compensate for the lower amounts of unsaturated fatty acids. *P. betavasculorum* strains, in general, also possessed the highest amounts of fatty acids of low chain length and palmitic (16:0) and palmitoleic acids (16:1). What is more, unsaturated long-chain fatty acids increase the permeability of the membranes and cell resistance to osmotic stress (Montgomerie et al., 1973). Therefore, the higher proportions of unsaturated fatty acids in Ecb168 and NCPPB2795T strains could help them adapt to high concentration of sucrose found in sugar beetroot, which may be as high as 20% (Getz, 2000).

Finally, it can also be hypothesized that the differences in the amounts of SFA and BCFA observed between *P. betavasculorum* strains may result from the adaptation of these bacteria to use nutrients available in the host plant’s tissues for FA synthesis. Strains adapted to grow in plant tissues with a high sugar content modulate the FA composition in membranes more through the synthesis of SFA because sugars are substrates needed for their synthesis (Salvador et al., 1993), while the primers for BCFA synthesis are branched amino acids (Kaneda, 1991; Kaiser et al., 2016; Dullius et al., 2020), which is why they are more abundant in isolated strains of artichoke or sunflower than in strains derived from sugar beet.

The genetic characterization of *P. betavasculorum* showed that analyzed strains are genetically diverse, and three different fingerprinting profiles were observed. Strains CFBP152 from sunflower and SF142.2 from artichoke are almost identical and differ from the sugar beet strains. No correlations between geographic origin and year of isolation could be determined (**Table 1**, **Supplementary Figure S12A**). However, further genetic characterization of *P. betavasculorum* strains agree with the results of phenotypic analyses and shows that strains from sugar beet are not separated from strains originating from other plant species. It should be noted that observed phylogenetic relationships between *P. betavasculorum* strains depend on the genomic method used. Based on MLSA (**Supplementary Figure S11**), core protein-based phylogeny (**Figure 5**) and POCP (**Supplementary Figure S12B**) strain CFBP3291 from potato is more related to strains from sugar beet NCPPB2793, NCPPB2795, and Ecb168 and all together are a bit distant from strains CFBP1520 and SF142.2 derived from sunflower and artichoke that are most similar to each other. In contrast, ANI (**Supplementary Figure S12C**) and AAI (**Supplementary Figure S12D**) methods show that NCPPB2795 and CFBP3291 strains are more distant from other *P. betavasculorum* strains.

Pangenomic analyses allowed the detection of common and unique features for *P. betavasculorum* strains isolated from different plants. The sugar beet and potato strains do not have the *nif* operon that enabled fixation of atmospheric nitrogen but are equipped with the *hut* genes encoding enzymes allowing for histidine degradation. In the case of strains originating from the artichoke and sunflower, it is the opposite. The ability to degrade histidine is common among bacteria, and the enzymes and genes of Hut pathway are conserved. The complete catabolism of histidine to glutamate, ammonia, and either formate or formamide allows the use of histidine as a carbon and nitrogen source. Moreover, intermediates of the pathway might serve as signaling molecules that affect bacterial virulence or biofilm formation (Bender, 2012). In the presence of high sugar content, as it is in sugar beet or potato tuber tissues, the catabolic repression inhibits the histidine degradation, but under nitrogen starvation, the repression is abolished, and then, histidine degradation provides both the carbon and nitrogen necessary for bacteria growth. In the case of strains that lack *hut* genes but possess the *nif* operon, nitrogen can be obtained by its fixation from the atmosphere. *P. betavasculorum* strains isolated from plants with high sugar content (sugar beet and potato) lacked genes of atmospheric nitrogen fixation. It is common for sugar beet microbiome and is correlated with field fertilization (Wolfgang et al., 2023). Moreover, it was shown that NifA is a regulator that represses auxin synthesis. NifA mutant exhibited higher pathogenicity and root infection (Bellés-Sancho et al., 2022). It can be, therefore, a factor that increases the virulence of *P. betavasculorum* strains without *nif* operon genes on sugar beet than *P. betavasculorum* strains derived from other host plants. The presence or absence of *hut* genes can be related to the histidine content in plant tissue. The red beets, potato tubers, carrot, chicory, or eggplant contain 0.02–0.04 g of histidine per 100 g of plant tissue. A total of 100 g of sugar beet and potato starch have 0.3 g and 0.17 g of histidine, while in artichoke and sunflower seeds, this amino acid was not detected (https://fitaudit.com/categories/fvs/histidine). However, free histidine was present in sunflower leaves but was absent in the bound amino acids (Roy et al., 2013).

The interchangeable occurrence of *nif* and *hut* genes in *P. betavasculorum* strains correlated with the host plant from which they were isolated can be considered an adaptation of the bacteria to the host plants, enabling optimal use of the nutrients available in their host tissues. However, to confirm this hypothesis, further in-depth research should be conducted on the ability of *P. betavasculorum* to colonize and cause disease symptoms in various host plants.

Comparative genomics of *P. betavasculorum* with other *Pectobacterium* species revealed several unusual features of this species that might have an influence on their environmental faintness.

In the genomes of *P. betavasculorum*, the genetic determinants of enzymes allowing for the sulfoquinovose utilization were identified. Sulfoquinovose is a product of hydrolysis of plant sulfolipids, which are present in plant membranes. The enzyme sulfoquinovose isomerase carries out the isomerization of sulfoquinovose to 6-deoxy-6-sulfofructose (Liu et al., 2023). In the sulfur deficiency conditions, which are typical for intensively cultivated soils, the presence of sulfur-scavenging enzymes may be advantageous (Roy et al., 2003).

Another important features unique for *P. betavasculorum* are cytokines, which are hormones promoting plant growth (Wei et al., 2023). *P. betavasculorum* strains possess isopentenyl transferase (IPT) enzyme gene in their genomes and several other genes with IPT activity, such as tRNA-IPTs and adenylate IPTs, which can produce various forms of cytokines, including the most active form, trans-Zeatin. Cytokines produced by bacteria may promote plant growth and enhance their resistance under stress conditions (Wei et al., 2023; Pereira et al., 2024). However, some phytopathogenic bacteria increase cytokine production at the infection site, which lengthens their survival in plant tissues (Wei et al., 2023). Therefore, cytokine production may contribute to enhanced virulence.

What is more*, P. betavasculorum* strains pose allantoin permease, or urea carboxylase and allophanate hydrolase gene cluster, which enable it to extract nitrogen from purines under anaerobic conditions in nitrogen starvation (Izaguirre-Mayoral et al., 2018).

The results of pathogenomic analyses correspond well with the phenotypic characterization of the *P. betavasculorum* strains. All strains have the same wide repertoire of virulence factors and an almost identical set of genes responsible for the production of PCWDEs, toxins, bacteriocins, siderophores, plant growth hormones, and volatile compounds influencing the interaction with plants, and are equipped with a set of seven secretion systems.

It is worth noting that the genes unique to *P. betavasculorum* selected based on comparative genomics can be used to develop a method for detecting and identifying this species, which is not currently available. In consequence, the number of *P. betavasculorum* strains available in international collections is very limited. Strains isolated from sugar beet predominate. As a result, it was only possible to use three strains from other plants in our study. When more strains become available, further studies are needed.

It is important, as no methods of protection or effective eradication of *Pectobacterium* exist. An effective detection method could prevent the spread of this pathogen and limit losses in beetroot, sunflower, and potato crops. Pectinolytic bacteria can spread rapidly. For example, *Dickeya solani* and *P. brasilense* were first observed on ornamental plants and vegetables grown in greenhouses in colder climates before spreading to the field crops (Sławiak et al., 2009; van der Wolf et al., 2014; Waleron et al., 2015; Jonkheer et al., 2021; Toth et al., 2021).

Summarizing the results of the analyses carried out within this research, it can be stated that species *P. betavasculorum* is a group of bacteria that specialize to colonize and infect particular plants. However, thanks to their metabolic plasticity, bacteria can easily change the host plant and adapt to changing environmental conditions, and thus, they are able to spread on different climatic regions. Therefore, the species *P. betavasculorum* should be considered an important pathogen in agriculture, especially in the era of progressive climate change and intensification of international trade in plants, especially on the food market.

## Data availability statement

The datasets presented in this study can be found in online repositories. The names of the repository/repositories and accession number(s) can be found in the article/**Supplementary Material**.

## Author contributions

MB-B: Writing – original draft, Formal analysis, Investigation, Visualization. MS: Formal analysis, Investigation, Writing – original draft. DH: Formal analysis, Investigation, Visualization, Writing – original draft. JJ: Formal analysis, Investigation, Methodology, Resources, Validation, Visualization, Writing – original draft. MMW: Data curation, Formal analysis, Investigation, Validation, Visualization, Writing – original draft. JG: Data curation, Formal analysis, Investigation, Writing – original draft. AM: Formal analysis, Investigation, Visualization, Writing – original draft. TS: Methodology, Writing – original draft. KW: Conceptualization, Data curation, Methodology, Supervision, Writing – original draft, Writing – review & editing. MW: Conceptualization, Data curation, Formal Analysis, Funding acquisition, Methodology, Project administration, Resources, Supervision, Writing – original draft, Writing – review & editing.
