# Supplementary material for: Comparative genomics, pangenomics, and phenomic studies of Pectobacterium betavasculorum strains isolated from sugar beet, potato, sunflower, and artichoke: insights into pathogenicity, virulence determinants, and adaptation to the host plant
Source: Front Plant Sci. 2024 Mar 21;15:1352318. doi: 10.3389/fpls.2024.1352318 (PMC10991766; doi:10.3389/fpls.2024.1352318)
Supplement: Supplementary file 1 [file DataSheet_1.zip › Borowska_Beszta_et_al_supplementary_metarials.docx]

Supplementary Material

## 1. Supplementary Figures

**Figure S1.** Images of *P. betavasculorum* strain CFBP1520*.* Photographs were taken by TEM (**A**) and light microscopy of Gram-stained cells with 100× magnification (B); Pits formed by bacteria growing on the CVP medium (C).

**Figure S2.** Phenotypic characteristics of *P. betavasculorum* strains, CFBP1520, CF142.2, CFBP3291, Ecb168, CFBP5531, CFBP5536, CFBP5540, NCPPB2795^T^, NCPPB2793, NCPPB3075. The chart only considers the characteristics by which the *P. betavasculorum* strains differ from the other closely related species: *P. atrosepticum* ICMP1526^T^, *P. parmentieri* SCC3193, *P. peruviense* IFB5232^T^*, P. polonicum* DPMP315^T^, *P. punjabense* SS95^T^, *P. wasabiae* CFBP3304^T^ and *P. zantedeschiae* 9M^T^. Navy colour means a positive reaction, blue - means a weak activity, and white - means an no reaction.

**Figure S3** Growth curves of *P. betavascrulorum* strains grown in the presence of various sugars. The plots show absorbance and fluorescence measurements for three strains, *P. betavasculorum* NCPPB2795^T^; *P. betavasculorum* CFBP1520; and *P. betavasculorum* SF142.2. Error bars are standard deviations of the mean.

**Figure S4** Growth curves of *P. betavascrulorum* strains grown in the presence of various plant extracts, sugar beet, fodder beet, calla lily, blackberry, pitaya, ground cherry, black nightshade, orchid, viviparous. The plots show absorbance and fluorescence measurements for three strains, *P. betavasculorum* NCPPB2795^T^, *P. betavasculorum* CFBP1520 and *P. betavasculorum* SF142.2 (C). Error bars are standard deviations of the mean. The red line indicates the absorbance measurements that indicate the growth of bacteria and blue columns indicate the fluorescence measurements indicating the metabolism of bacteria.

**Figure S5** Homoserine lactones production by *P. betavasculoum* strains. (A) results of CAS plate assay, (B) cluster of genes encoding homoserine lactones

**Figure S6** Siderophore production by *P. betavasculoum* strains. (A) results of CAS play assay, (B) cluster of genes encoding siderophores

**Figure S7** The measurements of IAA production by P. betavasculorum strains

**Figure S8** The ability of *P. betavasculorum* strains to acclimatize to different environmental conditions (A) temperature, (B) different NaCl concentration in the medium, (C) pH, (D) different water availability resulting from the presence of PEG.

**Figure S9** Growth curves of *P. betavascrulorum* strains grown in the presence of 0.5% glucose, 0.5% sucrose, 1% sucrose, 5% sucrose, 10% sucrose, 15% sucrose, and 20% sucrose. The plots show absorbance and fluorescence measurements for three strains, *P. betavasculorum* NCPPB2795^T^ (A), *P. betavasculorum* CFBP1520, (B) and *P. betavasculorum* SF142.2 (C). Error bars are standard deviations of the mean.

**Figure S10** Statistical analysis of total FA composition in *P. betavasculorum* strains

**Figure S11** Phylogeny based on MLSA

**Figure S12** Phylogenomic analyses Core genome (A), POCP (B), ANI (C), AAI (D)

**Figure S13.** The Venn graph shows the number of genes in the core genome that were detected by GetHomologues software with three different methods: OMCL, BBDH and COG.

**Figure S14** *nif* operon, and nitrogen fixation pathway pathway in *P. betavasculorum* genomes.

**Figure S15** Histidine degradation pathway, *hut* gene cluster pathway in *P. betavasculorum* genomes.

**Figure S16** Sulfoquinovose degradation pathway in *P. betavasculorum* genomes.

**2. Supplementary Tables**

**Table S1.** Phenotypic characteristics of *P. betavasculorum* and type strains of other closely related *Pectobacterium* species (enzymatic assays, sugar fermentation diatabs)

**Table S2.** The phenotypic analysis of *P. betavasculorum* using microbial identification test panel

**Table S3** Number of genes associated with general COG functional categories. The total is based on the total number of protein coding genes in the genome.

**Table S4.** List of CDs unique for each of *P. betvasculorum* strains

**Table S5**. The list of integrative conjugative elements (ICEs) and integrative mobile elements (IMEs) found in *P. betavasculorum* genomes.

**Table S6**. The list of prophages regions detected in *P. betavasculorum* genomes using PHASTER web server http://phaster.ca/.

**Table S7.** The list of secondary metabolites detected in *P. betavasculorum* genomes with ANISMASH web server <https://antismash.secondarymetabolites.org/#!/start>
