# Supplementary material for: Comparative genomics, pangenomics, and phenomic studies of Pectobacterium betavasculorum strains isolated from sugar beet, potato, sunflower, and artichoke: insights into pathogenicity, virulence determinants, and adaptation to the host plant
Source: Front Plant Sci. 2024 Mar 21;15:1352318. doi: 10.3389/fpls.2024.1352318 (PMC10991766; doi:10.3389/fpls.2024.1352318)
Supplement: Supplementary file 1 [file DataSheet_1.zip › Figure S4.pdf]

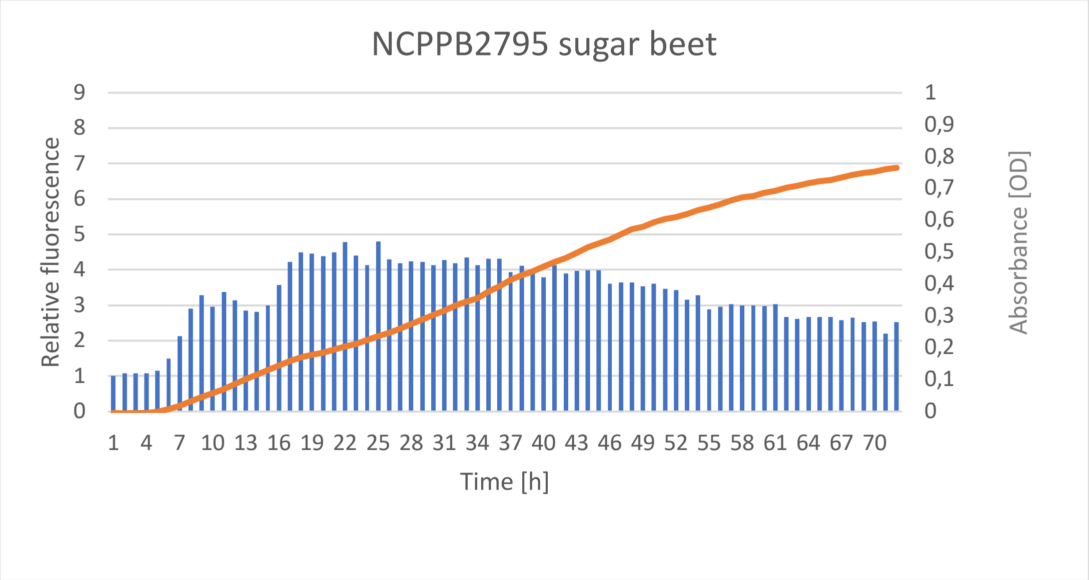
NCPPB2795 sugar beet
Relative fluorescence
9
8
7
6
5
4
3
2
1
0
1
0,9
0,8
0,7
0,6
0,5
0,4
0,3
0,2
0,1
0
Absorbance [OD]
1 4 7 10 13 16 19 22 25 28 31 34 37 40 43 46 49 52 55 58 61 64 67 70
Time [h]

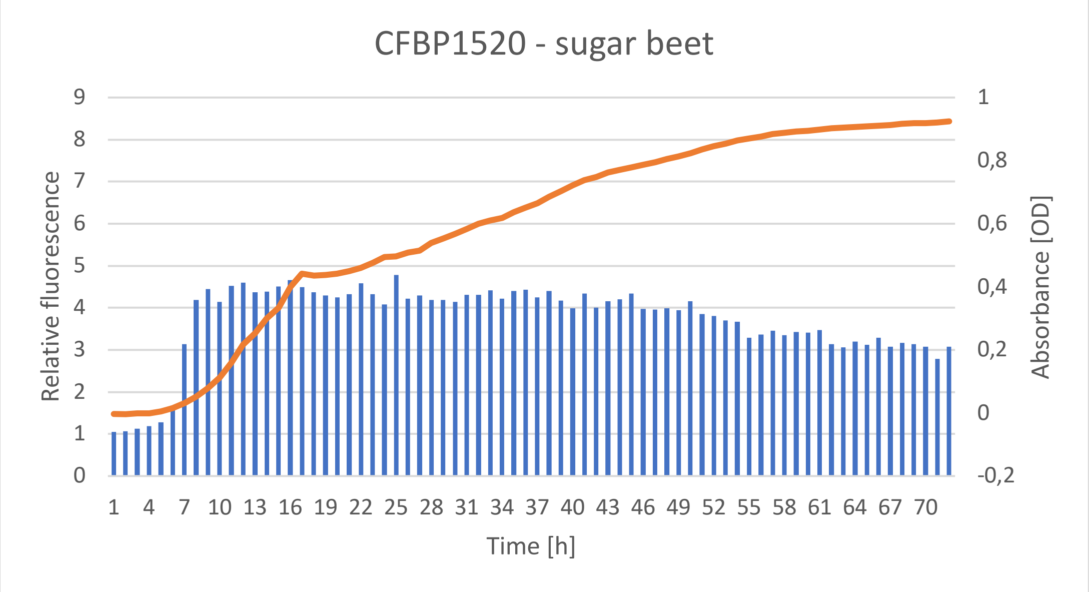
CFBP1520 - sugar beet
Relative fluorescence
9
8
7
6
5
4
3
2
1
0
1
0,8
0,6
0,4
0,2
0
-0,2
Absorbance [OD]
1 4 7 10 13 16 19 22 25 28 31 34 37 40 43 46 49 52 55 58 61 64 67 70
Time [h]

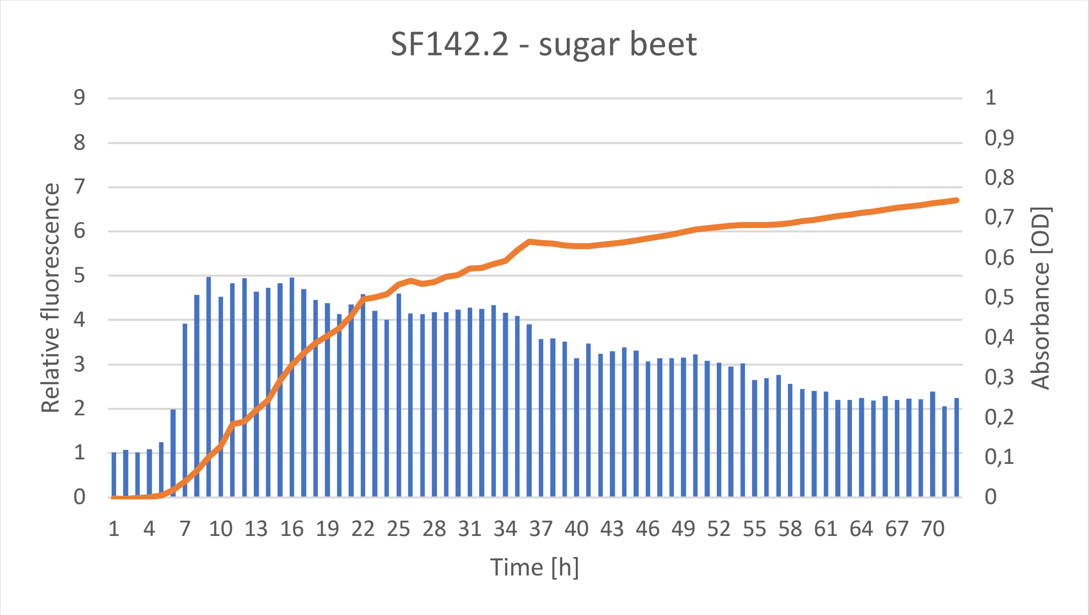
SF142.2 - sugar beet
Relative fluorescence
9
8
7
6
5
4
3
2
1
0
1
0,9
0,8
0,7
0,6
0,5
0,4
0,3
0,2
0,1
0
Absorbance [OD]
1 4 7 10 13 16 19 22 25 28 31 34 37 40 43 46 49 52 55 58 61 64 67 70
Time [h]

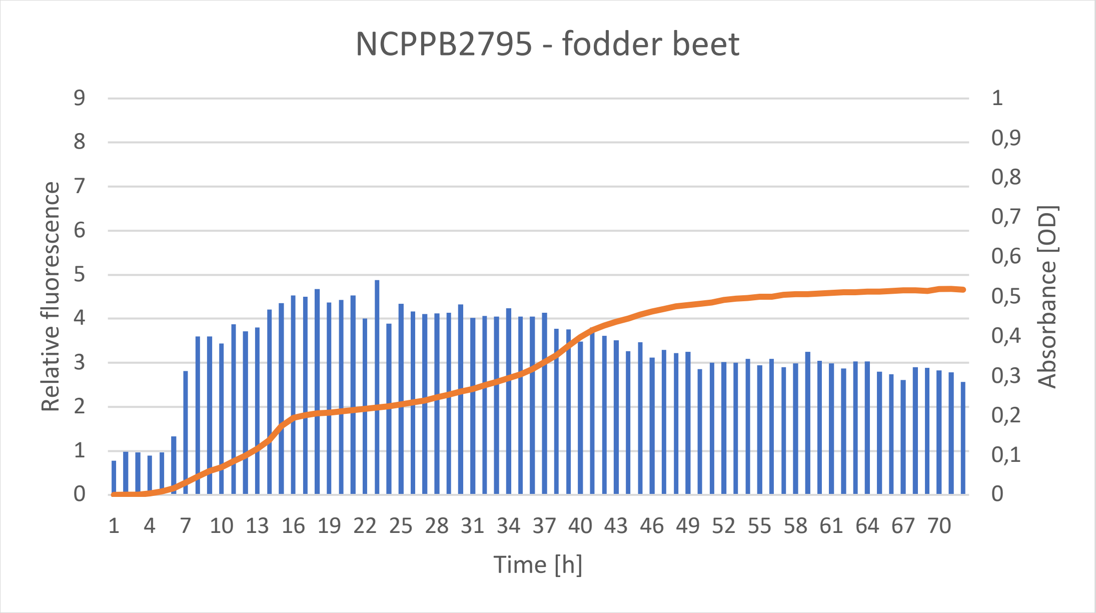
NCPPB2795 - fodder beet
Relative fluorescence
Absorbance [OD]
Time [h]
0
1
2
3
4
5
6
7
8
9
0
0,1
0,2
0,3
0,4
0,5
0,6
0,7
0,8
0,9
1
1 4 7 10 13 16 19 22 25 28 31 34 37 40 43 46 49 52 55 58 61 64 67 70

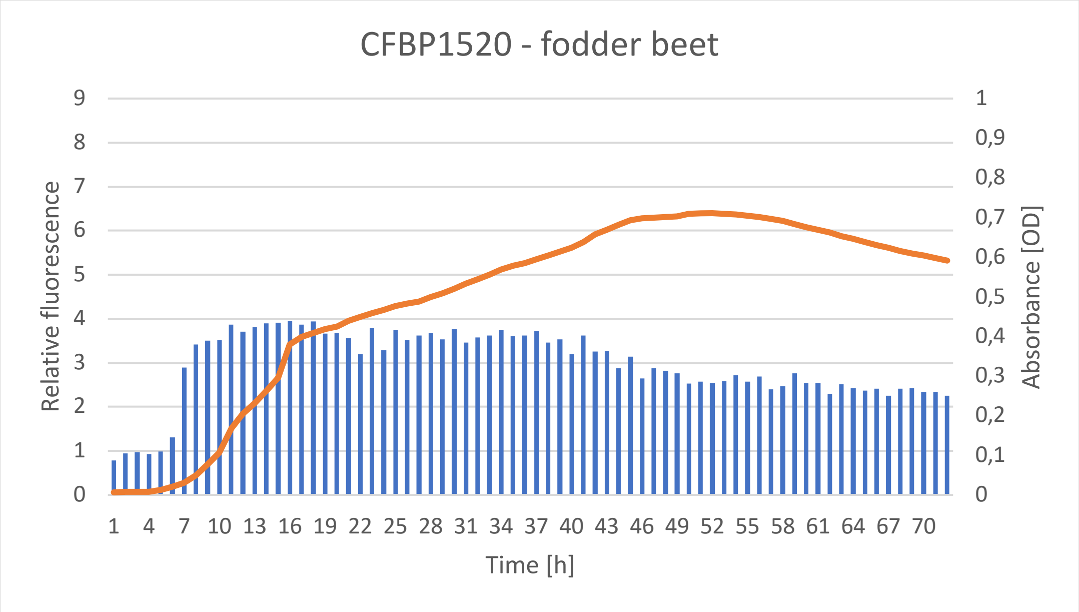
CFBP1520 - fodder beet
Relative fluorescence
Absorbance [OD]
Time [h]
0
1
2
3
4
5
6
7
8
9
0
0,1
0,2
0,3
0,4
0,5
0,6
0,7
0,8
0,9
1
1 4 7 10 13 16 19 22 25 28 31 34 37 40 43 46 49 52 55 58 61 64 67 70

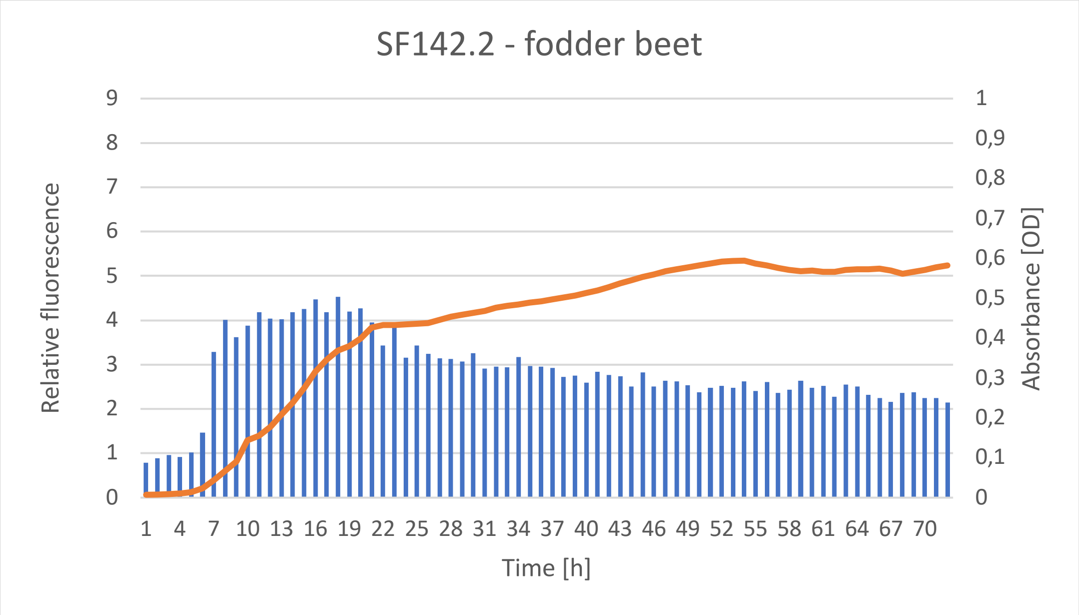
SF142.2 - fodder beet
Relative fluorescence
Absorbance [OD]
Time [h]
0
1
2
3
4
5
6
7
8
9
0
0,1
0,2
0,3
0,4
0,5
0,6
0,7
0,8
0,9
1
1 4 7 10 13 16 19 22 25 28 31 34 37 40 43 46 49 52 55 58 61 64 67 70

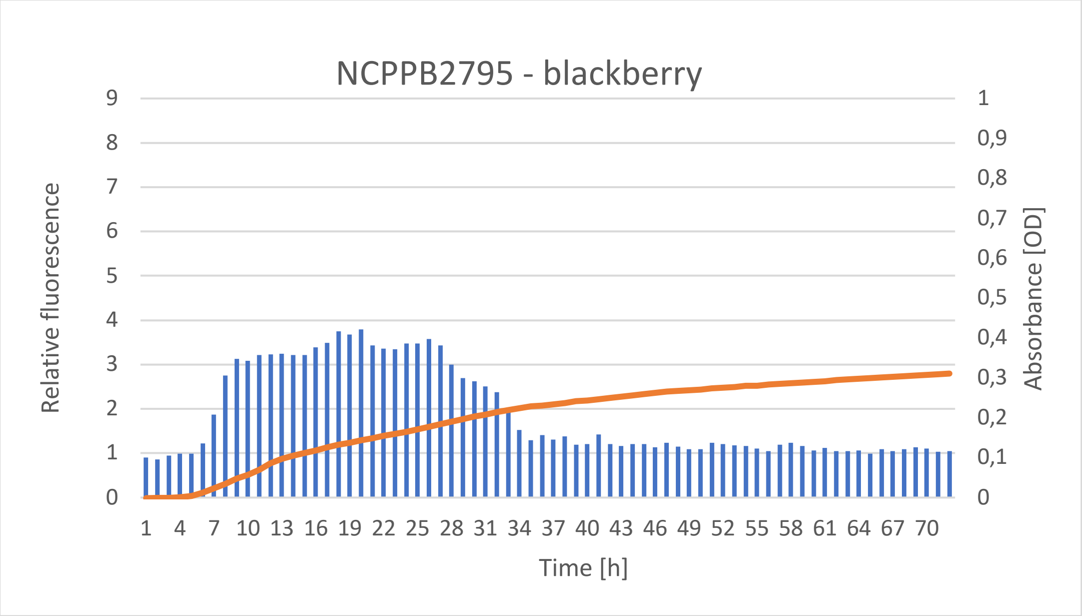
NCPPB2795 - blackberry
Relative fluorescence
Absorbance [OD]
Time [h]

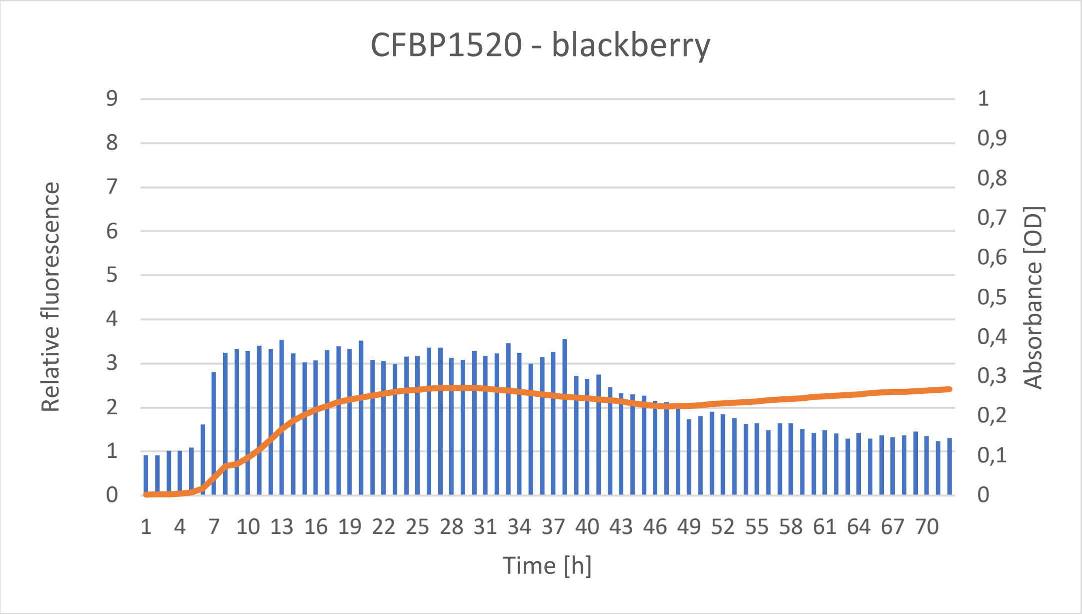
CFBP1520 - blackberry
Relative fluorescence
Absorbance [OD]
Time [h]

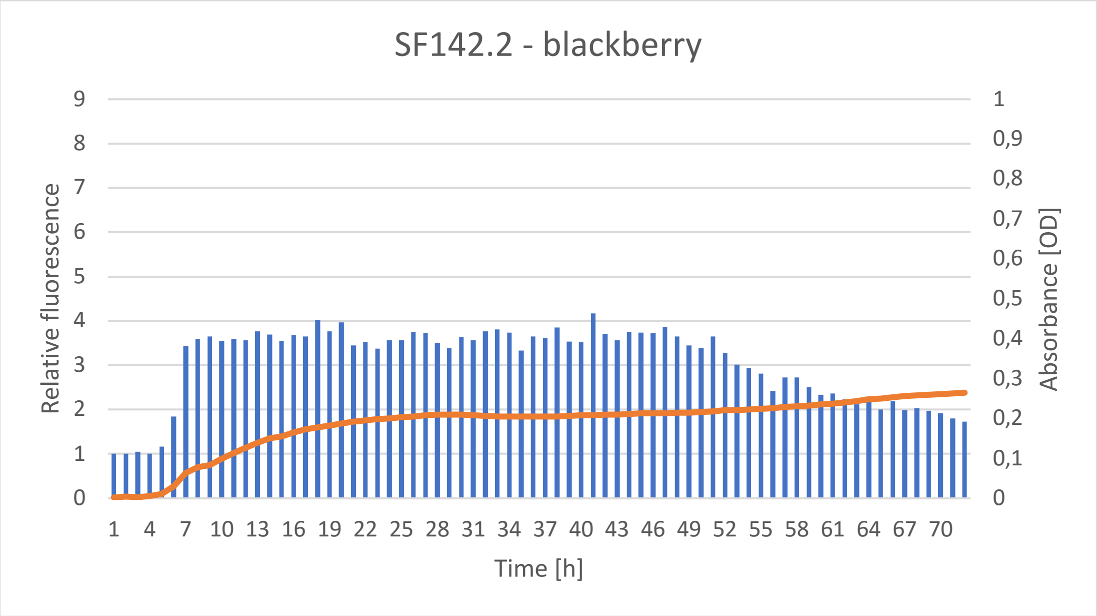
SF142.2 - blackberry
Relative fluorescence
Absorbance [OD]
Time [h]

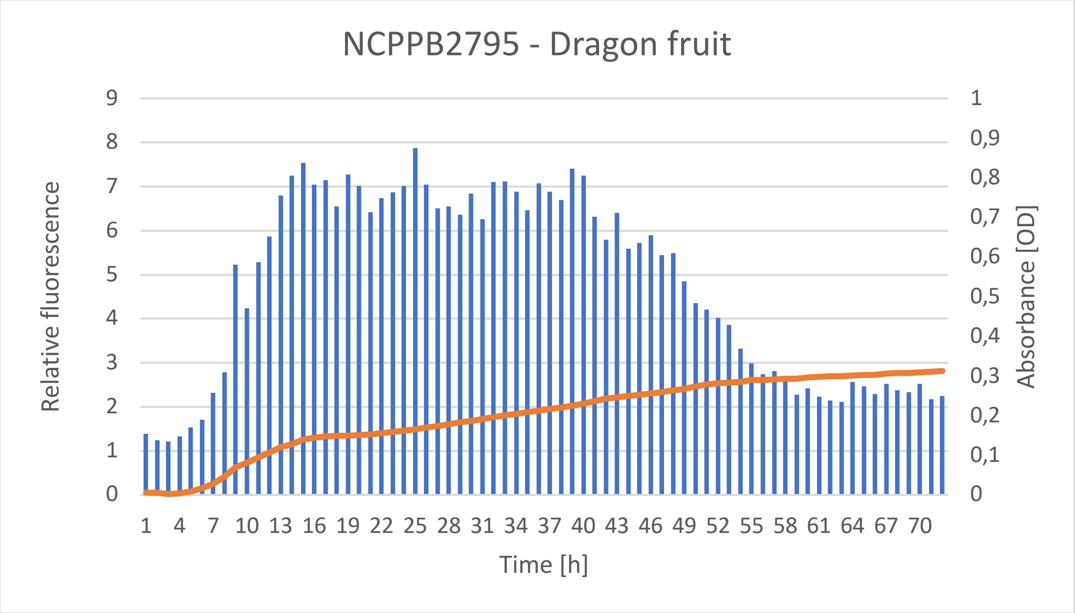
NCPPB2795 - Dragon fruit
Relative fluorescence
Absorbance [OD]
Time [h]
0
1
2
3
4
5
6
7
8
9
0
0,1
0,2
0,3
0,4
0,5
0,6
0,7
0,8
0,9
1
1 4 7 10 13 16 19 22 25 28 31 34 37 40 43 46 49 52 55 58 61 64 67 70

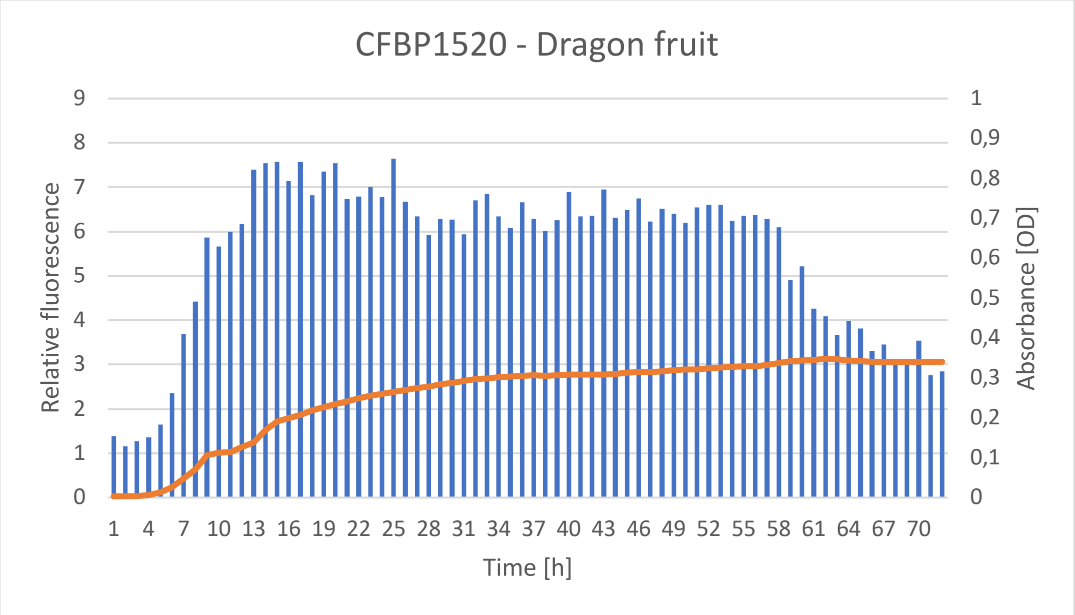
CFBP1520 - Dragon fruit
Relative fluorescence
Absorbance [OD]
Time [h]
0
1
2
3
4
5
6
7
8
9
0
0,1
0,2
0,3
0,4
0,5
0,6
0,7
0,8
0,9
1
1 4 7 10 13 16 19 22 25 28 31 34 37 40 43 46 49 52 55 58 61 64 67 70

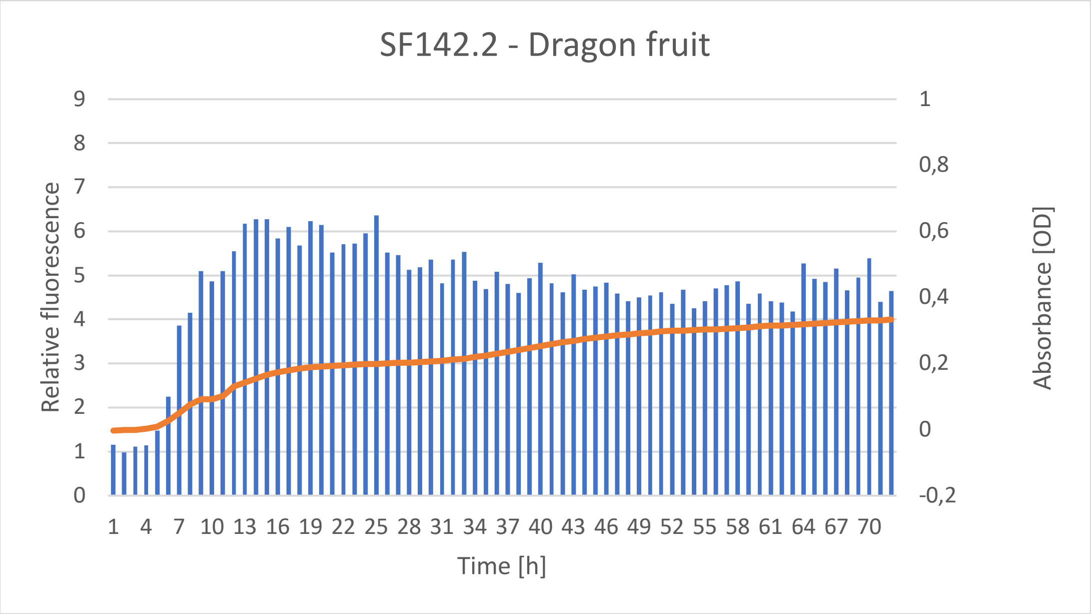
SF142.2 - Dragon fruit
Relative fluorescence
Absorbance [OD]
Time [h]
0
1
2
3
4
5
6
7
8
9
-0,2
0
0,2
0,4
0,6
0,8
1
1 4 7 10 13 16 19 22 25 28 31 34 37 40 43 46 49 52 55 58 61 64 67 70

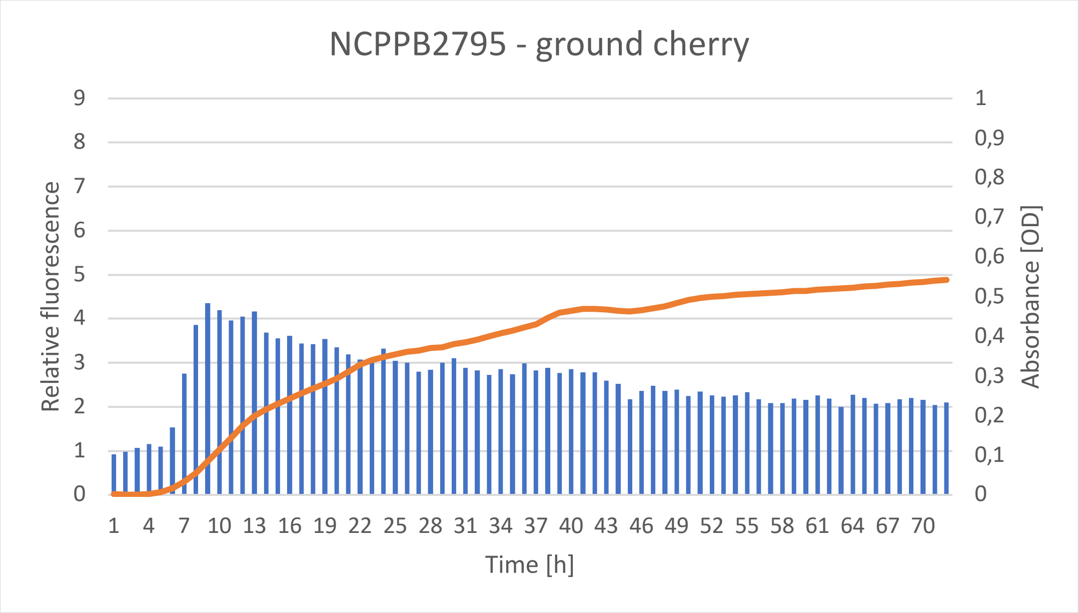
NCPPB2795 - ground cherry
Relative fluorescence
Absorbance [OD]
Time [h]
9
8
7
6
5
4
3
2
1
0
1
0,9
0,8
0,7
0,6
0,5
0,4
0,3
0,2
0,1
0
1 4 7 10 13 16 19 22 25 28 31 34 37 40 43 46 49 52 55 58 61 64 67 70

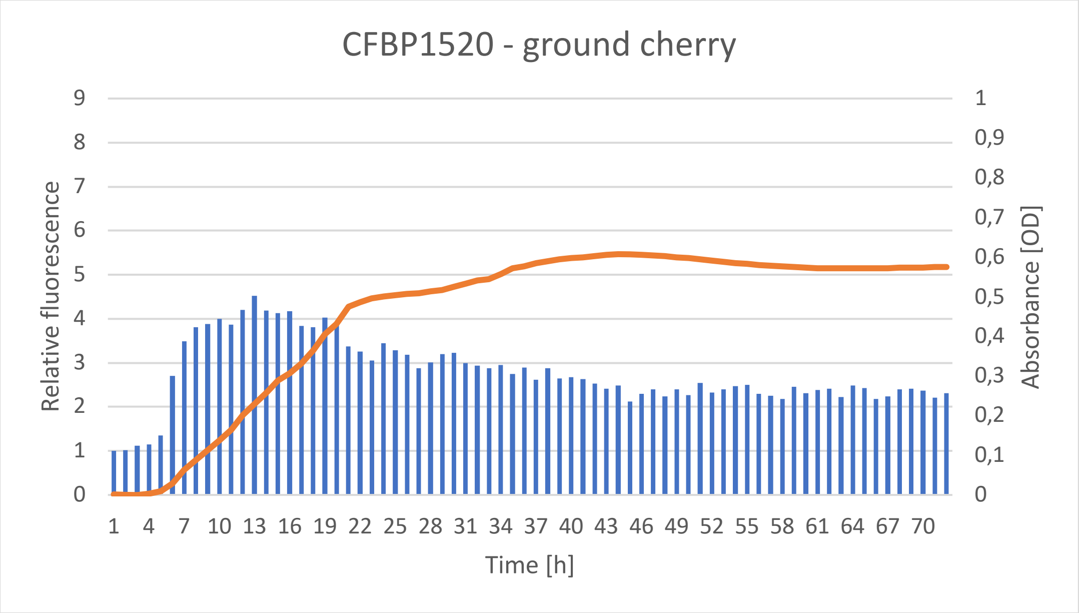
CFBP1520 - ground cherry
Relative fluorescence
Absorbance [OD]
Time [h]
9
8
7
6
5
4
3
2
1
0
1
0,9
0,8
0,7
0,6
0,5
0,4
0,3
0,2
0,1
0
1 4 7 10 13 16 19 22 25 28 31 34 37 40 43 46 49 52 55 58 61 64 67 70

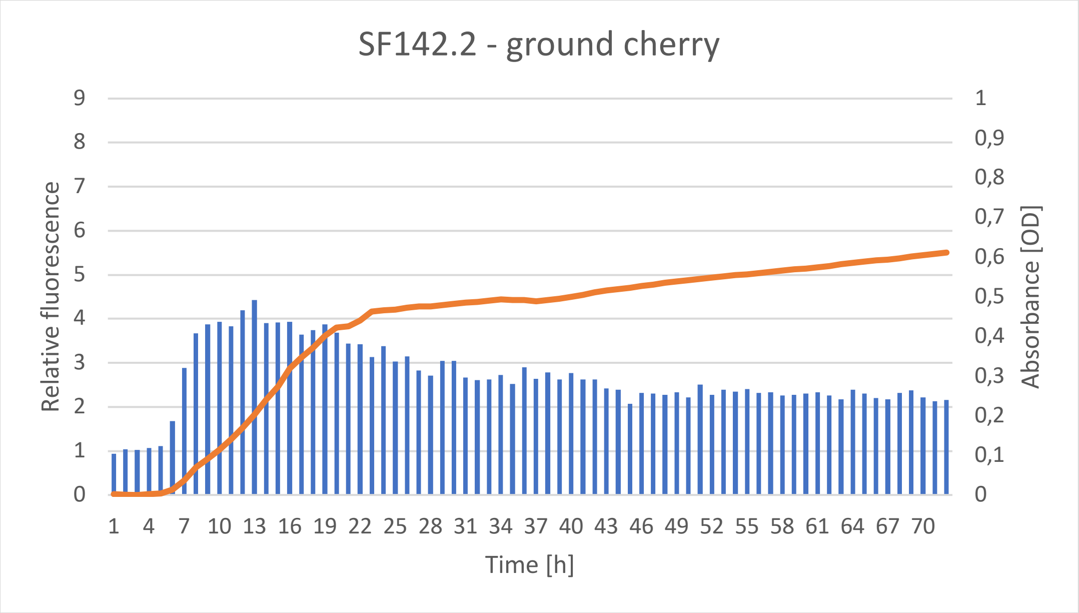
SF142.2 - ground cherry
Relative fluorescence
Absorbance [OD]
Time [h]
9
8
7
6
5
4
3
2
1
0
1
0,9
0,8
0,7
0,6
0,5
0,4
0,3
0,2
0,1
0
1 4 7 10 13 16 19 22 25 28 31 34 37 40 43 46 49 52 55 58 61 64 67 70

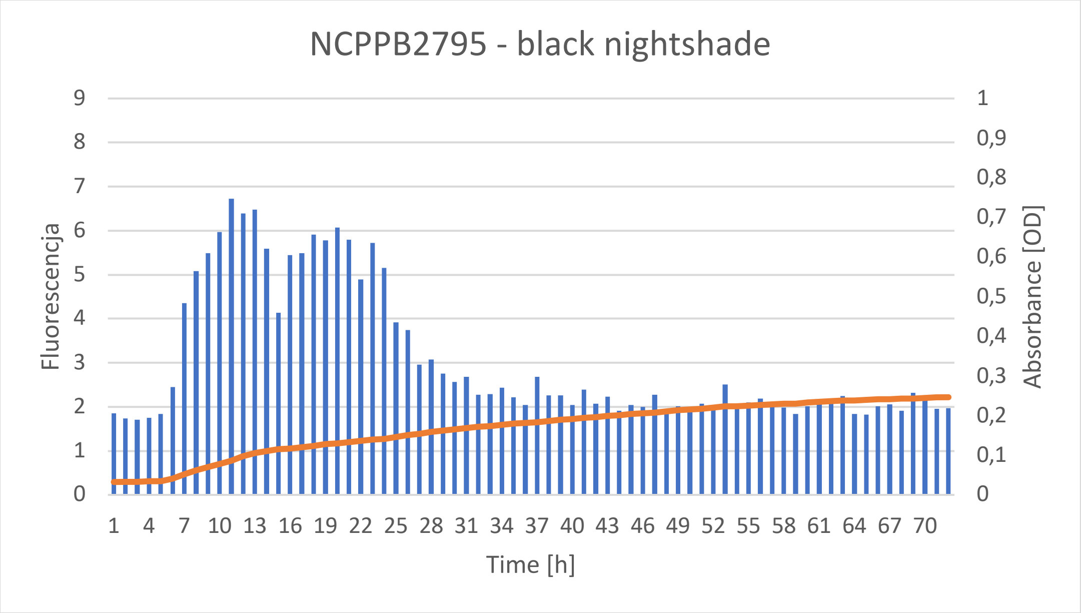
NCPPB2795 - black nightshade
Fluorescencja
Absorbance [OD]
Time [h]
0
1
2
3
4
5
6
7
8
9
0
0,1
0,2
0,3
0,4
0,5
0,6
0,7
0,8
0,9
1
1 4 7 10 13 16 19 22 25 28 31 34 37 40 43 46 49 52 55 58 61 64 67 70

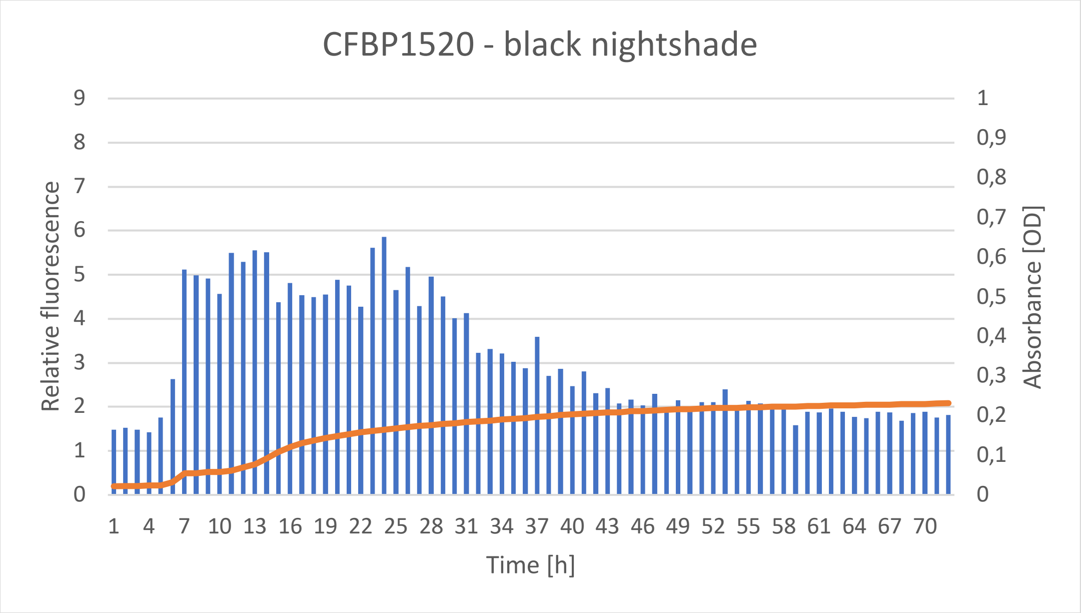
CFBP1520 - black nightshade
Relative fluorescence
Absorbance [OD]
Time [h]
0
1
2
3
4
5
6
7
8
9
0
0,1
0,2
0,3
0,4
0,5
0,6
0,7
0,8
0,9
1
1 4 7 10 13 16 19 22 25 28 31 34 37 40 43 46 49 52 55 58 61 64 67 70

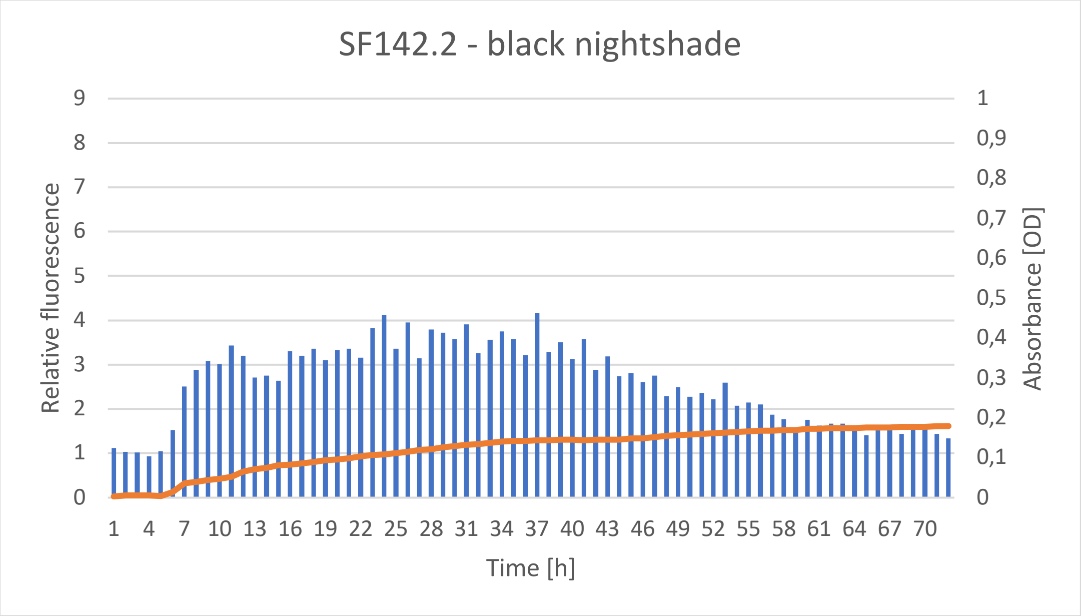
SF142.2 - black nightshade
Relative fluorescence
Absorbance [OD]
Time [h]
0
1
2
3
4
5
6
7
8
9
0
0,1
0,2
0,3
0,4
0,5
0,6
0,7
0,8
0,9
1
1 4 7 10 13 16 19 22 25 28 31 34 37 40 43 46 49 52 55 58 61 64 67 70

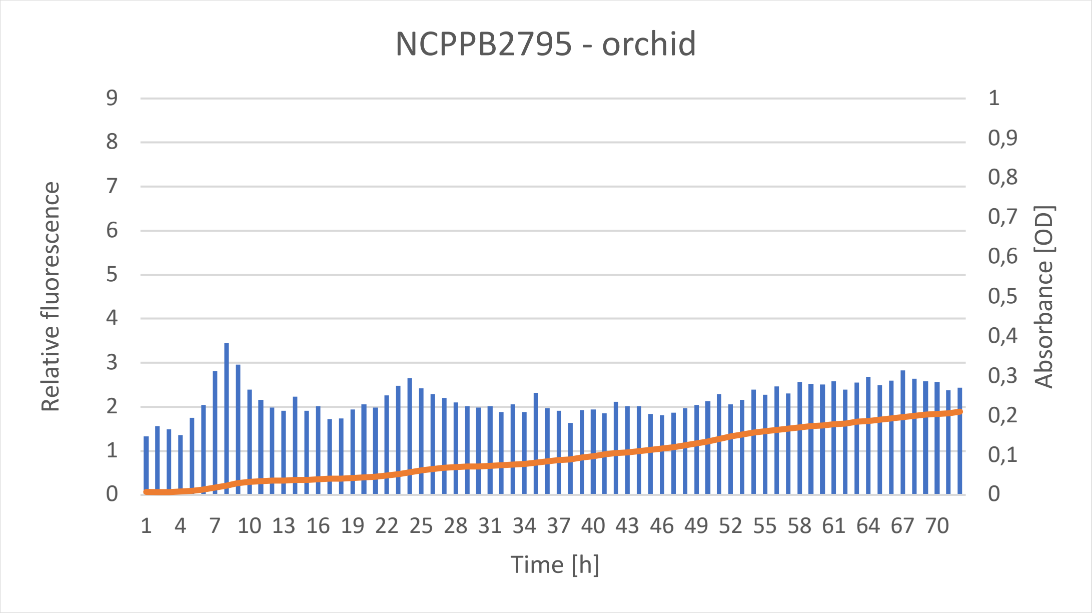
NCPPB2795 - orchid
Relative fluorescence
Absorbance [OD]
Time [h]

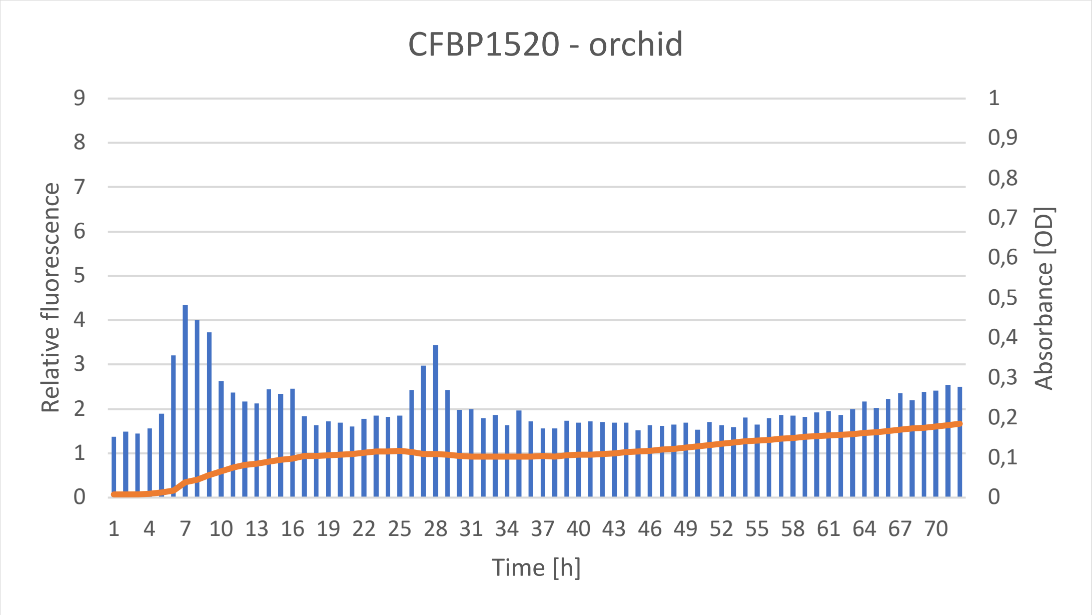
CFBP1520 - orchid
Relative fluorescence
Absorbance [OD]
Time [h]

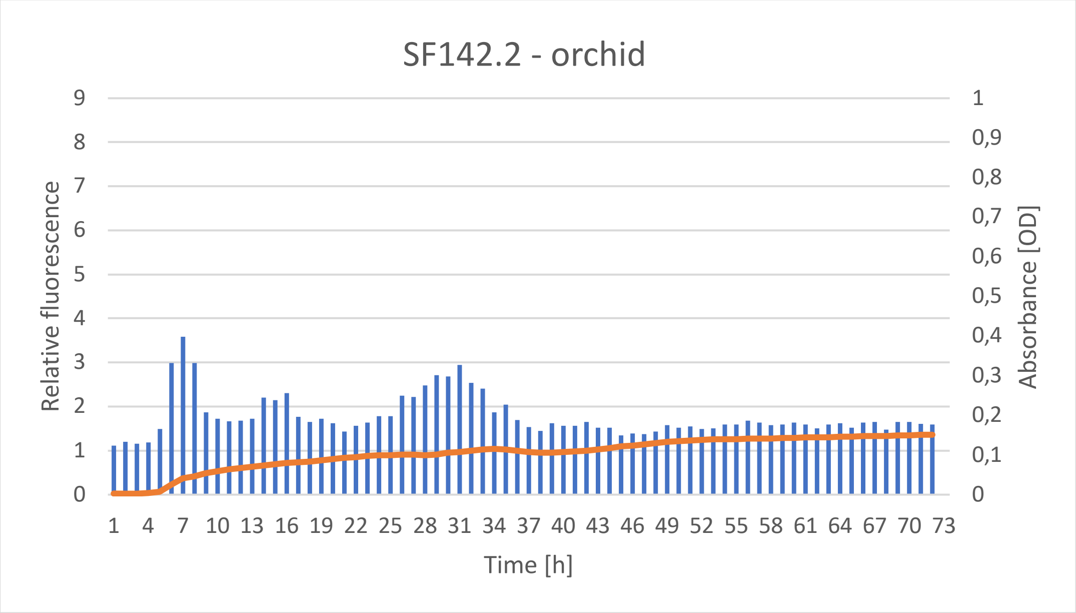
SF142.2 - orchid
Relative fluorescence
Absorbance [OD]
Time [h]

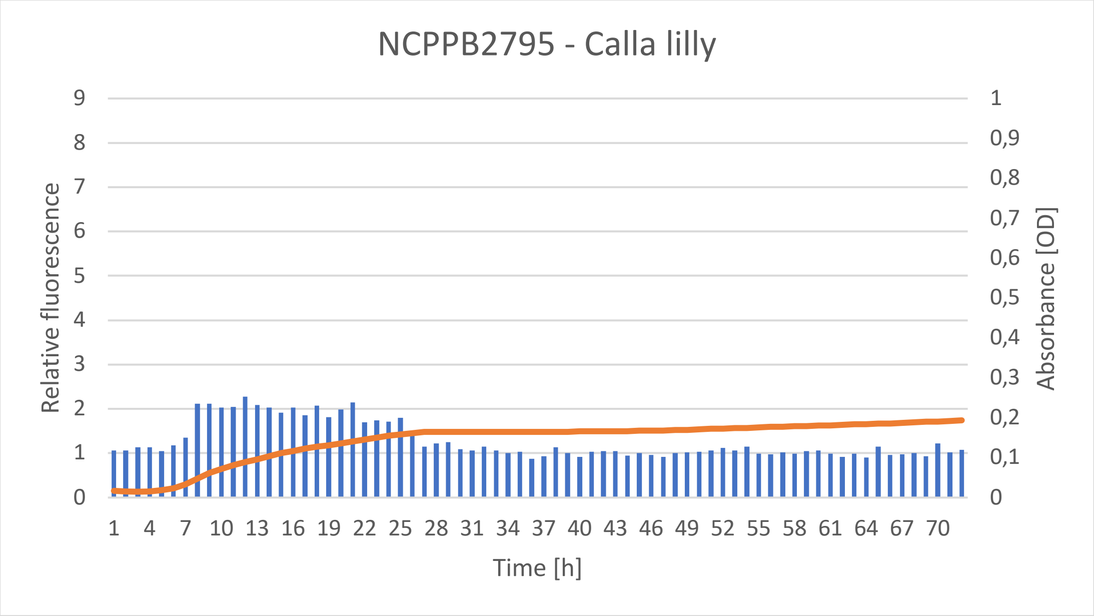
NCPPB2795 - Calla lilly
Relative fluorescence
Absorbance [OD]
Time [h]
0
1
2
3
4
5
6
7
8
9
0
0,1
0,2
0,3
0,4
0,5
0,6
0,7
0,8
0,9
1
1 4 7 10 13 16 19 22 25 28 31 34 37 40 43 46 49 52 55 58 61 64 67 70

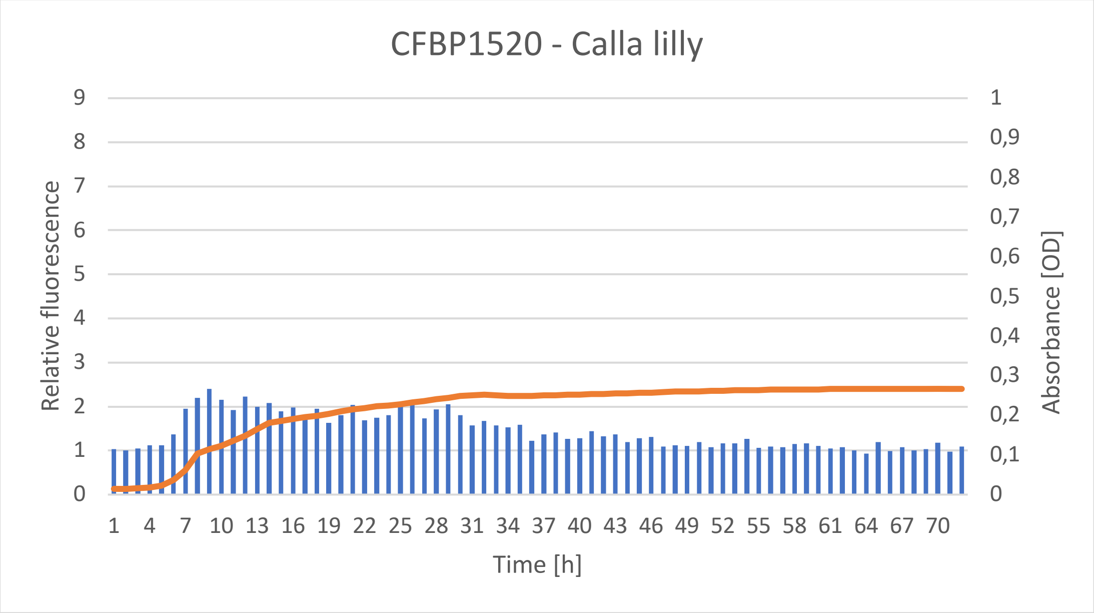
CFBP1520 - Calla lilly
Relative fluorescence
Absorbance [OD]
Time [h]
0
1
2
3
4
5
6
7
8
9
0
0,1
0,2
0,3
0,4
0,5
0,6
0,7
0,8
0,9
1
1 4 7 10 13 16 19 22 25 28 31 34 37 40 43 46 49 52 55 58 61 64 67 70

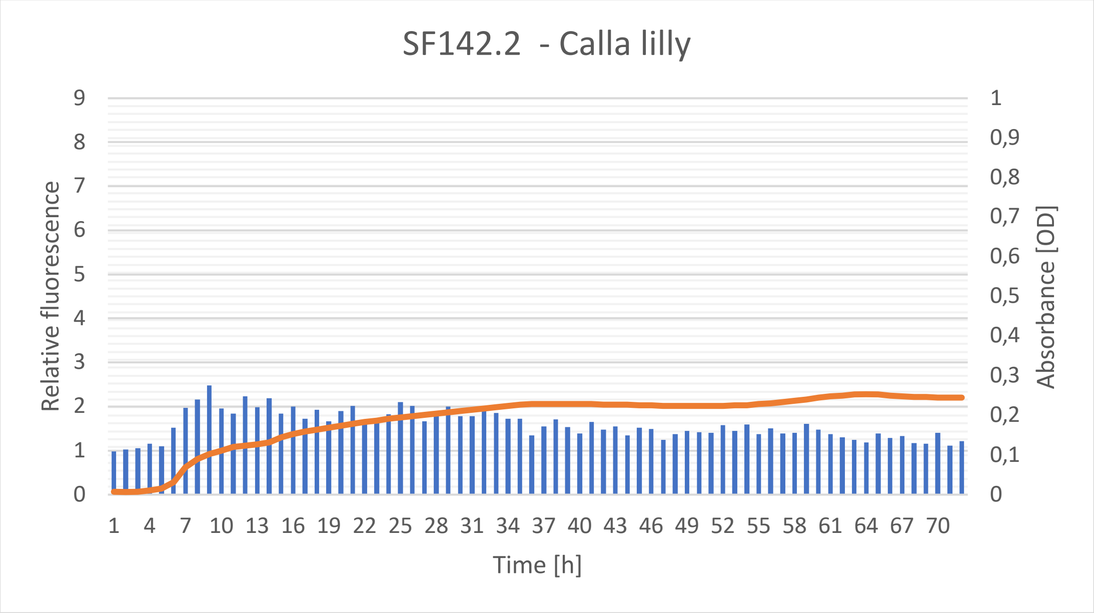
SF142.2 - Calla lilly
Relative fluorescence
Absorbance [OD]
Time [h]
0
1
2
3
4
5
6
7
8
9
0
0,1
0,2
0,3
0,4
0,5
0,6
0,7
0,8
0,9
1
1 4 7 10 13 16 19 22 25 28 31 34 37 40 43 46 49 52 55 58 61 64 67 70

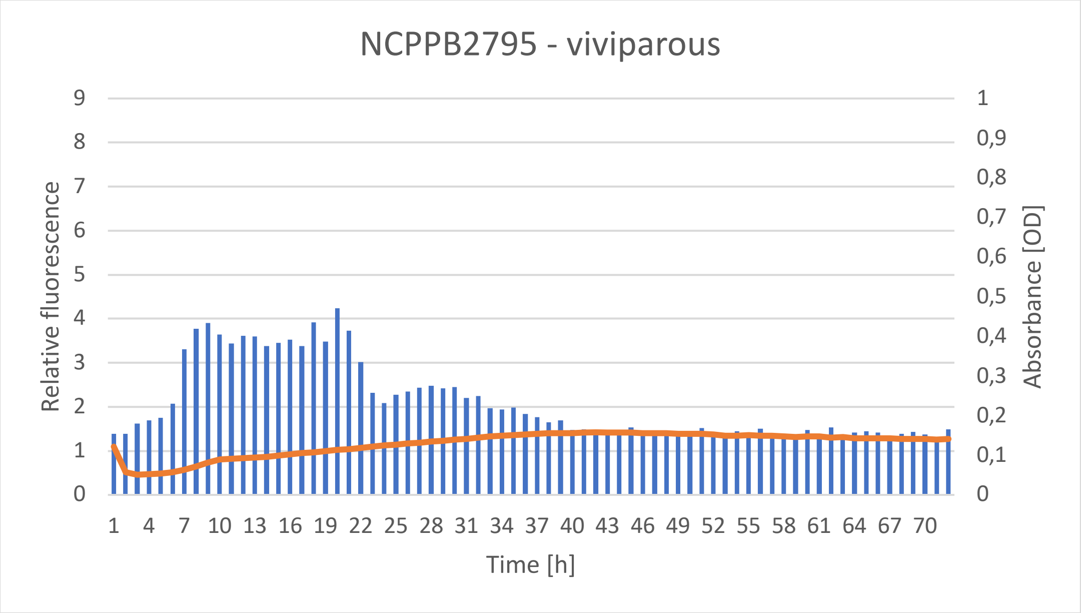
NCPPB2795 - viviparous
Relative fluorescence
Absorbance [OD]
Time [h]

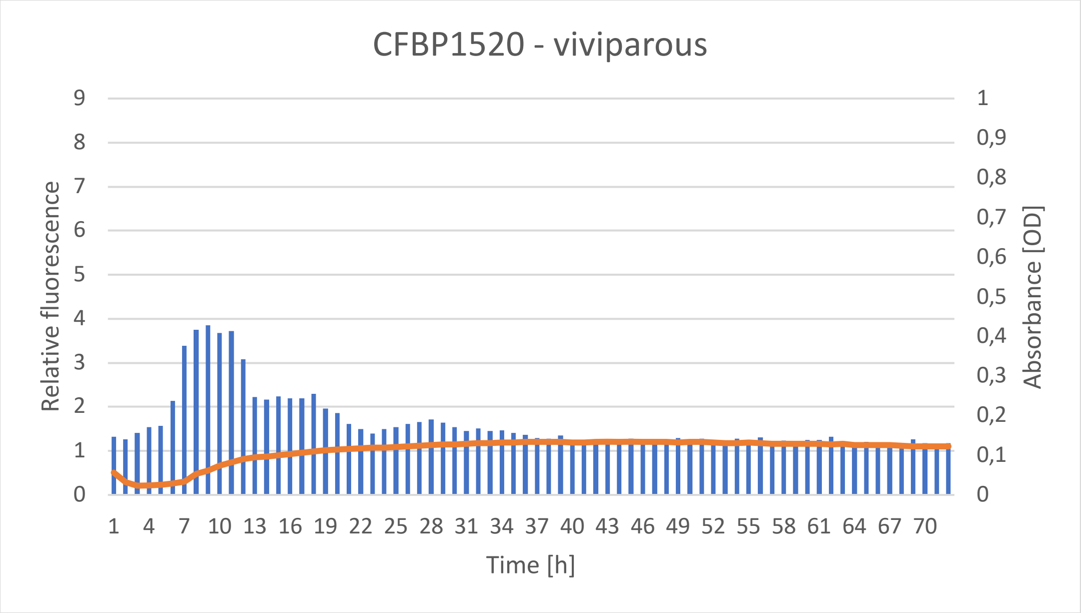
CFBP1520 - viviparous
Relative fluorescence
Absorbance [OD]
Time [h]

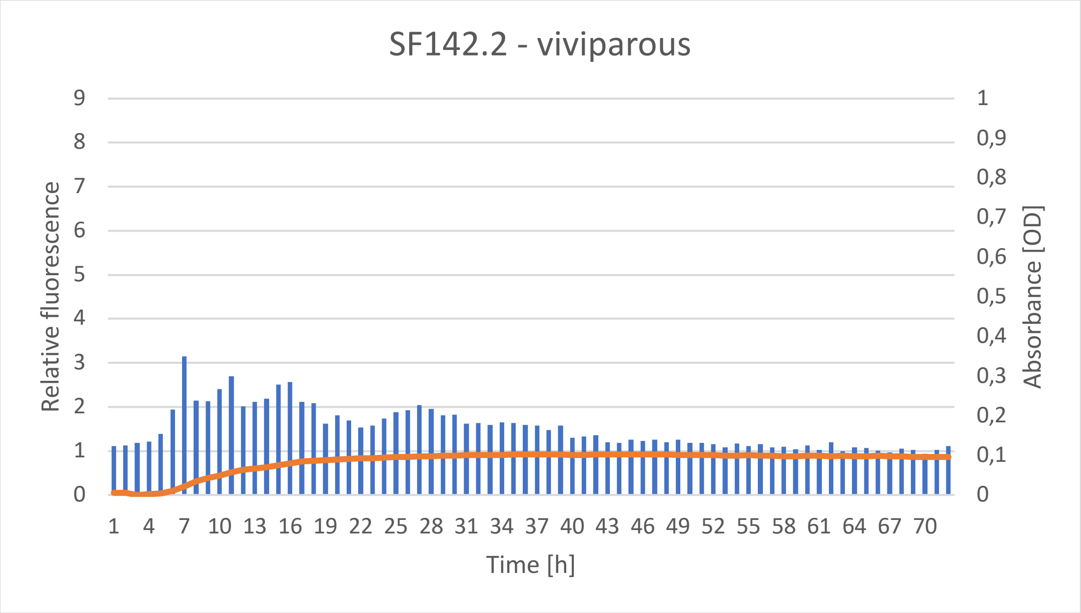
SF142.2 - viviparous
Relative fluorescence
Absorbance [OD]
Time [h]

Absorbance [OD] growth of bacteria

The relative fluorecence – metabolism of bacteria
