# Supplementary figures and images for: Comparative genomics, pangenomics, and phenomic studies of Pectobacterium betavasculorum strains isolated from sugar beet, potato, sunflower, and artichoke: insights into pathogenicity, virulence determinants, and adaptation to the host plant
Source: Front Plant Sci. 2024 Mar 21;15:1352318. doi: 10.3389/fpls.2024.1352318 (PMC10991766; doi:10.3389/fpls.2024.1352318)

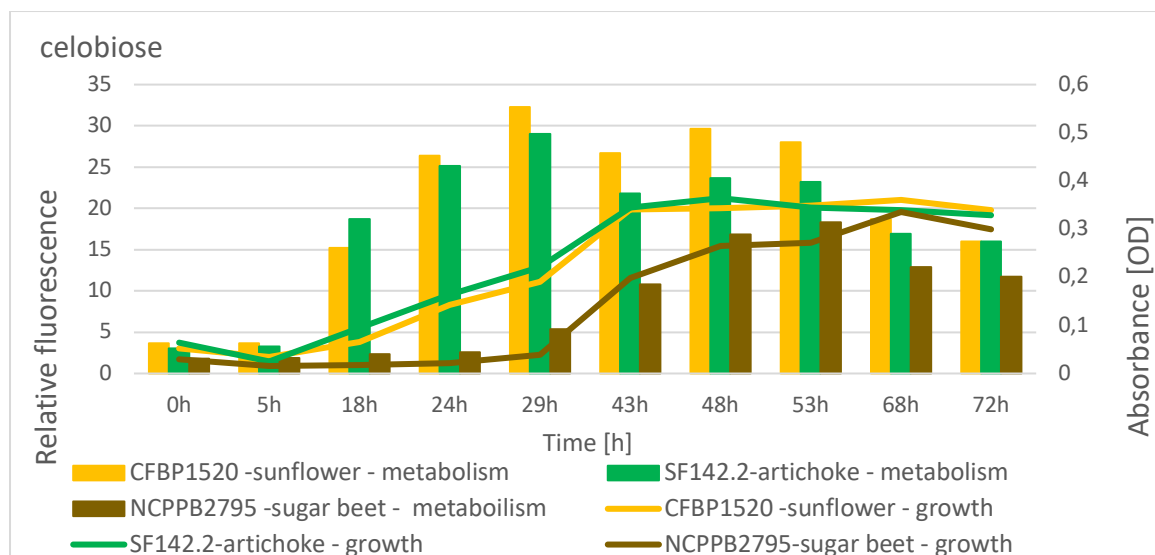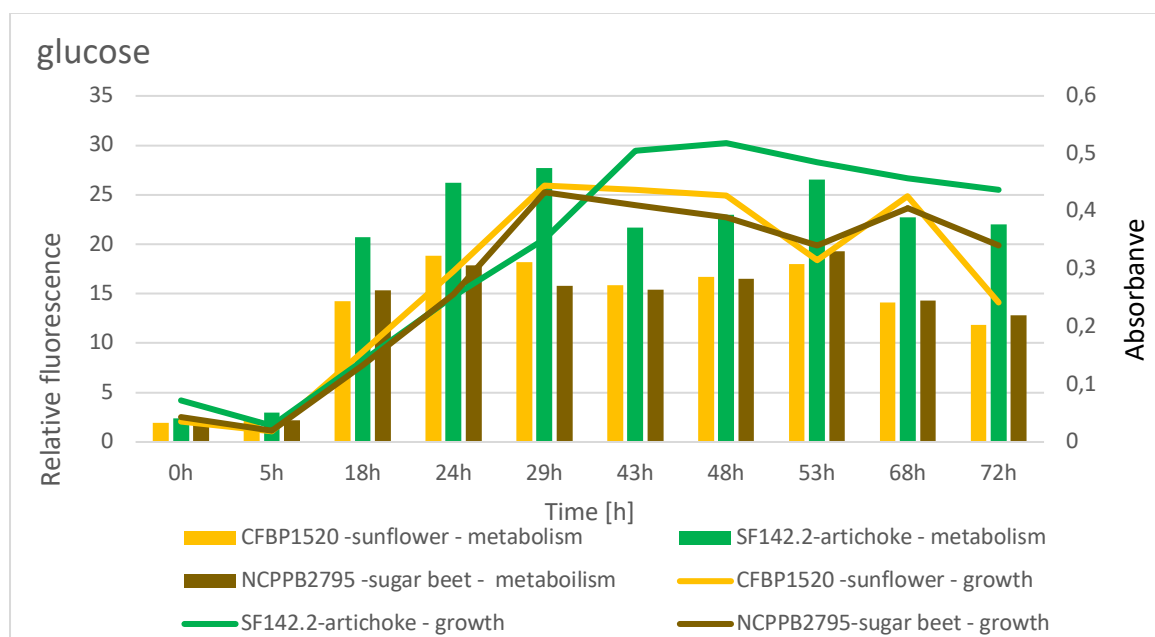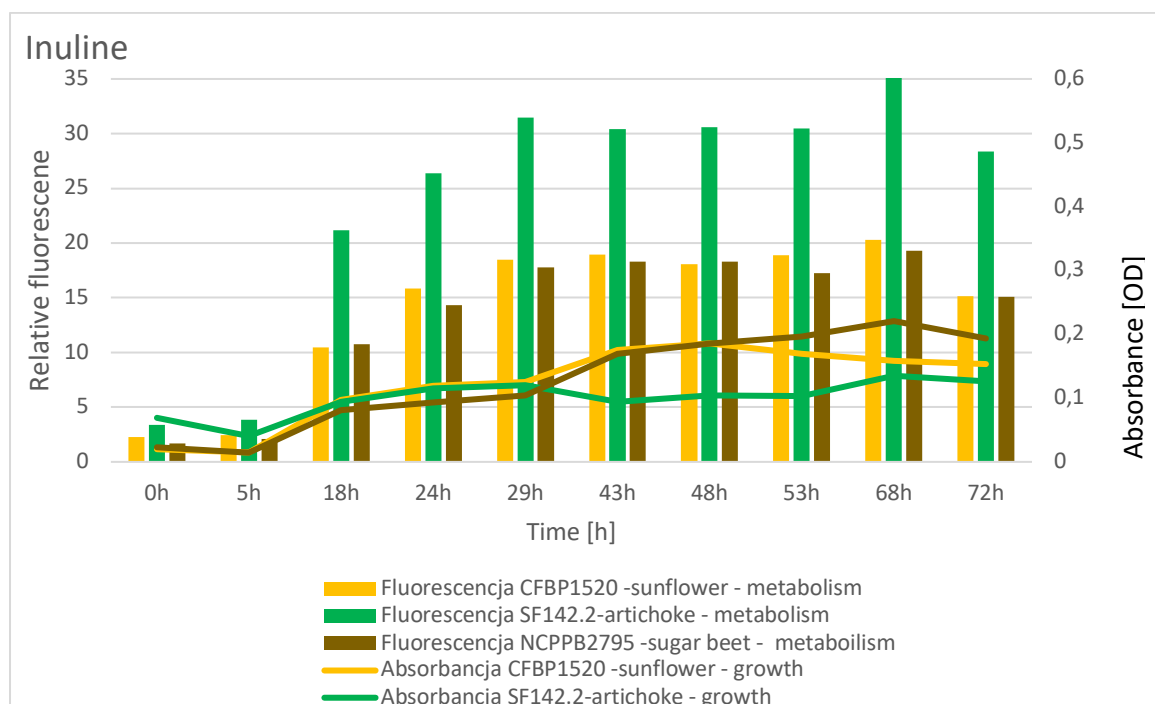

## Xylose

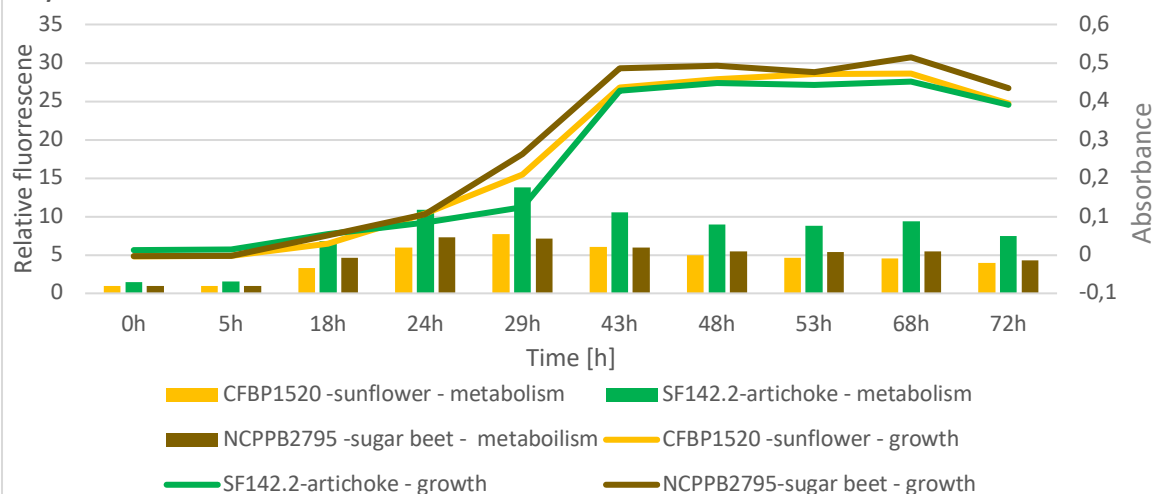

## Lactose

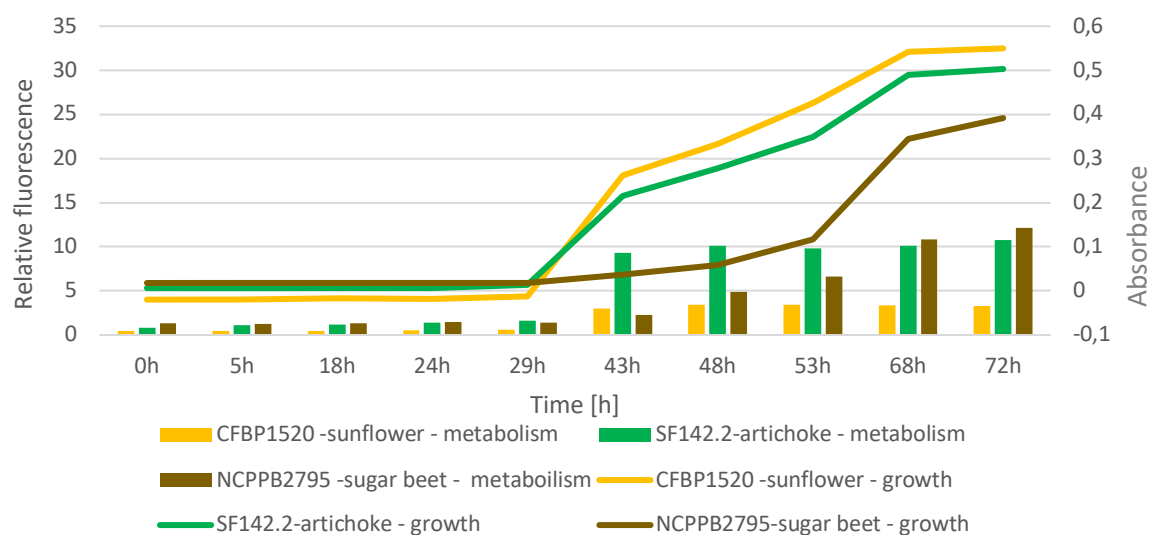

## Maltose

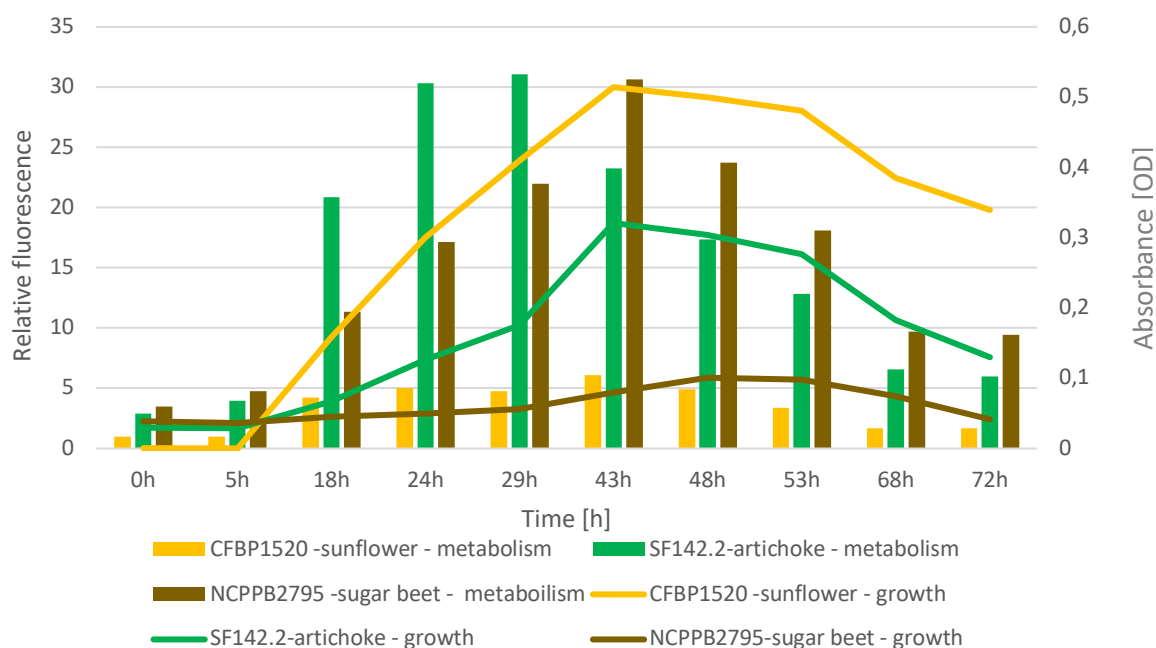

## Melibiose

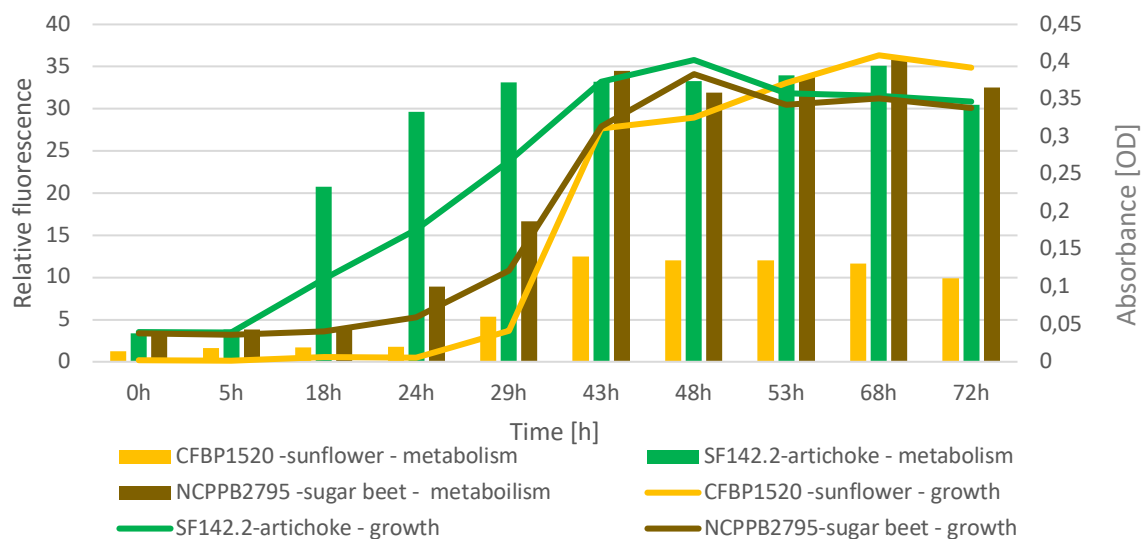

## Palatinose

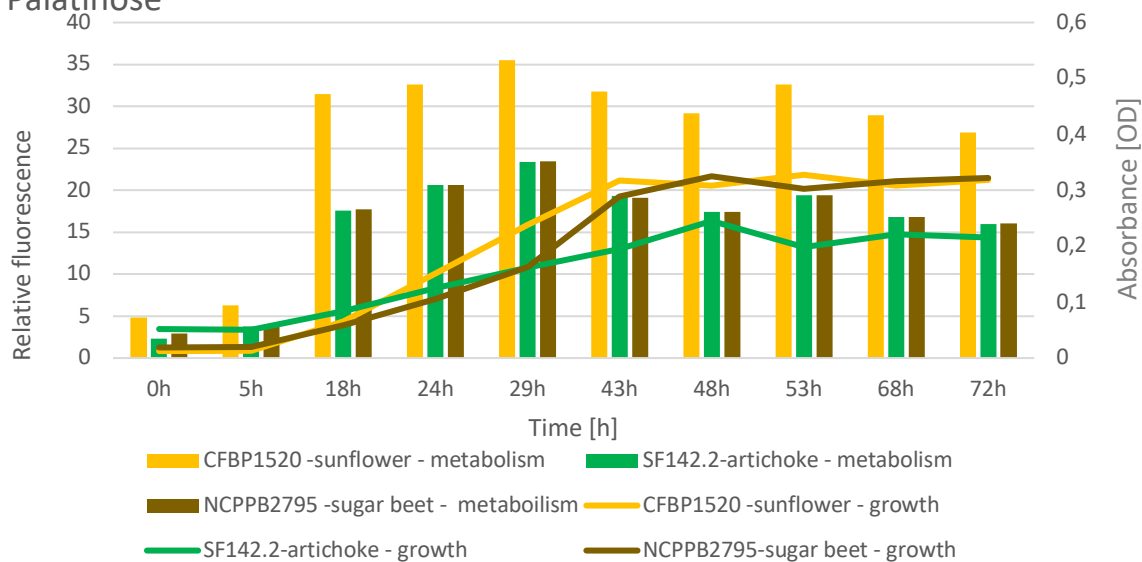

## Rhamnose

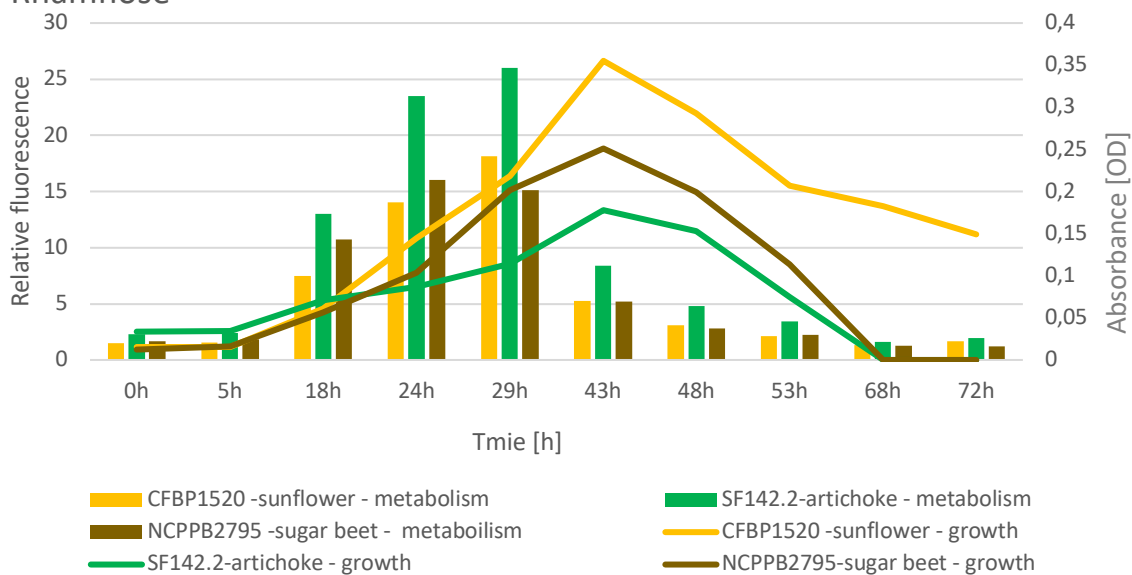

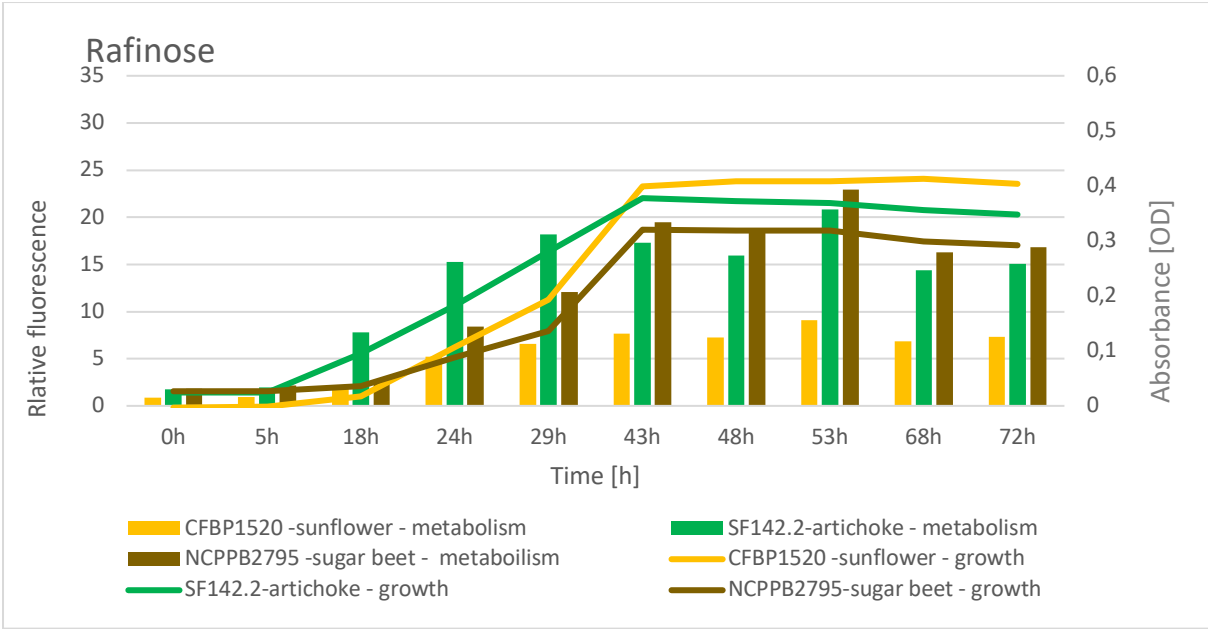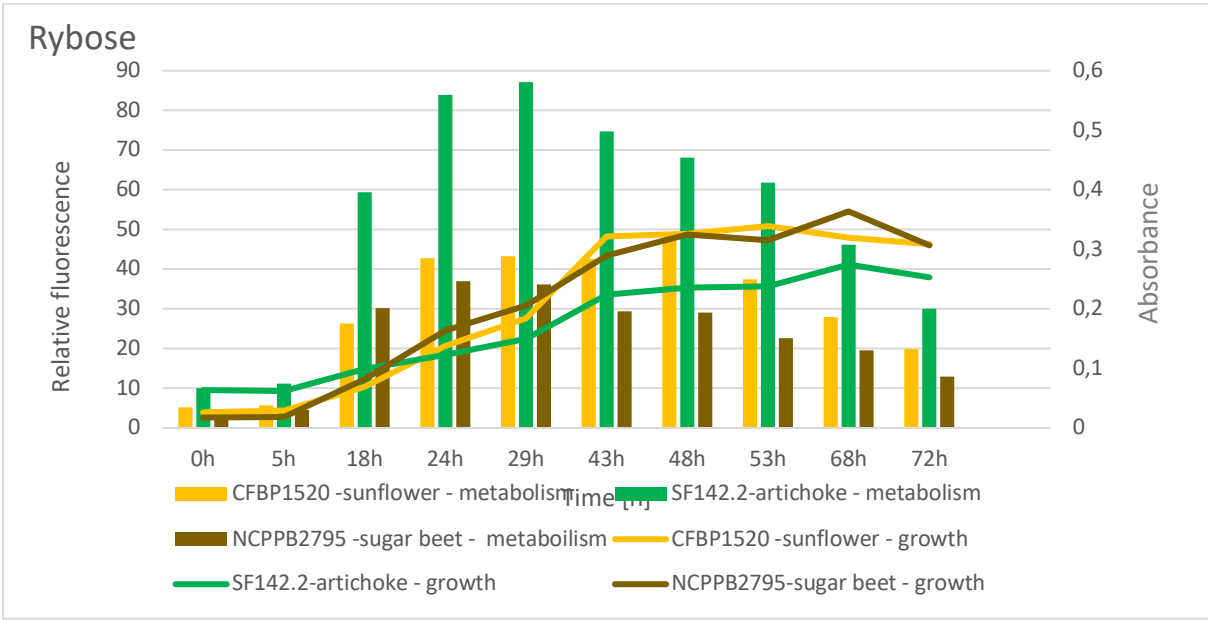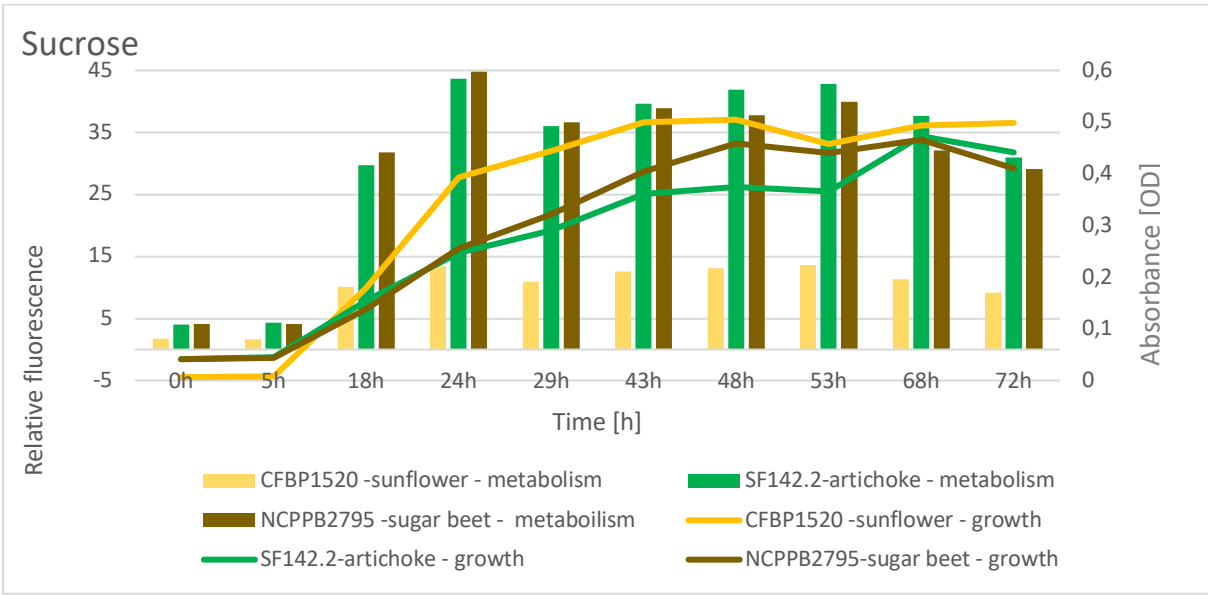

## Sorbitol

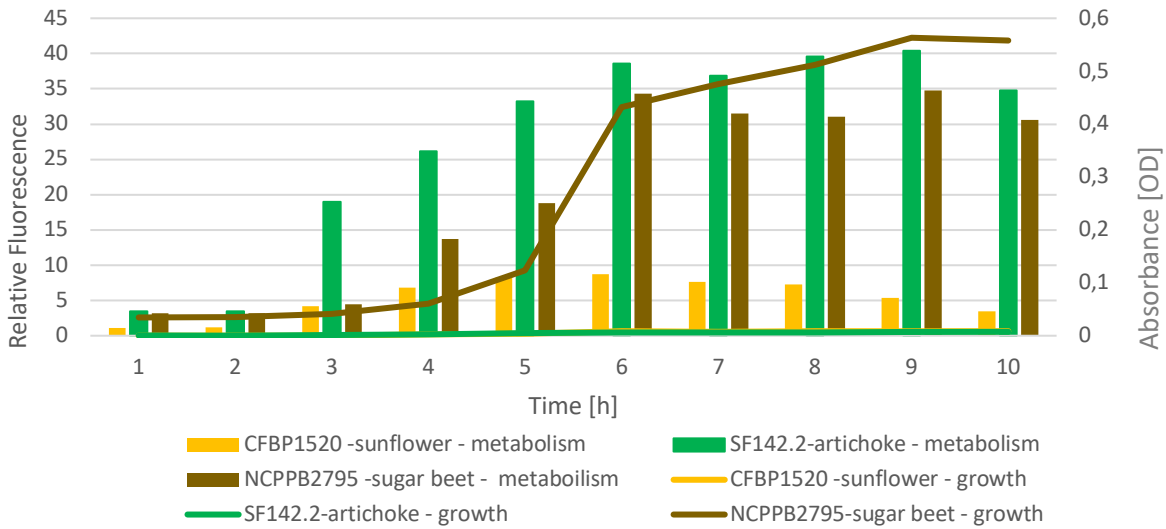

## Trehalose

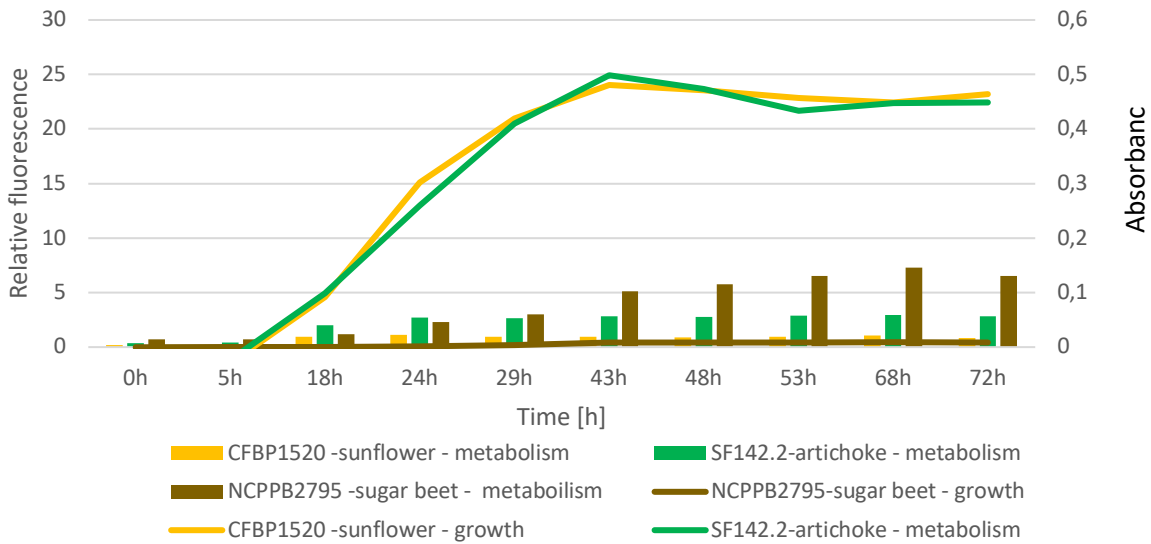

## Turanose

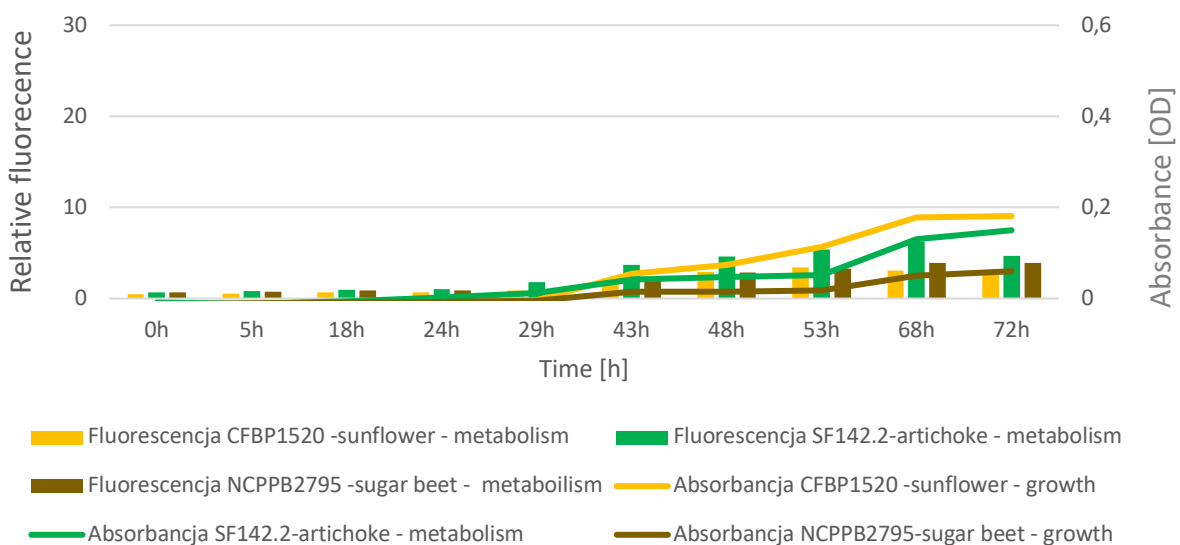

Supplement: Supplementary file 1 [file DataSheet_1.zip › Figure S3.pdf]

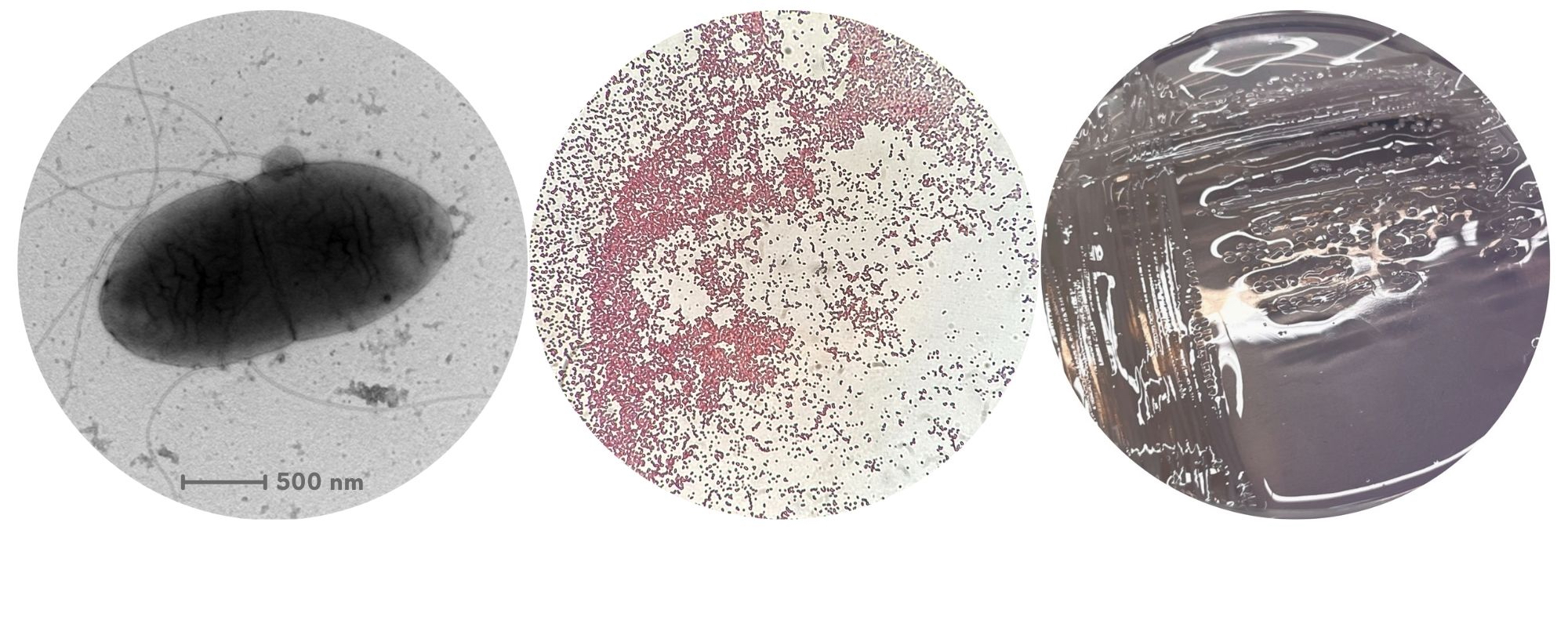

Supplement: Supplementary file 1 [file DataSheet_1.zip › Figure_S1_TEM.jpg]

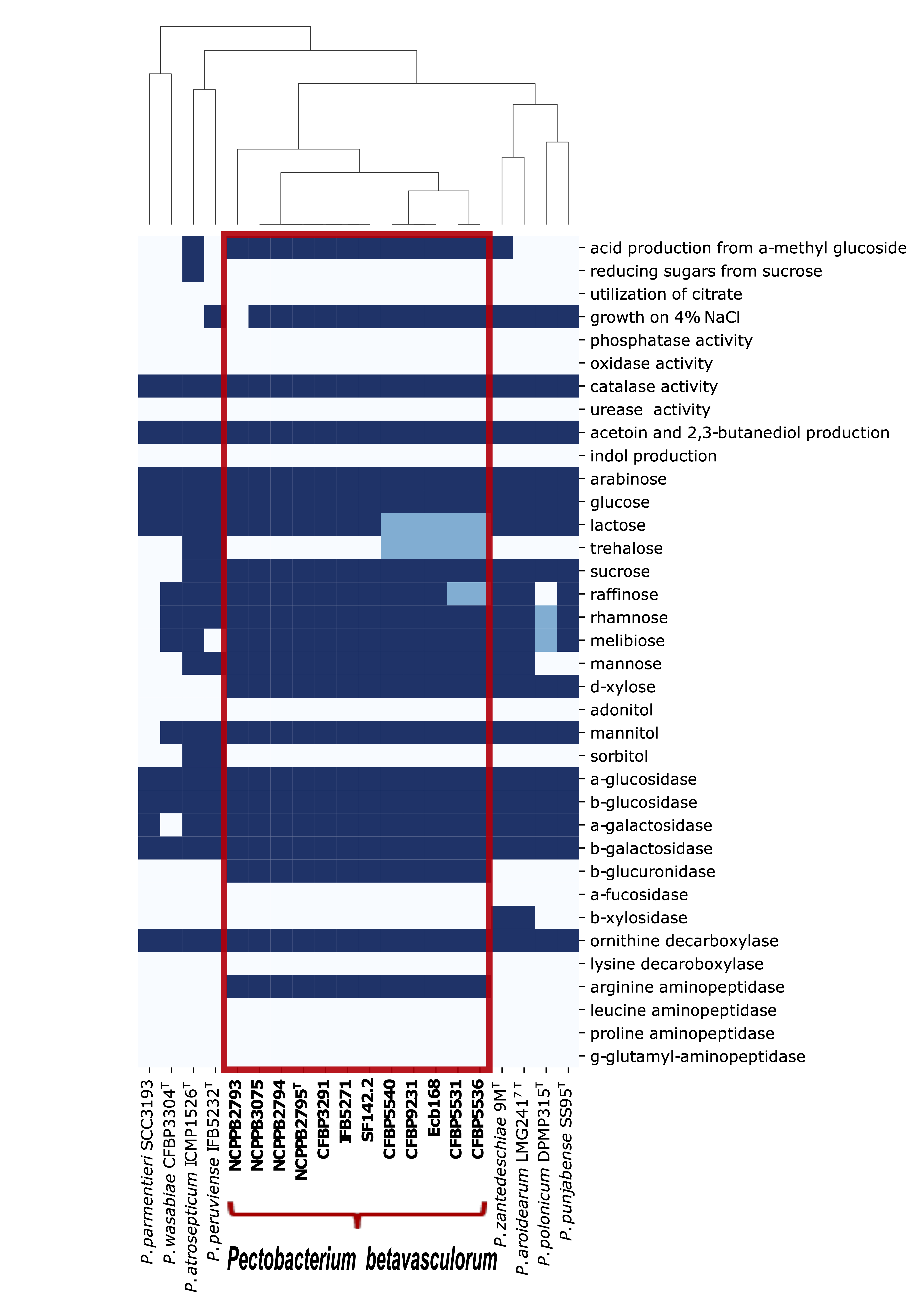

Supplement: Supplementary file 1 [file DataSheet_1.zip › Figure_S2_Phenotypic characteristic.jpg]

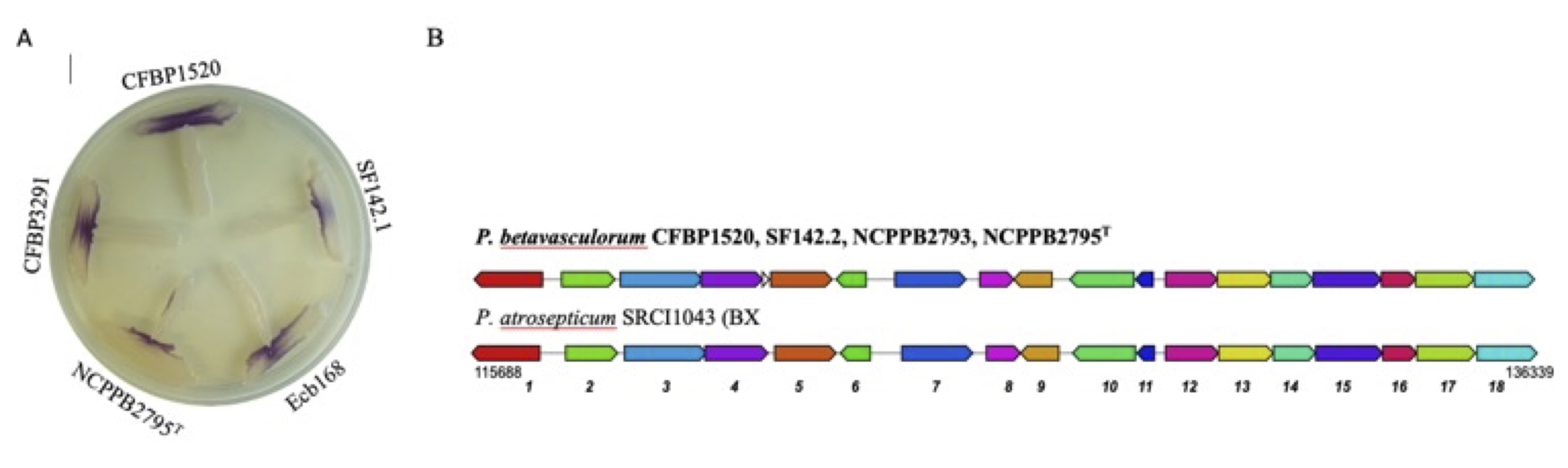

Supplement: Supplementary file 1 [file DataSheet_1.zip › Figure_S5 AHL.jpg]

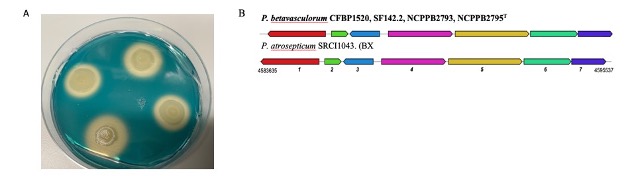

Supplement: Supplementary file 1 [file DataSheet_1.zip › Figure_S6 Siderophores.jpg]

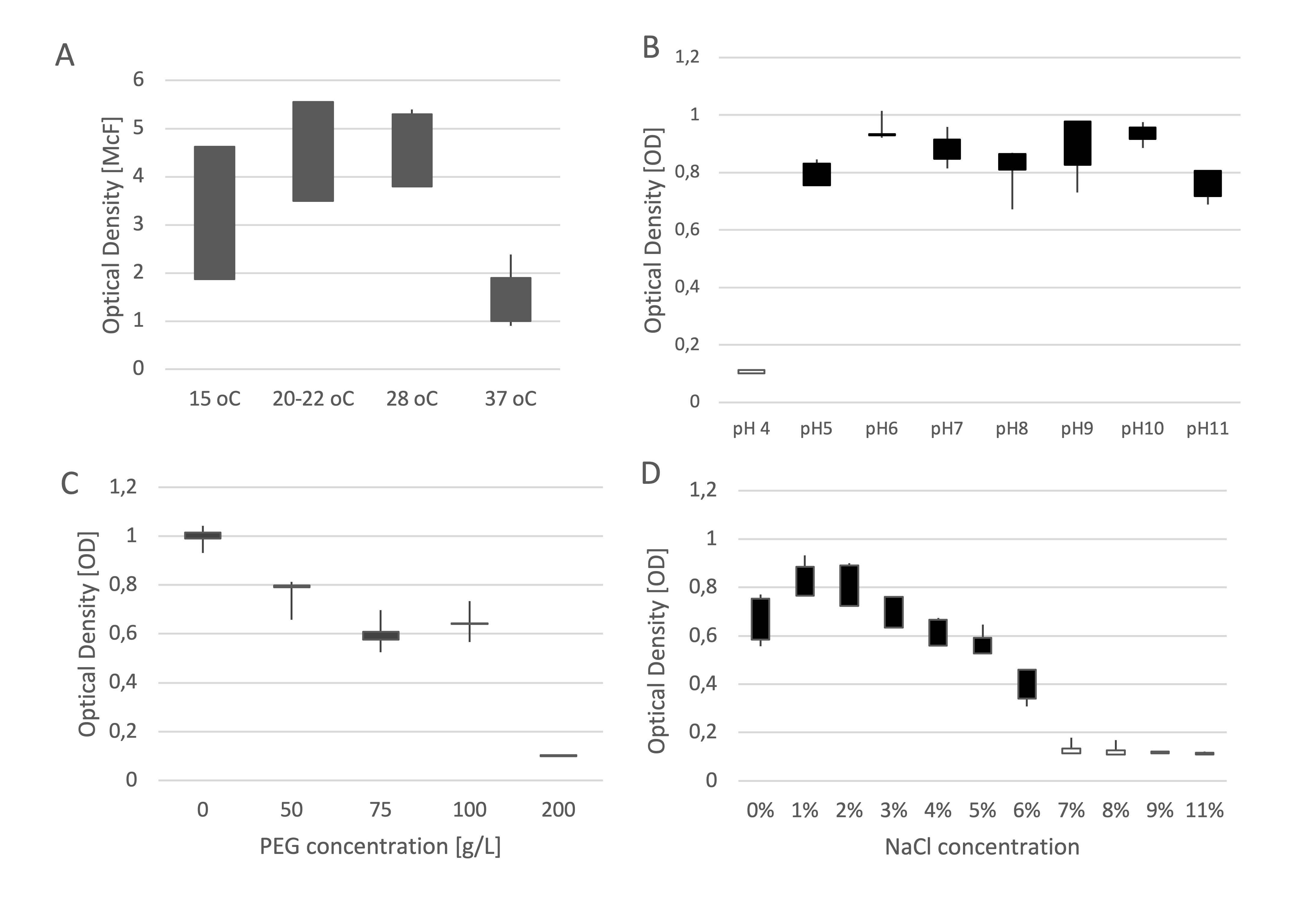

Supplement: Supplementary file 1 [file DataSheet_1.zip › Figure_S8_adaptation.jpg]

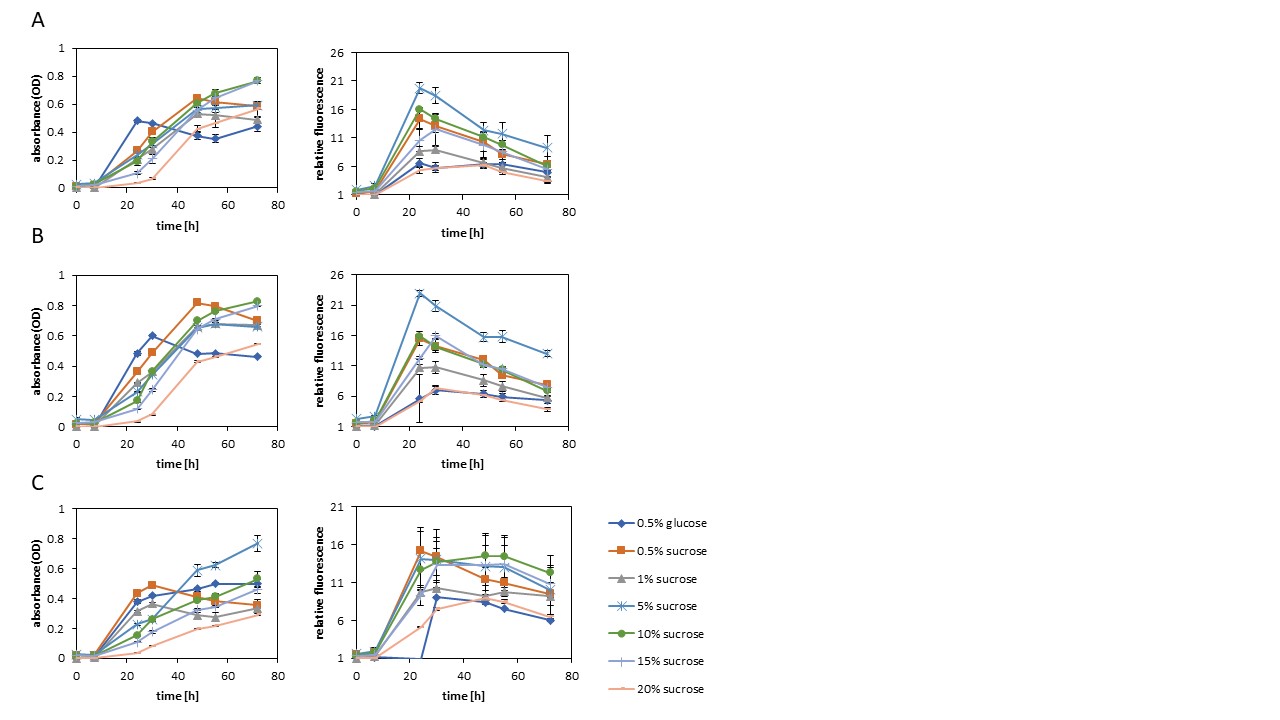

Supplement: Supplementary file 1 [file DataSheet_1.zip › Figure_S9_%sucrose.jpg]

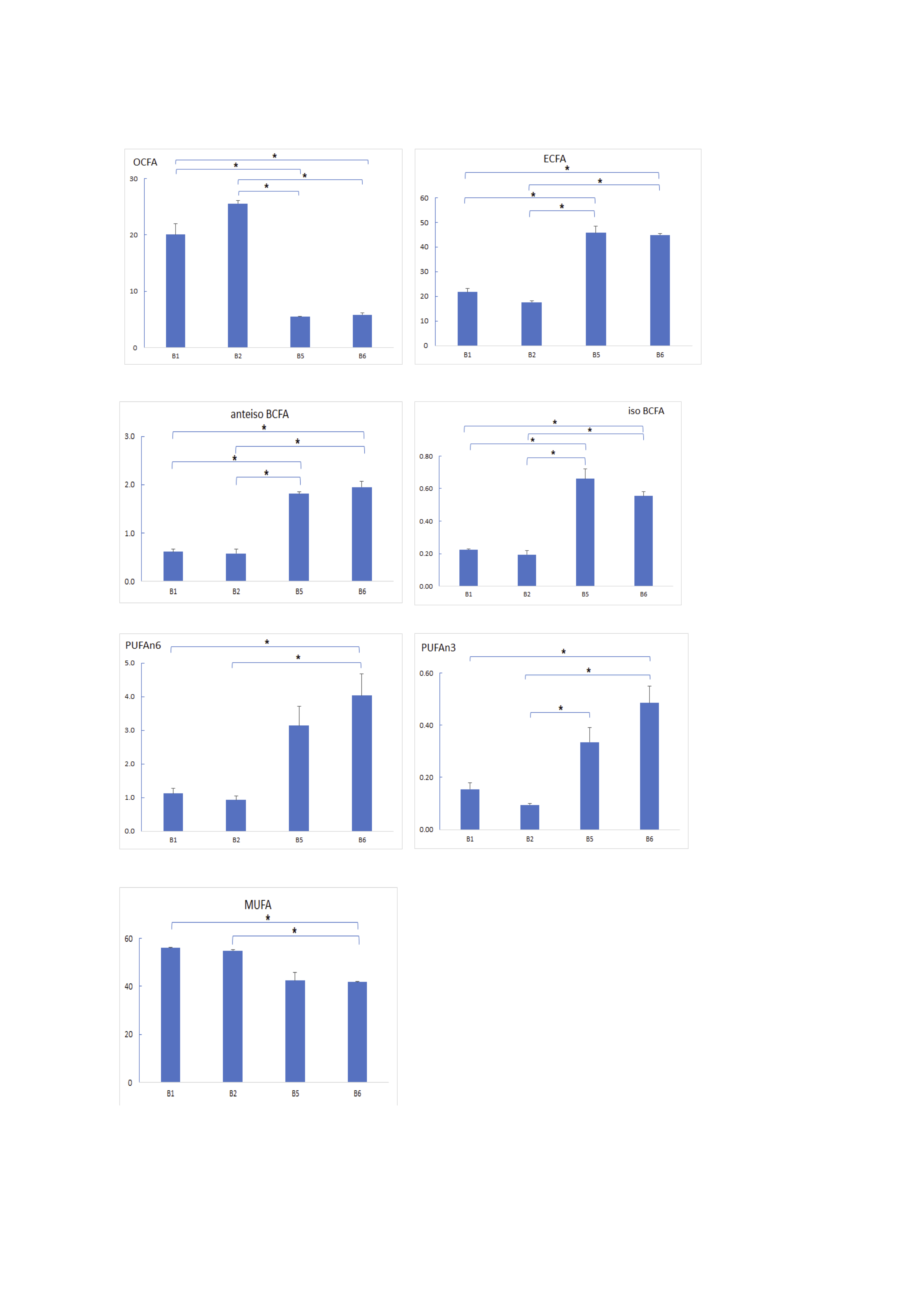

Supplement: Supplementary file 1 [file DataSheet_1.zip › Figure_S10_FA statistic.jpg]

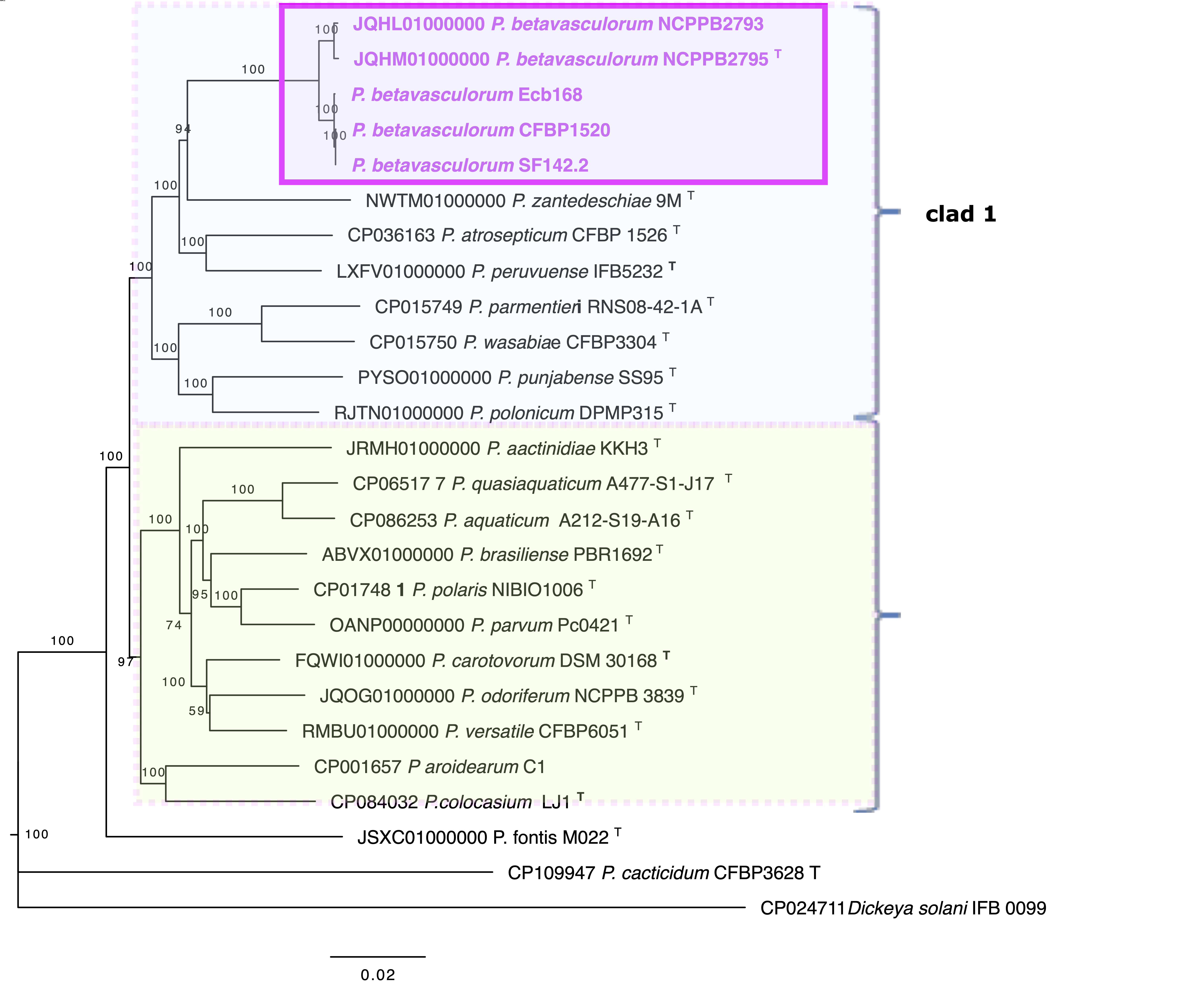

Supplement: Supplementary file 1 [file DataSheet_1.zip › Figure_S11-MLSA.jpg]

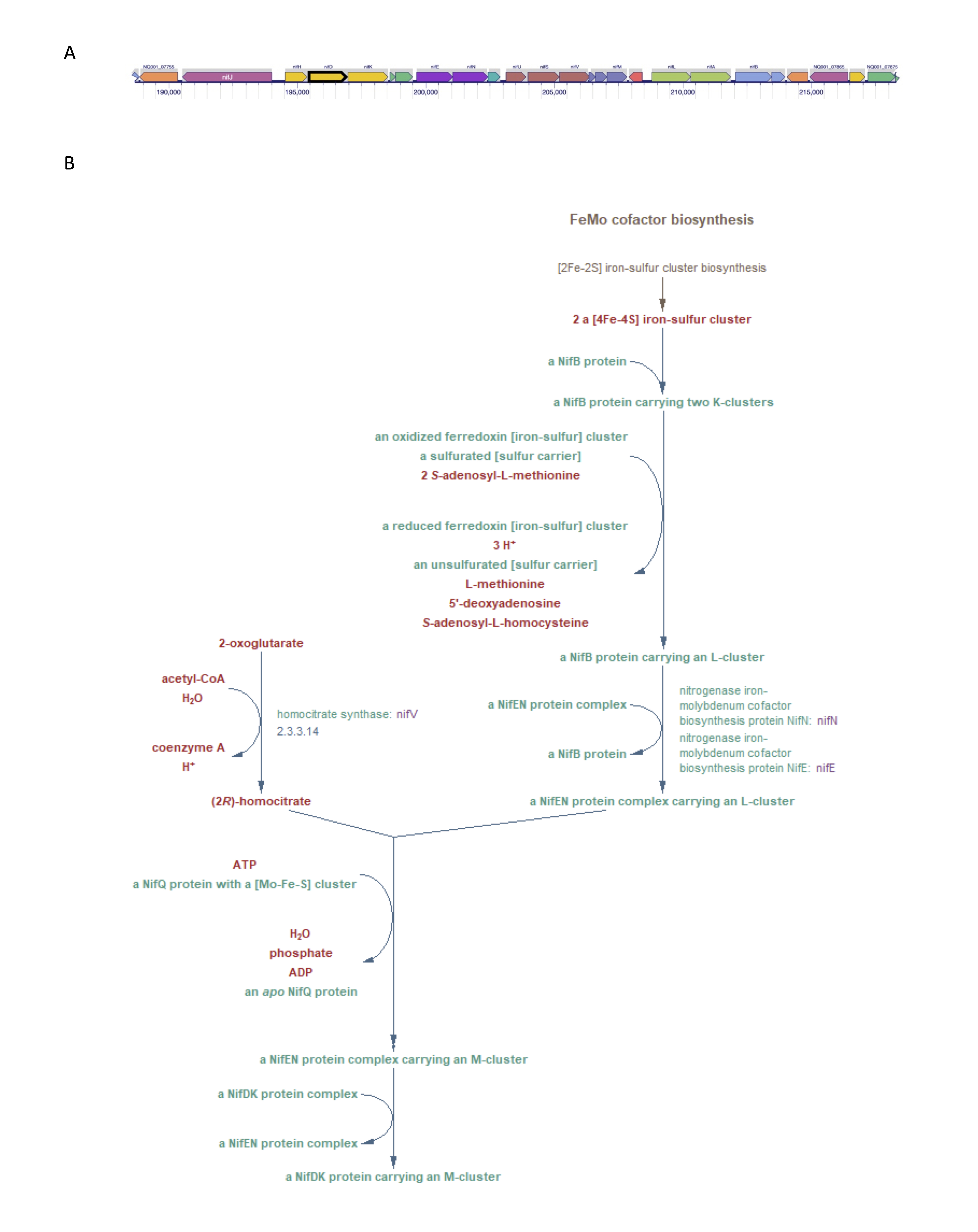

Supplement: Supplementary file 1 [file DataSheet_1.zip › Figure_S14_nif_operon.jpg]

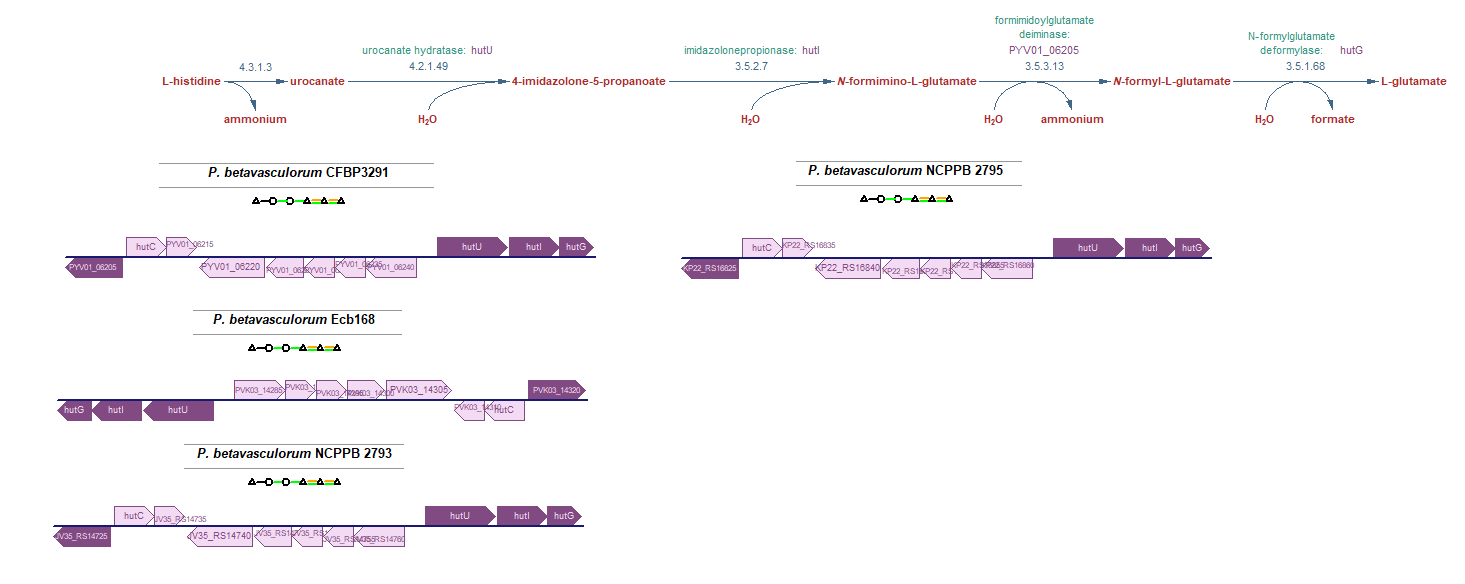

Supplement: Supplementary file 1 [file DataSheet_1.zip › Figure_S15_histidine_degradation_pathway.png]

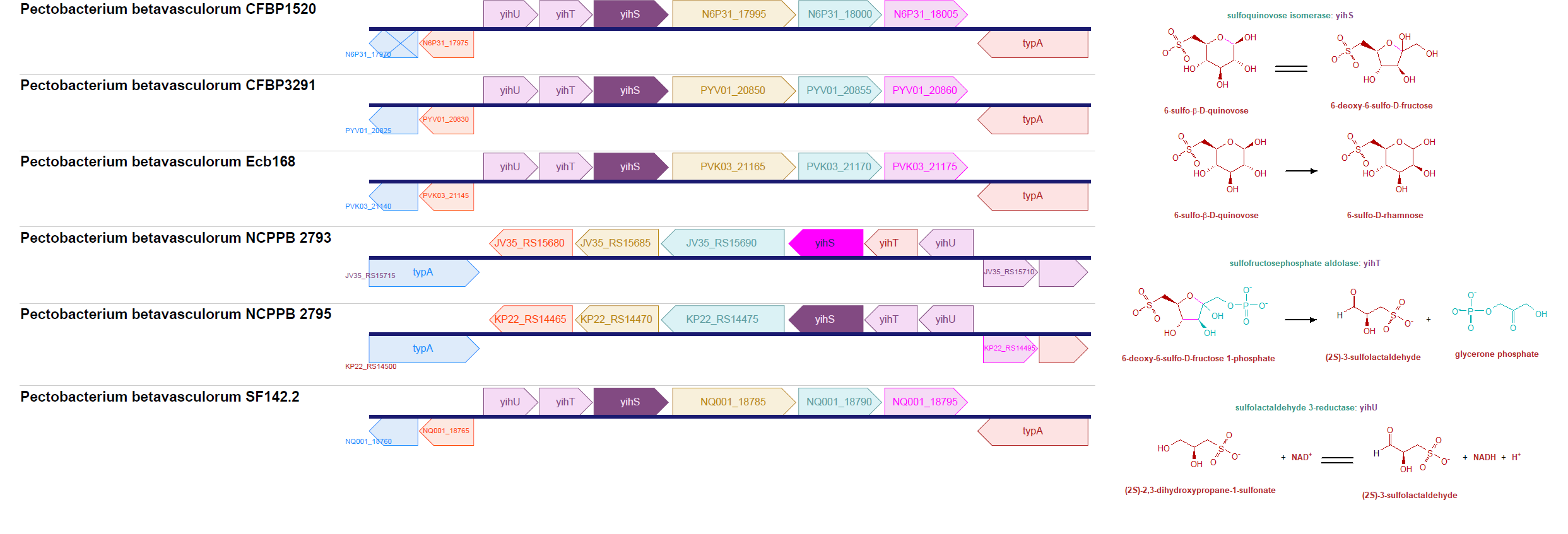

Supplement: Supplementary file 1 [file DataSheet_1.zip › Figure_S16_quinovose.png]

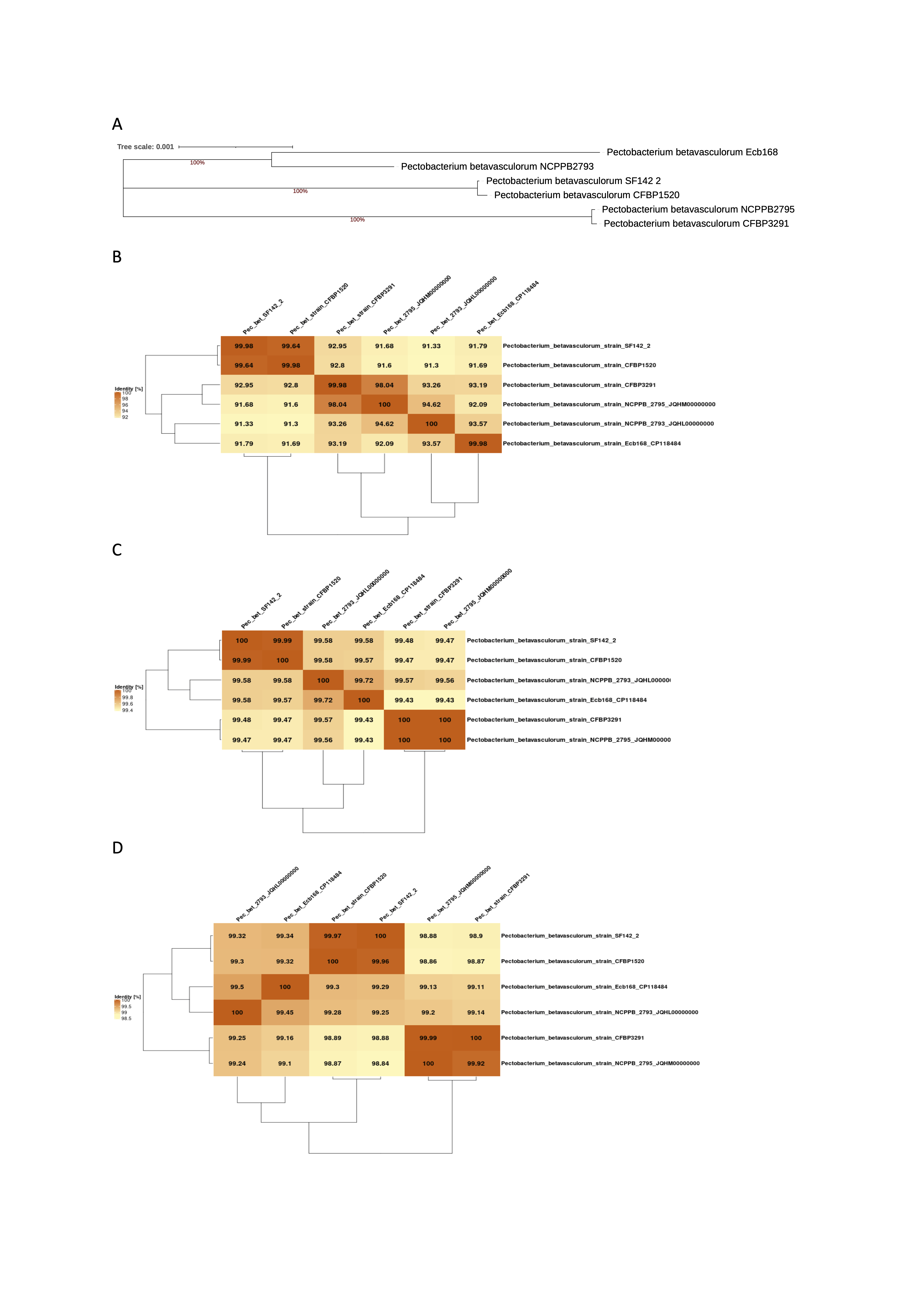

Supplement: Supplementary file 1 [file DataSheet_1.zip › FigureS12_Genomic_analyses_fin.jpg]

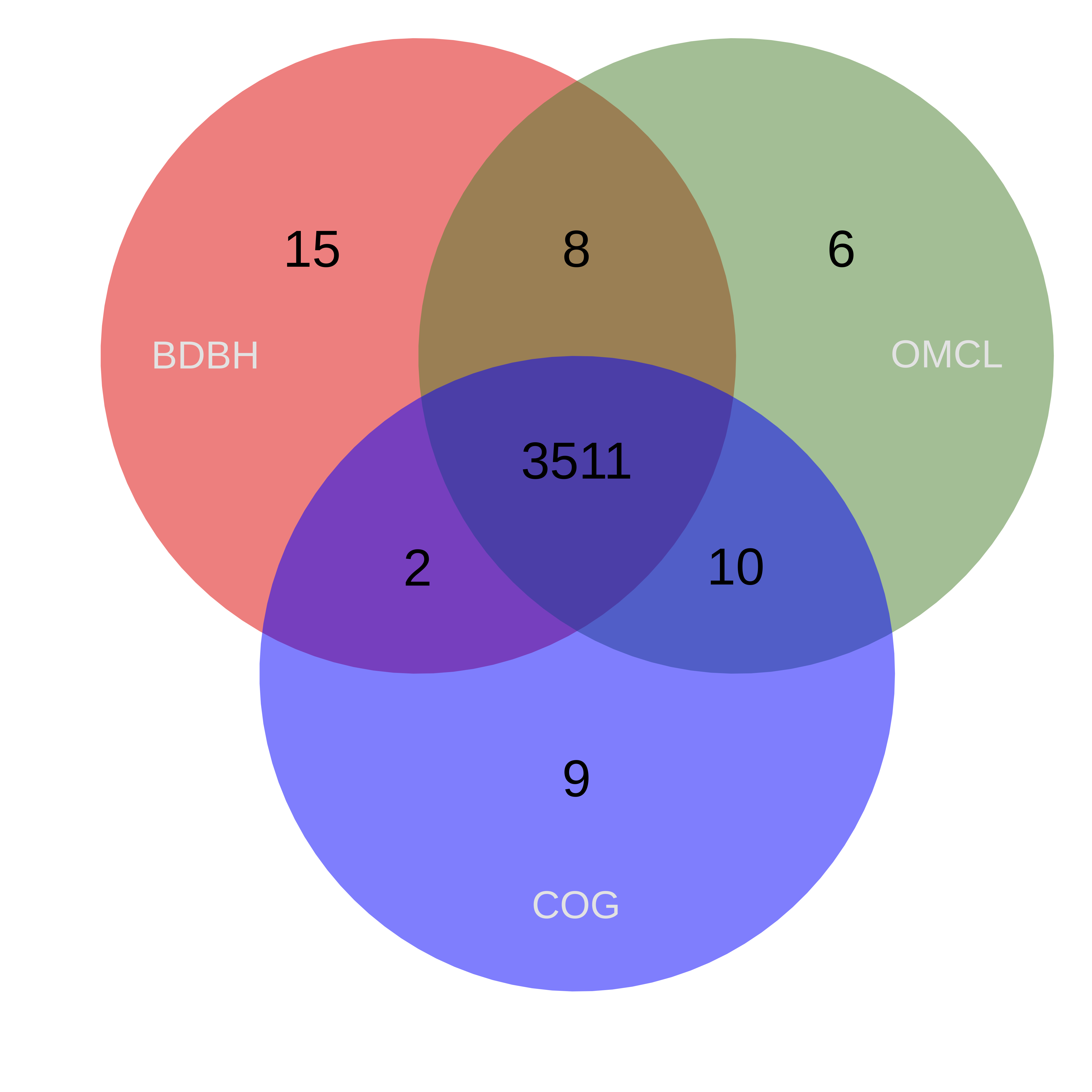

Supplement: Supplementary file 1 [file DataSheet_1.zip › Fogure_S13_venn_t0.jpg]
